# Beyond Floristic Quality: A Data-Driven Approach to Assessing Coastal Wetland Condition Using Plants

**DOI:** 10.1007/s13157-026-02094-z

**Published:** 2026-10-06

**Authors:** Giuseppe E. Fiorino, Simon T. Denomme-Brown, Hayley N. Rogers, Ian M. Smith, Kathryn L. Amatangelo, Matthew J. Cooper, Nicholas P. Danz, Robert W. Howe, Rachel E. Schultz, Bridget A. Wheelock, Douglas A. Wilcox, Greg P. Grabas

**Affiliations:** 1 Environment and Climate Change Canada, 4905 Dufferin Street, Toronto, ON M3H 5T4 Canada; 2 Department of Environmental Science and Ecology, SUNY-Brockport, 350 New Campus Drive, Brockport, NY 14420 USA; 3 Department of Biology, Grand Valley State University, Allendale, MI 49401 USA; 4 Department of Natural Sciences, University of Wisconsin-Superior, 1605 Catlin Avenue, Superior, WI 54880 USA; 5 Cofrin Center for Biodiversity, University of Wisconsin-Green Bay, Green Bay, WI 54311-7001 USA; 6 Institute for Great Lakes Research, Department of Biology, Central Michigan University, Mount Pleasant, MI USA

**Keywords:** Anthropogenic disturbance, Index of biotic condition, Laurentian Great Lakes, Marsh, Monitoring, Plant community

## Abstract

Plant communities are commonly used to assess ecological condition due to their sensitivity to local environmental factors and the practicality of conducting vegetation surveys. Using a large dataset (41,314 survey plots from 563 wetlands across 12 years), we developed and tested a new plant-based Index of Biotic Condition (pIBC) for coastal wetlands for each of the five Laurentian Great Lakes. The pIBC shares conceptual similarities with Floristic Quality Assessment (FQA) metrics but incorporates species-specific sensitivity and responsiveness to anthropogenic disturbance based on modeled probabilities of occurrence derived from field data. This distinguishes it from traditional FQA metrics, which rely on expert-assigned Coefficients of Conservatism and do not incorporate occurrence probability. We found that each lake-specific version of the pIBC consistently predicted anthropogenic disturbance, as indicated by water quality and surrounding land use. Additionally, comparisons with seven other plant-based metrics showed that the pIBC generally provides greater within-lake sensitivity to water-quality and land-use impacts, providing higher-resolution insight into spatial and temporal variation at the lake scale, which is often the most relevant scale for management decisions. The pIBC was also applicable across wetlands of different hydrogeomorphic types and under different water-level conditions. The pIBC’s strong performance suggests that it is well-suited for assessing coastal wetland condition across sites and within sites through time. Overall, this new index is a conceptually grounded and statistically robust tool for conservation practitioners that is easy to calculate and interpret.

**Supplementary Information:**

The online version contains supplementary material available at https://doi.org/10.1007/s13157-026-02094-z.

## Introduction

To conserve or restore the natural environment effectively, conservation practitioners must be able to distill ecosystem condition from complex information gathered during field surveys. It is, therefore, unsurprising that indices of ecological integrity or condition – tools that summarize detailed field data, often into a single number – have become increasingly popular over recent decades (e.g., Burton et al. 1999; Cooper et al. 2018; Grabas et al. 2012; Howe et al. 2007a). One widely adopted tool, especially in North America, is Floristic Quality Assessment (FQA) and its associated metrics (the Floristic Quality Index, mean Coefficient of Conservatism, and numerous variants; e.g., Kutcher and Forrester 2018). The foundation of FQA is based on numerical scores (Coefficients of Conservatism) that are assigned to individual plant species within a region by experienced botanists. Coefficients of Conservatism are based on a species’ fidelity to high-quality, undisturbed habitats, with higher values indicating species that are less likely to be present in disturbed conditions (Swink and Wilhelm 1994; Zinnen et al. 2025).

Despite its popularity, FQA has limitations and has been subject to criticism (Bried et al. 2013; DeBerry and Perry 2015; Matthews et al. 2015; Spyreas 2019). For instance, Coefficients of Conservatism (C) values are based on professional judgment rather than being empirically derived from species’ actual sensitivities to site quality (with some exceptions; e.g., Bried et al. 2018; Bried et al. 2025), and may be biased by species or habitat rarity (although there is little evidence that this is problematic; Matthews et al. 2015; Spyreas 2019). Additionally, C values are assigned to reflect a species’ relationship with disturbance in a specific region and may differ for ecological or methodological reasons among regions (e.g., use of different criteria or protocols when assigning C values; Spyreas 2019). Individual species may also differ in their tolerance to disturbance at smaller scales or for specific ecosystems, but it is impractical to have a set of C values for all regions and ecosystems of interest. Regardless of these concerns, FQA remains popular because it is simple, easy to use, and often agrees with subjective assessments of site quality (Spyreas 2014, 2019).

One alternative index that shows promise in addressing some of FQA’s shortcomings is the Index of Biotic Condition (IBC), first developed for birds by Howe et al. (2023). The IBC approach involves objectively determining species’ relationships with anthropogenic disturbance while maintaining the simplicity of FQA. As a result, it captures the conceptual intent of FQA in summarizing species-level responses to environmental conditions, but unlike FQA, it relies on quantitative, empirical data specific to the region and ecosystem being studied. To extend this methodology to plants, an extensive dataset would be required to quantify the responses of species to an environmental reference gradient (Howe et al. 2023). An ideal dataset for this purpose has been assembled by the Great Lakes Coastal Wetland Monitoring Program (CWMP), a U.S.-Canadian bi-national collaboration of federal agencies, states, and academic partners that samples coastal wetlands throughout North America’s Laurentian Great Lakes (hereafter “Great Lakes”) to provide information on coastal wetland condition (https://greatlakeswetlands.org/; Uzarski et al. 2017; Uzarski et al. 2019). Since the program began in 2011, over 900 unique wetland sites have been surveyed, which offers an unprecedented opportunity to develop improved indices of wetland condition (e.g., Cooper et al. 2018; Dybiec et al. 2020; Howe et al. 2023). This extensive data resource can also be used to expand on previous work validating the effectiveness of FQA in Great Lakes coastal wetlands (e.g., Bourdaghs et al. 2006).

Great Lakes coastal wetlands provide immense ecological, economic, and cultural value by serving as essential breeding and migratory habitat for wildlife, providing spawning and nursery habitat for fish (supporting both commercial harvest and recreational angling), improving water quality, among other ecosystem functions and services (reviewed in Sierszen et al. 2012). The Great Lakes also contain some of the world’s most distinctive and important wetlands, including nine Ramsar Wetlands of International Importance (https://rsis.ramsar.org/*).* Unfortunately, the Great Lakes basin has experienced extensive wetland loss (Ducks Unlimited Canada 2010; Amani et al. 2022) and widespread degradation from land-use changes, pollution, water-level regulation, invasive species, and climate change (Mortsch 1998; Danz et al. 2007; Allan et al. 2013). Given these pressures, it is imperative that tools exist to quantify coastal wetland condition accurately. Ideally, such tools allow practitioners to identify high-quality wetlands, track change over time, evaluate restoration success, and identify threats to inform conservation and management.

In this study, we developed a plant-based Index of Biotic Condition (pIBC) for coastal wetlands for each of the Laurentian Great Lakes following an approach similar to the one used by Howe et al. (2023) for birds. We then validated the lake-specific versions of the pIBC and compared them to seven other plant-based metrics, including four floristic quality metrics. Many traditional biotic indices do not perform as well in the southern lakes (lakes Erie and Ontario) as they do in the northern lakes (lakes Superior, Michigan, and Huron) (e.g., Dybiec et al. 2020), possibly due to the complicating factor of water-level management on Lake Ontario and the extensive history of land-use alteration in the southern basin that has left few, if any, pristine wetlands. Additionally, many biotic indices that perform adequately at large scales (e.g., among lakes) seem to lack resolution within lakes (based on unpublished CWMP data), which is the scale that is often most relevant for conservation practitioners and land managers. Accordingly, our goals were to develop a new index that (1) objectively links plant species occurrences to known environmental stressors, (2) offers improved performance in the Great Lakes compared to other metrics, (3) maximizes within-lake resolution, and importantly, (4) is simple to compute and interpret. We also tested whether each version of the pIBC was robust to changes in water levels (Wilcox et al. 2002) and whether it was suitable for use in wetlands of different hydrogeomorphic types (Albert et al. 2005).

## Materials and Methods

### Study Area

We used data collected from 2011 to 2022 by the Great Lakes Coastal Wetland Monitoring Program (https://greatlakeswetlands.org/; Uzarski et al. 2017; Uzarski et al. 2019). The pool of study sites was all coastal wetlands in the Great Lakes basin greater than 4 ha in size with a permanent or periodic surface-water connection to an adjacent Great Lake or connecting river system. Coastal wetlands were selected for sampling using a random sampling protocol stratified by (1) wetland hydrogeomorphic type (lacustrine, riverine, barrier protected; Albert et al. 2005), (2) region (northern or southern; Danz et al. 2005), and (3) lake (with connecting channels included as part of the downstream Great Lake) (Uzarski et al. 2017, 2019). We sampled roughly 20% of all wetlands in each stratum each year, so that nearly all coastal wetlands within the Great Lakes basin meeting the selection criteria were sampled at least once every five years. In addition, we resampled 10% of wetlands according to a rotating panel design. Sampled wetlands were dominated by emergent, herbaceous vegetation and shallow water (< 2 m deep) containing floating and/or submerged vegetation. We used data from 563 wetlands that were sampled an average of 2.3 times over the 12-year study period, representing 1,273 wetland-year sampling events. The number of wetlands sampled for vegetation in each lake was 73 for Lake Superior (155 sampling events), 87 for Lake Michigan (197), 194 for Lake Huron (456), 66 for Lake Erie (150), and 143 for Lake Ontario (315).

### Plant Surveys

Vegetation sampling for each wetland occurred between June and August along three transects established perpendicular to the depth contours of the wetland that crossed through selected vegetation zones. Major vegetation zones were defined as the wet meadow zone, emergent vegetation zone, and the submerged and floating aquatic vegetation zone. Each transect consisted of five quadrat sampling points (i.e., survey plots) per vegetation zone that were evenly spaced and centered between the boundaries of each vegetation zone. Up to 15 quadrat sampling points per transect were sampled if all vegetation zones were present. Vegetation was surveyed in 1m^2^ quadrats at each sampling point along each transect for a total of 15–45 quadrats per wetland (depending on number of zones present). A width of 11 m was used as a zone width threshold because that was the smallest width to accommodate five 1m^2^ quadrats with a 1 m distance between quadrats and the zone boundaries. If the width of a vegetation zone was less than 11 m, a perpendicular transect was established at the midpoint of the zone along the original transect, and quadrats were placed at 5 m intervals along the perpendicular transect in the narrow vegetation zone. The presence and percent cover for each plant species were recorded for each quadrat. Plant species were identified to the species-level where possible, with the exception of certain non-vascular species (e.g., *Chara* spp. and *Nitella* spp.).

### Environmental Condition Gradient

The first step in developing the pIBC was to quantify the response of individual plant species to an independently-derived gradient of environmental condition (Howe et al. 2007b, 2023). The environmental condition gradient was defined by a collection of independent environmental variables associated with the wetland sampling points, yielding an objective score ranging from very low environmental quality to very high environmental quality, but not based at all on wetland plant occurrences. We used the Coastal Wetland Monitoring Program’s Water Quality and Land Use (WQLU) Index (formerly called Sum-Rank; Uzarski et al. 2017; Harrison et al. 2020) as our measure of environmental condition because it incorporates components of water quality and land cover that are likely to influence the structure of coastal wetland plant communities at different spatial scales directly and indirectly. This index is also strongly correlated with other common measures of disturbance for Great Lakes coastal wetlands, including AgDev (*r* = 0.80), a stress index based on the percentage of agricultural land within a watershed, the percentage of urban land use within a watershed, population density, and road density (Host et al. 2019), and the Water Quality Index (*r* = 0.89), a stress index based solely on water chemistry (Chow-Fraser 2006; Eq. 8). The WQLU Index was calculated based on ten in situ water quality variables (water clarity, specific conductance, total nitrogen, nitrate-nitrite-N, ammonium-N, total phosphorus, soluble reactive phosphorus, chlorophyll-a, dissolved oxygen, and pH), eight land-cover variables (proportions of agriculture, development, natural vegetation, and wetlands within 1 km and 20 km buffers from each wetland), and one principal component (PC1) derived from a principal component analysis of all water quality and land-cover variables (scaled such that higher values indicated better condition) (Uzarski et al. 2005). Values for each variable were rank-transformed such that the rank order was ordinal to the inferred degree of anthropogenic disturbance (with the greatest rank for each variable indicating the least disturbance). Ranks from all 19 variables were then summed, and each summed WQLU Index value was scaled from 0 to 10, with higher values indicating better environmental condition.

Water quality sampling was conducted at three replicate locations within dominant plant growth forms in each wetland where vegetation sampling occurred. Plant growth forms were defined as patches of vegetation dominated by species sharing similar physical structure (e.g., a mixed patch of *Nuphar variegata* and *Nymphaea odorata* were considered a single growth form). The most common growth forms sampled were cattail (*Typha* spp.), submerged aquatic vegetation (e.g., *Potamogeton*,* Vallisneria*,* Ceratophyllum* spp.), lily (e.g., *Nymphaea*,* Nuphar*,* Nelumbo* spp.), dense bulrush (*Schoenoplectus* spp.), sparse bulrush, wet meadow (e.g., *Carex*,* Juncus* spp.), and common reed (*Phragmites australis*). In situ water quality variables (dissolved oxygen, pH, and specific conductance) were measured at the mid-depth of the water column using a water quality sonde. Water samples were collected from each sample location and stored for laboratory analysis of total nitrogen, total phosphorus, ammonium-N, nitrate-nitrite- N, soluble reactive phosphorus, and chlorophyll-a following standard analytical methods (APHA 2005). A transparency tube with Secchi disk was used to measure water clarity (see Uzarski et al. 2017 for additional detail). For wetlands where multiple plant growth forms were sampled, water quality parameters were averaged across growth forms, and these average values were used to calculate site-level WQLU Index values for each year a wetland was sampled. Land-cover variables were calculated using the 2010 North American Land Cover 30 m dataset from the North American Land Change Monitoring System (NALCMS) (https://www.cec.org/north-american-environmental-atlas/land-cover-2010-landsat-30 m/).

### Linking Species Occurrences to Environmental Condition

For each plant species, we generated best-fit “curves,” referred to as biotic response (BR) functions, relating the probability of occurrence of each species to the WQLU Index environmental condition gradient. Shapes of the curves across the environmental gradient were either monotonically increasing (the upward part of the normal curve), monotonically decreasing (the downward part of the normal curve), or unimodal with complete or truncated tails. To quantify these responses, we applied an algorithm developed by Howe et al. (2023) to select the best-fit parameters of a normal (bell-shaped) curve. The curve was defined using the R function *dnorm* (R Core Team 2023), and the parameters were estimated via the *nlminb* function (Gay 1990).

**Fig. 1:**
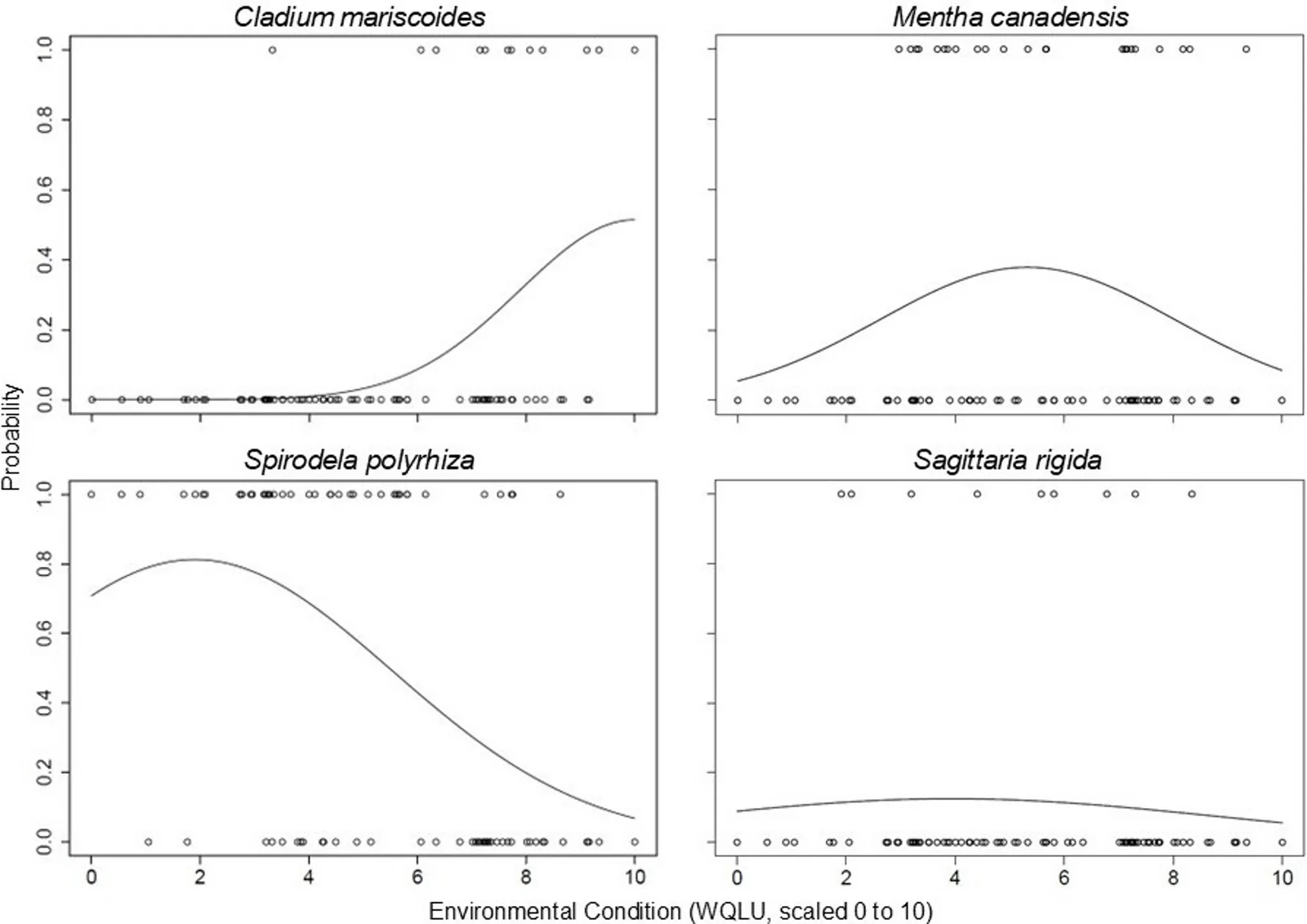
Examples of biotic response curves for four species for Lake Michigan (see Online Resource 1 for biotic response curves for all species for each lake). Circles represent the presence (1.0) or absence (0.0) of each species in quadrats sampled for each coastal wetland site between 2011 and 2022. *Cladium mariscoides* (top left) probability of occurrence was greatest in wetlands with high WQLU Index values (less disturbed), *Spirodela polyrhiza* (bottom left) was greatest in wetlands with low WQLU (more disturbed), *Mentha canadensis* (top right) was greatest at intermediate WQLU, and *Sagittaria rigida* (bottom right) showed no appreciable relationship with WQLU

We divided our dataset into lake-specific training datasets (to develop/calibrate the index) and testing datasets (to validate the index on a dataset that was separate from the dataset used for development/calibration). Our training datasets consisted of 75% of sites that were sampled at least two times over the study period; if these sites were sampled more than twice, then two sample years were randomly selected to ensure that sites that were sampled more frequently were not overrepresented (36 sites [72 sampling events] for Lake Superior, 42 [84] for Lake Michigan, 104 [208] for Lake Huron, 39 [78] for Lake Erie, 76 [152] for Lake Ontario). Our testing datasets consisted of the remaining 25% of sites that were sampled at least two times over the study period, as well as any sites that were sampled only once during the study period (37 sites [59 sampling events] for Lake Superior, 45 [74] for Lake Michigan, 90 [159] for Lake Huron, 27 [48] for Lake Erie, 67 [107] for Lake Ontario). The training datasets were used to generate lake-specific BR functions for all native species that occurred at least five times in those datasets (124 species for Lake Superior, 141 for Lake Michigan, 251 for Lake Huron, 59 for Lake Erie, 124 for Lake Ontario; Fig. 1, Online Resource 1).

### Plant Index of Biotic Condition (pIBC)

Site-level pIBC values were computed by aggregating species “weights” (*w*_*i*_), which were calculated using parameters derived from BR functions for species encountered during standardized field surveys (see https://doi.org/10.5061/dryad.0cfxpnwg2 for weights for all species for each lake and a pIBC calculator). A weight for each native species was calculated as the product of two parameters: (1) the species optimum, $\:\stackrel{-}{x}$_*i*_ (the value of WQLU at which probability of occurrence is highest; the peak of the BR function), and (2) the difference between *p(*$\:\stackrel{-}{x}$_*i*_*)*_*i*_ (the predicted probability of occurrence at the optimum) and *p(0)*_*i*_ (the predicted probability of occurrence under the most degraded conditions [WQLU = 0]). The first parameter ($\:\stackrel{-}{x}$_*i*_) reflects the sensitivity of the species to the environmental condition gradient; in general, the more sensitive the species, the larger the optimum value. The second parameter [*p(*$\:\stackrel{-}{x}$_*i*_*)*_*I*_*– p(0)*_*i*_] reflects the responsiveness of the species to change in the environmental condition gradient; in general, the more responsive the species, the larger the difference between the maximum probability of occurrence and the probability of occurrence when environmental condition is most degraded. Hypothetically, a species with a maximum weight (*w*_*i*_ = 10) would have peak probability of occurrence when WQLU = 10 ($\:\stackrel{-}{x}$_*i*_ = 10) and would occur at 100% of sites when WQLU = 10 and 0% of sites when WQLU = 0 [*p(*$\:\stackrel{-}{x}$_*i*_*)*_*i*_*– p(0)*_*i*_ = 1]. All non-native species were assigned a weight of zero, as their presence inherently reflects anthropogenic introduction and departure from reference conditions, which was consistent with the conceptual basis of the index. We considered retaining empirically derived weights for non-native species. Although preliminary calculations of weights for non-natives were generally low (median = 0.50), a small subset received comparatively higher weights, reflecting that some non-native species occur more frequently in sites otherwise characterized by better conditions. However, these cases likely reflect factors such as introduction history and dispersal pathways that are not fully captured by the WQLU environmental condition gradient. In addition, retaining empirically derived weights for such taxa could give the misleading impression that their presence is desirable. Importantly, preliminary testing (not shown here) indicated that this decision to assign non-native species a weight of zero did not meaningfully affect the performance of the pIBC.

The pIBC value for a given wetland ($\:{pIBC}_{Lake,i}$) was calculated as the mean weight of all plant species observed multiplied by the square-root of the number of species with weights, following the same general structure of the Floristic Quality Index (mean C multiplied by √N; Swink and Wilhelm 1994):

$$\:{pIBC}_{Lake,i}=\:{\stackrel{-}{w}}_{Lake,i}\:\surd\:{N}_{i}$$

where $\:{\stackrel{-}{w}}_{Lake,i}$ is the mean weight and $\:{N}_{i}$ is the number of species with weights for wetland *i* within a given lake.

We note that this computation differs from Howe et al. (2023), who calculated their bird IBC as the sum of weights for all species present at a site. Calculating the pIBC in this modified manner integrates species richness but avoids scenarios where sites with numerous low-weight species outscore sites with fewer high-weight species. This was particularly important for the pIBC because of the substantial number of species included in the different versions of the index (59 to 251 depending on the lake, compared to 14 species used for the bird IBC).

### Index Validation

To validate each lake-specific version of the pIBC, we fit linear mixed-effects models (Gaussian family) with the WQLU Index as the response variable, pIBC, dataset (whether the site/sampling event belonged to the training or testing dataset), and their interaction as fixed effects, and site and year as random effects. Each lake-specific pIBC was considered validated if there was (1) a non-statistically significant interaction between pIBC and dataset (indicating that the relationship between pIBC and WQLU was not significantly different between training and testing datasets), and (2) a statistically significant positive relationship between pIBC and WQLU. Although the models could have been fitted with WQLU Index as the predictor and pIBC as the response, we modelled WQLU as the response because our objective was to validate the pIBC rather than to model the ecological process (i.e., that disturbance shapes plant communities). In this validation framework, we assessed whether the pIBC, derived solely from plant community data, recovered known gradients of anthropogenic disturbance measured independently through water quality and land use. The inclusion of the dataset predictor and its interaction with pIBC was intended to ensure that validation was independent of the weight calculation process and to evaluate whether the pIBC performed consistently on previously unseen sites.

### Comparison to Other Metrics

We compared the performance of each lake-specific version of the pIBC to seven other vegetation metrics by evaluating their relationships with the WQLU Index (Uzarski et al. 2017; Harrison et al. 2020) within each lake. The vegetation metrics were: (1) mean coefficient of conservatism (mean C), (2) floristic quality index (FQI) (Swink and Wilhelm 1994), (3) cover-weighted mean C, (4) cover-weighted FQI (Bourdaghs et al. 2006), (5) a recently developed multi-metric index of biotic integrity (IBI) for Great Lakes coastal wetlands (Dybiec et al. 2020), (6) native species richness, and (7) invasive species cover. All metrics were calculated by aggregating survey data from all quadrats sampled at a site, except for invasive species cover, which was calculated as a quadrat-level average because preliminary analyses showed a stronger relationship between quadrat-level average cover and WQLU than site-level cumulative cover. For the FQA metrics, non-native species were included and assigned C values of zero. We elected to include non-native species in these metrics because many are widespread and often among the dominant taxa in many Great Lakes coastal wetlands (Trebitz and Taylor 2007). This approach was also consistent with our pIBC methodology (assigning weights of zero to non-native species). C values assigned to native species were based on the Floristic Quality Assessment System for Southern Ontario (for lakes Erie and Ontario) (Oldham et al. 1995) and the Michigan Floristic Quality Assessment Database (for lakes Superior, Michigan, and Huron) (Reznicek et al. 2014).

Using the full dataset (training, testing, and sampling events originally omitted from the training dataset), we fit a series of linear mixed-effects models for each lake (Gaussian family) with the WQLU Index as the response variable, one of the eight vegetation metrics as a fixed effect, and site and year as random effects. The full dataset was used to maximize statistical power, which was justified because, to validate the index, we demonstrated that the relationship between each lake-specific version of the pIBC and WQLU was not significantly different between training and testing datasets. We then assessed the relative performance of the eight candidate models (representing each vegetation metric) for each lake by comparing Akaike Information Criterion corrected for small sample size (ΔAICc) and marginal R^2^ (R^2^_m_; Nakagawa and Schielzeth 2013) values. This mixed-effects modelling framework provided an advantage over simpler cross-sectional approaches that use only one observation per site. By incorporating site-level sampling events in different years and including site and year as random effects, our models accounted for spatial and temporal structure in the data. This allowed us to evaluate each vegetation metric’s ability to track variation in disturbance across sites and within sites over time, which is fundamental for long-term ecological monitoring.

### Water Levels and Hydrogeomorphic Types

We also tested whether each lake-specific pIBC was suitable for use under different water-level conditions, and for wetlands of different hydrogeomorphic types. To achieve this, for each lake version of the pIBC, we fit a linear mixed-effects model (Gaussian family) with the WQLU Index as the response variable, two interaction terms (pIBC × annual water level [high or low] and pIBC × hydrogeomorphic type [lacustrine, riverine, or barrier-protected; Albert et al. 2005]) as fixed effects, and site and year as random effects. For all lakes except Lake Ontario, annual water level was classified as either high or low depending on whether the mean water level during the growing season (May to October) for a given year was above or below the median from 2011 to 2022 (water-level data were from the Great Lakes Coordinating Committee; https://www.greatlakescc.org/en/coordinating-committee-products-and-datasets). Because water levels on Lake Ontario generally experience little interannual variation as a result of outflow regulation, only the extreme water levels of 2017 and 2019 were considered high-water-level years (Smith et al. 2021). For Lake Superior, “low” annual water levels ranged from 183.19 to 183.59 m relative to the International Great Lakes Datum 1985 (IGLD) (in five of the twelve years of the study) and “high” annual water levels ranged from 183.64 to 183.85 m IGLD (seven years); for Lake Michigan and Lake Huron (which are hydrologically connected), “low” annual water levels ranged from 175.96 to 176.74 m IGLD (six years) and “high” annual water levels ranged from 176.80 to 177.38 m IGLD (six years); for Lake Erie, “low” annual water levels ranged from 174.07 to 174.51 m IGLD (six years) and “high” annual water levels ranged from 174.55 to 174.99 m IGLD (six years); and for Lake Ontario, “low” annual water levels ranged from 74.68 to 75.07 m IGLD (10 years) and “high” annual water levels ranged from 75.45 to 75.54 m IGLD (two years). For the models, non-statistically significant interactions indicated that the relationship between pIBC and the WQLU Index was similar between high- and low-water-level years, and among hydrogeomorphic wetland types. For this analysis, we also considered using raw annual water-level values and a three-category classification (high, moderate, or low), but these alternative approaches did not meaningfully change the interpretation of the results and are not presented here. All analyses were completed in R (Version 4.3.1; R Core Team 2023). Mixed models were fit using the *lme4* package in R (Bates et al. 2015), P-values were obtained using the *lmerTest* package (Kuznetsova et al. 2017), and models were compared using the *performance* package (Lüdecke et al. 2021). Statistical assumptions of linear mixed effects models were assessed using standard diagnostic plots and were adequately met in all cases.

## Results

Our 12-year dataset consisted of 41,314 quadrats (3,821 transects) sampled across the five Great Lakes (Lake Superior: 4,390 quadrats, Lake Michigan: 6,556, Lake Huron: 15,416, Lake Erie: 3,938 quadrats, Lake Ontario: 11,014). The number of unique species observed on each lake was 424 for Lake Superior (398 native, 26 non-native), 556 for Lake Michigan (483 native, 73 non-native), 714 for Lake Huron (604 native, 110 non-native), 329 for Lake Erie (278 native, 51 non-native), and 441 for Lake Ontario (361 native, 80 non-native). The most frequently observed species were *Myrica gale* on Lake Superior, *Lemna minor* on Lake Michigan, *Calamagrostis canadensis* on Lake Huron, *Ceratophyllum demersum* on Lake Erie, and *Typha × glauca* on Lake Ontario. The most abundant species (based on cumulative cover) were *Myrica gale* on Lake Superior, *Typha × glauca* on Lake Michigan and Lake Ontario, *Carex stricta* on Lake Huron, *and Phragmites australis subsp. australis* on Lake Erie.

**Fig. 2:**
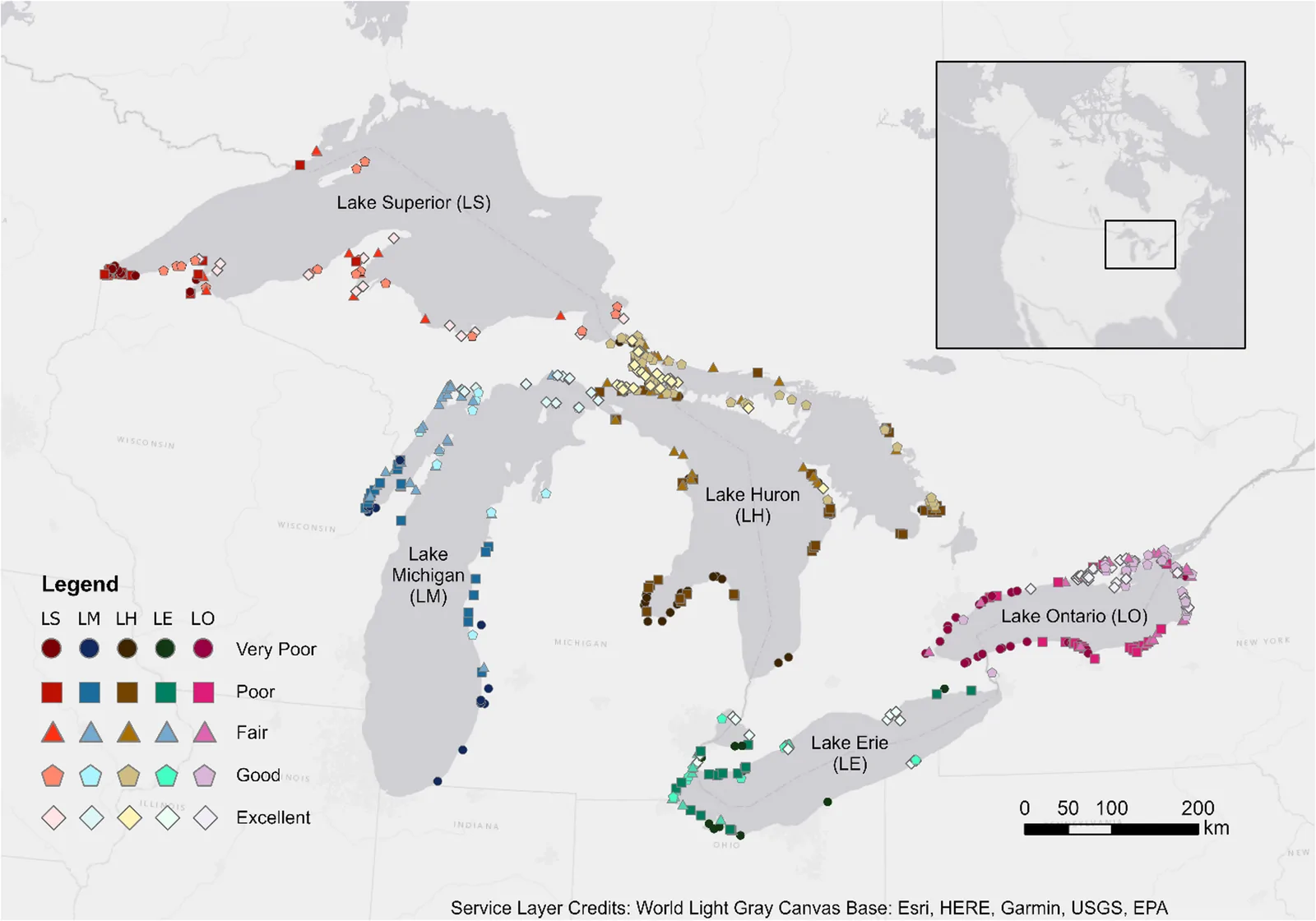
Map of plant-based Index of Biotic Condition (pIBC) values for coastal wetlands surveyed in the Laurentian Great Lakes from 2011 to 2022. For sites sampled in multiple years, values were averaged. pIBC values are lake-specific and generally should not be compared among lakes (but see Online Resource 3 for equations to standardize lake-specific pIBC values among lakes). The legend is organized as a matrix with columns for each lake and rows for condition categories (based on Table 5 below)

Lake-specific pIBC values (Fig. 2) ranged from 0.1 to 14.1 for Lake Superior (mean: 6.7), 0.5 to 24.3 for Lake Michigan (mean: 12.4), 1.5 to 21.5 for Lake Huron (mean: 12.8), 0.5 to 19.7 for Lake Erie (mean: 6.2), and 1.5 to 17.7 for Lake Ontario (mean: 10.4). Note that pIBC values are calibrated against the range of WQLU Index values for a given lake, and thus cannot generally be compared among lakes. WQLU Index values ranged from 28.5 to 100 for Lake Superior (mean: 69.6), 2.6 to 100 for Lake Michigan (mean: 57.2), 0 to 100 for Lake Huron (mean: 69.8), 0 to 72.9 for Lake Erie (mean: 25.3), and 0 to 85.9 for Lake Ontario (mean: 39.6).

### Index Validation

**Fig. 3:**
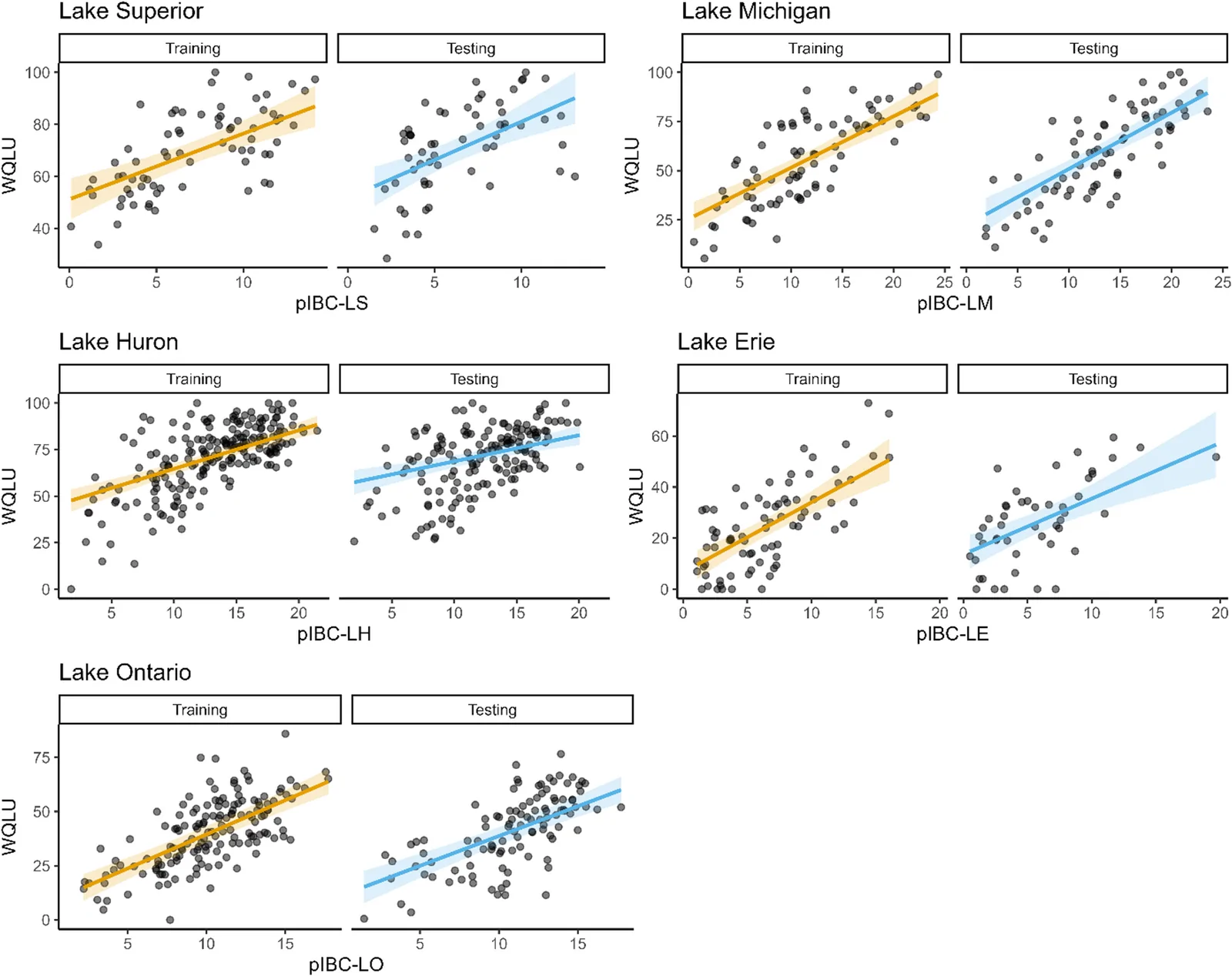
Relationships between the plant-based Index of Biotic Condition (pIBC) and Water Quality and Land Use (WQLU) Index for coastal wetlands surveyed from 2011 to 2022 for each of the Laurentian Great Lakes, shown separately for training and testing datasets. In all cases, there was a strong, statistically significant, positive association between pIBC and WQLU that was not significantly different between training and testing datasets. Points represent raw data. Lines and shaded ribbons represent model-predicted average trends and associated uncertainty (95% confidence intervals) based on a model that accounts for variation across sites and years

For all models, we found no statistically significant interaction between the lake-specific versions of the pIBC and dataset (training vs. testing) (Table 1; Fig. 3), indicating that the relationship between pIBC and WQLU was consistent across data subsets. Conversely, we found strong, statistically significant, positive associations between each lake-specific version of the pIBC and the WQLU Index (Table 1; Fig. 3), demonstrating that higher index scores corresponded with better environmental condition. Together, these results met our criteria for validation of the pIBC: consistency across datasets and a strong positive association with WQLU.

**Table 1:** Results from linear mixed-effects models used to validate each lake-specific version of the plant-based Index of Biotic Condition (pIBC)

Model	Effect	ß	SE	df	t value	*P* value
Lake Superior	pIBC_LS_	2.3	0.8	82.9	2.9	**< 0.01**
	Dataset	0.2	3	77.3	0.1	0.95
	pIBC_LS_ × Dataset	0.2	0.4	76.5	0.5	0.61
Lake Michigan	pIBC_LM_	2.5	0.5	134.2	5.3	**< 0.001**
	Dataset	−1.5	3.1	115.4	−0.5	0.63
	pIBC_LM_ × Dataset	0.1	0.2	126.7	0.5	0.59
Lake Huron	pIBC_LH_	2.4	0.4	353.5	6.2	**< 0.001**
	Dataset	5.3	2.5	345.6	2.1	**0.04**
	pIBC_LH_ × Dataset	−0.3	0.2	355.7	−1.8	0.08
Lake Erie	pIBC_LE_	3	0.7	84.8	4.6	**< 0.001**
	Dataset	3.5	2.4	78.5	1.5	0.14
	pIBC_LE_ × Dataset	−0.3	0.3	97.6	−0.9	0.37
Lake Ontario	pIBC_LO_	3.4	0.5	193.4	6.8	**< 0.001**
	Dataset	1.6	2.6	198.2	0.6	0.53
	pIBC_LO_ × Dataset	−0.2	0.2	205.7	−0.9	0.38

Each model (one per Great Lake) had WQLU Index as the response variable, pIBC, dataset (whether the site/sampling event belonged to the training or testing dataset), an interaction between both terms as fixed effects, and site and year as random effects. In all cases, there was a strong, statistically significant, positive association between pIBC and WQLU that was not significantly different between training and testing datasets, which met our validation criteria for the pIBC. Statistically significant *P*-values are indicated in boldface (*P* < 0.05)

### Comparison to Other Metrics

In comparing eight different vegetation metrics across all five Great Lakes, we found that the lake-specific pIBCs were the strongest predictors of WQLU for four of the five lakes (lowest ΔAICc and highest R^2^_m_) (Table 2). The exception was Lake Huron, where the multi-metric index of biotic integrity (IBI) (Dybiec et al. 2020) performed slightly better (∆AICc = 0, R^2^_m_ = 0.18), while the pIBC was second best (ΔAICc = 5.4, R^2^_m_ = 0.15). The IBI also performed relatively well on Lake Superior (∆AICc = 8.7, R^2^_m_ = 0.15), but no other metrics performed well on multiple lakes. All eight metrics showed statistically significant relationships with WQLU in at least three lakes (*P* < 0.05); however, with the exception of the pIBC (R^2^_m_ = 0.15–0.52), most explained little variance in WQLU (R^2^_m_ = 0–0.19).

**Table 2:** Results from linear mixed-effects models used to compare the performance of each lake-specific version of the plant-based Index of Biotic Condition (pIBC) to seven other vegetation metrics

Lake	Model/Effect	ß	SE	df	t value	*P* value	∆AICc	*R* ^2^_m_
Lake Superior	pIBC_LS_	2.4	0.4	88.8	6.5	**< 0.001**	0**	0.28**
	MeanC	4.6	1.3	143	3.5	**< 0.001**	18.3	0.08
	FQI	0.3	0.2	140.6	1.9	0.06	26.5	0.03
	wMeanC	3	0.9	136.3	3.5	**< 0.001**	19.2	0.08
	wFQI	0.3	0.1	137.9	2.5	**0.01**	24.4	0.04
	IBI	42.8	8.1	142.2	5.3	**< 0.001**	8.7*	0.15*
	Invasive Cover	−0.8	0.2	140.9	−4.9	**< 0.001**	10.6	0.14
	Native Richness	0	0.1	133.8	0.5	0.64	29.6	0
Lake Michigan	pIBC_LM_	2.5	0.2	140	11.5	**< 0.001**	0**	0.52**
	MeanC	4.5	1.4	156.4	3.2	**< 0.01**	49.3	0.03
	FQI	0.5	0.1	157.8	3.8	**< 0.001**	46.3*	0.04*
	wMeanC	2.3	0.8	147.4	2.9	**< 0.01**	50.3	0.02
	wFQI	0.4	0.1	155.1	3.5	**< 0.001**	47.6	0.04*
	IBI	18.1	6.8	126.5	2.7	**< 0.01**	51.7	0.02
	Invasive Cover	−0.1	0.1	120	−1.4	0.16	56	0
	Native Richness	0.1	0.1	130	2.1	**0.03**	53.9	0.01
Lake Huron	pIBC_LH_	1.5	0.2	442	8.2	**< 0.001**	5.4*	0.15*
	MeanC	4.1	0.9	449.1	4.6	**< 0.001**	33	0.05
	FQI	0.4	0.1	420.4	5	**< 0.001**	30	0.04
	wMeanC	3.6	0.5	448.3	7.7	**< 0.001**	5.8	0.12
	wFQI	0.5	0.1	446.9	7.5	**< 0.001**	8.9	0.12
	IBI	44.3	4.8	421.4	9.2	**< 0.001**	0**	0.18**
	Invasive Cover	−0.3	0.1	400.1	−4.3	**< 0.001**	34.4	0.03
	Native Richness	0.1	0	304.9	2.4	**0.02**	43.7	0.01
Lake Erie	pIBC_LE_	2.4	0.3	98.4	8.2	**< 0.001**	0**	0.37**
	MeanC	4.6	1.7	143	2.6	**< 0.01**	31.5*	0.04*
	FQI	0.5	0.2	131.4	2.6	**< 0.01**	31.7	0.03
	wMeanC	1.1	0.8	122	1.4	0.17	35.2	0.01
	wFQI	0.2	0.2	117.9	1.4	0.18	35.3	0.01
	IBI	25.7	10.7	125	2.4	**0.02**	31.8	0.03
	Invasive Cover	25.7	10.7	125	2.4	**0.02**	32.8	0.02
	Native Richness	0.2	0.1	112.6	1.6	0.1	34.6	0.01
Lake Ontario	pIBC_LO_	2.8	0.2	214.1	13.1	**< 0.001**	0**	0.38**
	MeanC	9.2	1.5	305.1	6.2	**< 0.001**	82	0.09
	FQI	1.1	0.1	289.7	8.7	**< 0.001**	57.5*	0.19*
	wMeanC	3.7	1	279.5	3.7	**< 0.001**	95.8	0.03
	wFQI	0.8	0.1	302.1	6.5	**< 0.001**	73.1	0.1
	IBI	32.7	8.4	278.2	3.9	**< 0.001**	95.1	0.03
	Invasive Cover	−0.1	0.1	269.5	−1.1	0.26	107.5	0
	Native Richness	0.3	0.1	303.7	5.6	**< 0.001**	82.2	0.08

Each row of the table contains the results from a model (one per metric per Great Lake) with the Water Quality and Land Use (WQLU) Index as the response variable, one of the eight vegetation metrics as a fixed effect, and site and year as random effects. The vegetation metrics compared to the lake-specific versions of the pIBC were mean coefficient of conservatism (meanC), floristic quality index (FQI), cover-weighted mean C (wMeanC), cover-weighted FQI (wFQI), a recently developed multi-metric index of biotic integrity (IBI), invasive species cover, and native species richness. The lake-specific pIBCs were the strongest predictors of WQLU for four of the five lakes (lowest ΔAICc and highest marginal R^2^ [R²_m_]). Statistically significant *P*-values are indicated in boldface (*P* < 0.05). The lowest ∆AICc and highest R^2^_m_ values are indicated with a double asterisk, and the second-best values are indicated with a single asterisk

### Water Levels and Hydrogeomorphic Types

**Fig. 4:**
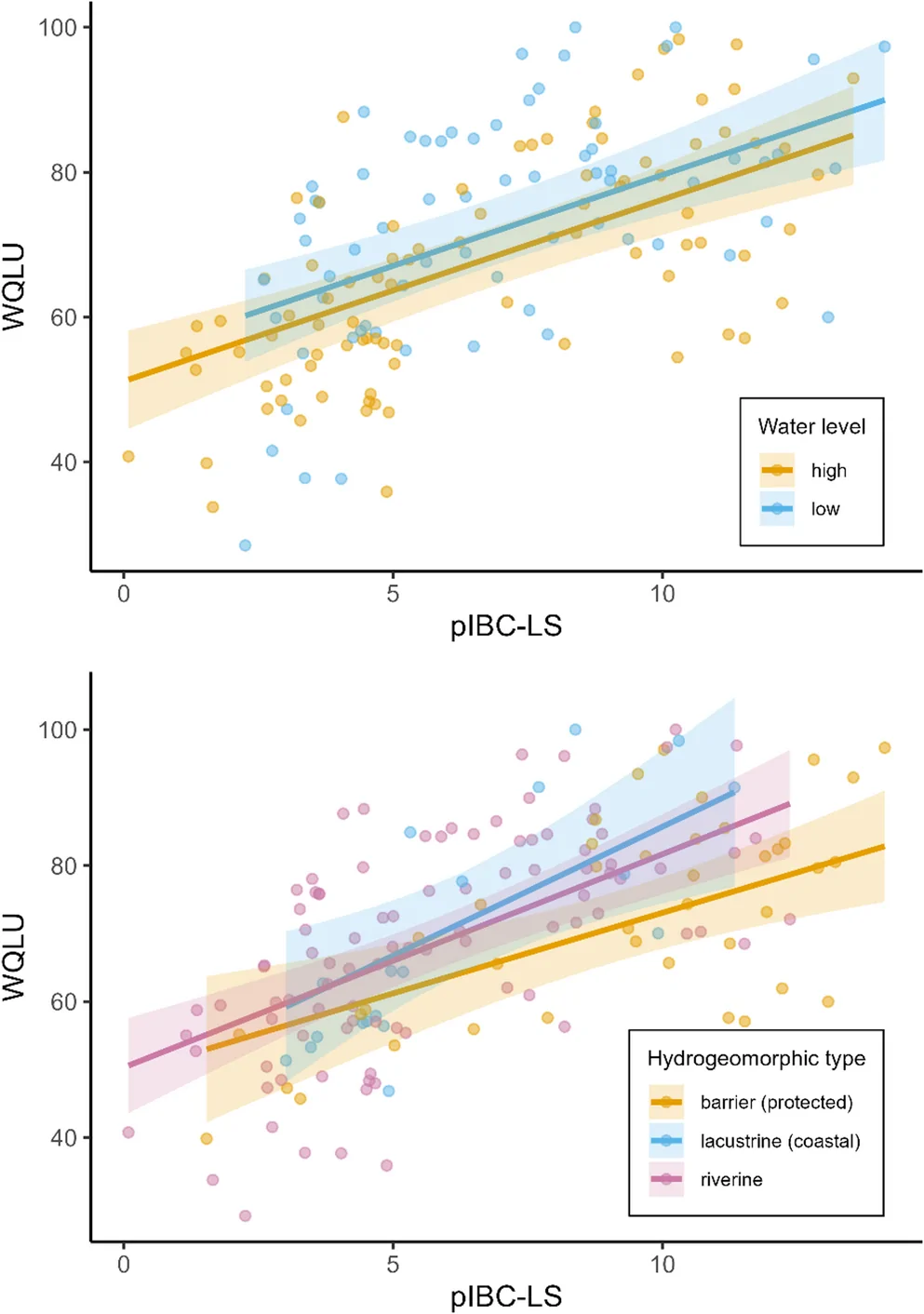
Relationships between the Lake Superior version of the plant-based Index of Biotic Condition (pIBC_LS_) and the Water Quality and Land Use (WQLU) Index, shown in high- versus low-water-level years (top), and for wetland hydrogeomorphic types (bottom). The association between pIBC_LS_ and WQLU did not differ in high- versus low-water-level years, or among lacustrine, riverine, and barrier-protected hydrogeomorphic types (pIBC_LS_ × annual water level and pIBC_LS_ × hydrogeomorphic type interaction terms were not significantly significant). Points represent raw data. Lines and shaded ribbons represent model-predicted average trends and associated uncertainty (95% confidence intervals) based on a model that accounts for variation across sites and years. Plots for the other four Great Lakes can be found in Online Resource 2

We found no statistically significant interactions between any of the lake-specific versions of the pIBC and annual water levels or wetland hydrogeomorphic type (Table 3), indicating that the associations between different versions of the pIBC and WQLU were not significantly different between high- and low-water-level years, or among lacustrine, riverine, and barrier-protected hydrogeomorphic types (Fig. 4, Online Resource 2).

**Table 3:** Results from linear mixed-effects models used to test whether each lake-specific version of the plant-based Index of Biotic Condition (pIBC) was suitable for use under different water-level conditions, and for wetlands of different hydrogeomorphic types

Model	Effect	Sum Sq	Mean Sq	Num df	Den df	F value	*P* value
Lake Superior	pIBC_LS_	2875.2	2875.2	1	102.6	40.3	**< 0.001**
	Water Level	28.6	28.6	1	56.4	0.4	0.53
	HGM	7.5	3.7	2	89.7	0.1	0.95
	pIBC_LS_ × Water Level	5.7	5.7	1	108.4	0.1	0.78
	pIBC_LS_ × HGM	113.4	56.7	2	94.5	0.8	0.45
Lake Michigan	pIBC_LM_	2071.9	2071.9	1	161.5	38.6	**< 0.001**
	Water Level	0.1	0.1	1	55.9	0	0.96
	HGM	0	0	2	145.2	0	1
	pIBC_LM_ × Water Level	10.7	10.7	1	92.9	0.2	0.66
	pIBC_LM_ × HGM	42.4	21.2	2	169.9	0.4	0.67
Lake Huron	pIBC_LH_	1665.7	1665.7	1	410.9	22.7	**< 0.001**
	Water Level	0	0	1	81.8	0	1
	HGM	98.9	49.5	2	390.7	0.7	0.51
	pIBC_LH_ × Water Level	0	0	1	259	0	0.98
	pIBC_LH_ × HGM	24.2	12.1	2	420.2	0.2	0.85
Lake Erie	pIBC_LE_	1713.1	1713.1	1	140.2	21.9	**< 0.001**
	Water Level	181.6	181.6	1	34	2.3	0.14
	HGM	78	39	2	99	0.5	0.61
	pIBC_LE_ × Water Level	73.2	73.2	1	95.6	0.9	0.34
	pIBC_LE_ × HGM	94.9	47.4	2	108	0.6	0.55
Lake Ontario	pIBC_LO_	3790.6	3790.6	1	284.2	56.2	**< 0.001**
	Water Level	20.8	20.8	1	44.5	0.3	0.58
	HGM	89.4	44.7	2	234.4	0.7	0.52
	pIBC_LO_ × Water Level	26.4	26.4	1	225.6	0.4	0.53
	pIBC_LO_ × HGM	23	11.5	2	248.7	0.2	0.84

Each model (one per Great Lake) had the Water Quality and Land Use (WQLU) Index as the response variable, two interaction terms (pIBC × annual water level [high or low] and pIBC × hydrogeomorphic type [HGM; lacustrine, riverine, or barrier-protected]) as fixed effects, and site and year as random effects. In all cases, there was a strong, statistically significant, positive association between pIBC and WQLU that was not significantly different between high- and low-water-level years, or among lacustrine, riverine, and barrier-protected hydrogeomorphic types. Given the inclusion of three-level categorical predictors in these models, we have reported results from Type III ANOVA tables to summarize the overall significance of fixed effects. Statistically significant *P*-values are indicated in boldface (*P* < 0.05)

## Discussion

Plant communities are widely used as indicators of anthropogenic disturbance (e.g., Bourdaghs et al. 2006; Grabas et al. 2012; Dybiec et al. 2020). This likely reflects the simplicity and cost-effectiveness of plant surveys compared to other taxa and, more importantly, that in general, the presence of rooted, immobile plants directly reflects their ability to tolerate local environmental conditions. In this study, we used a massive dataset (41,314 quadrats surveyed in 563 wetlands over 12 years) to develop and assess the performance of a plant-based Index of Biotic Condition (pIBC) for coastal wetlands for each of the Laurentian Great Lakes. We found that each lake-specific version of the pIBC was a consistently strong predictor of anthropogenic disturbance (based on water quality and surrounding land use), including at independent testing sites that were excluded from index calibration. Furthermore, comparisons with seven other plant-based metrics demonstrate that the pIBC provides improved within-lake sensitivity to water-quality and land-use disturbance, offering finer resolution for interpreting spatial and temporal variation at the scale where most management decisions are made. Moreover, because species weights have now been established, the index can be calculated easily (see equation above and https://doi.org/10.5061/dryad.0cfxpnwg2 for weights for all species for each lake and a pIBC calculator). This, combined with its straightforward interpretation, makes the pIBC a valuable tool for conservation practitioners seeking rapid assessment of coastal wetland condition.

The pIBC is computed by aggregating species “weights,” which are calculated based on two parameters: the anthropogenic disturbance value when the predicted probability of species occurrence is maximized and the difference between the maximum probability of occurrence and the probability of occurrence when disturbance is greatest (Howe et al. 2023). The first parameter generally reflects the sensitivity of the species to environmental condition, whereas the second parameter reflects the responsiveness of the species to change in the environmental condition gradient. Species with high weights are, therefore, reliable “indicator species” because they tend to be present in higher quality sites and less so in lower quality sites (i.e., highly sensitive and highly responsive). It is the second parameter that makes the pIBC conceptually different than traditional FQA metrics, which are essentially variations of an average tolerance to disturbance and fidelity to specific habitats (based on professional judgment), but do not integrate likelihood of occurrence. A key distinction is that, in many cases, uncommon species have high Coefficient of Conservatism values but low weights relative to more common species. Low weights for uncommon species may seem counterintuitive because this implies that a site with many uncommon species could score lower than a site with a few medium- to high-weight species; however, this approach is justifiable for several reasons. First, extremely rare species (defined as those that occurred less than five times in our training dataset; see Methods for details) are entirely omitted from pIBC calculations, so it is only a subset of quasi-rare (uncommon) species that influence pIBC scores. This improves the reliability of biotic response functions and derived weights by avoiding misleading conclusions due to overfitting or outlier effects. Second, because uncommon species are inherently detected less frequently in quadrat-based surveys than common species, they have a relatively small impact on pIBC scores. Common species are observed more consistently, so their presence drives site-level scores and ensures that results reflect dominant ecological signals rather than being skewed by isolated or incidental occurrences. Third, rarity is often driven by factors beyond anthropogenic disturbance (i.e., range limit, availability of suitable habitat, habitat specificity [e.g., soil type, hydrology, microclimate], life history traits [e.g., growth rate, dispersal capability]; Flather and Sieg 2007), making rare species less reliable indicators of site condition.

We also demonstrated that the pIBC was effective under different water-level conditions and for wetlands of different hydrogeomorphic types, which is critical for a biological indicator for Great Lakes coastal wetlands, given that these variables can influence plant community composition independent of anthropogenic disturbance (Wilcox et al. 2002; Albert et al. 2005). Wilcox et al. (2002) argued that water-level variation exerts such a strong influence that biotic metrics for Great Lakes coastal wetlands must account for different water-level histories. A method that has been used to overcome this constraint is to calibrate indicators for distinct plant zones whose presence and distribution are closely tied to water-level variation (Burton et al. 1999; Uzarski et al. 2004, 2005; Cooper et al. 2018; Dybiec et al. 2020). Although we did not take this approach, we achieved a similar outcome by explicitly linking the presence of plant species to disturbance using a dataset that encompasses a period of high and low water levels in the Great Lakes. Therefore, species weights reflect relationships with disturbance across varying hydrological conditions. In addition, although certain species do, of course, occur more or less frequently under different hydrological conditions (e.g., *Eleocharis palustris* occurred in 10% of quadrats during low water levels and 5% of quadrats during high water levels on Lake Huron), the large number of species used in the different versions of the pIBC (59–251) (most of which did not show notable differences in occurrence in high versus low water years) ensures that no single species disproportionately influences site-level pIBC values. We note that our sampling methodology did not allow us to explicitly test the performance of the pIBC in the context of lagged vegetation responses to changing water levels (e.g., plant community shifts when high waters are followed by low waters; Wilcox and Meeker 1991; Wilcox and Nichols 2008). However, in years where water levels during the growing season were notably higher or lower than the previous year (arbitrarily defined as a change of > 0.2 m IGLD), lake-specific correlations between the pIBC and the WQLU Index were generally moderate to strong (*r* = 0.36–0.88 for all comparisons except one [Lake Superior in 2021: *r* = 0.17]; median *r* = 0.74). These correlations are not a formal test of lagged responses at individual sites (because the same sites were infrequently sampled in consecutive years), and they are only representative of water-level dynamics that occurred during the study period. Nevertheless, the moderate-to-strong correlations observed suggest that lag effects generally did not influence the ability of the pIBC to track disturbance gradients at the regional scale.

In general, we were surprised by the limited variation in the WQLU Index that was explained by many of the metrics that we compared to the pIBC; none had R^2^_m_ values greater than 10% for Lake Michigan and Lake Erie, three were greater than 10% for Lake Huron, and two were greater than 10% for Lake Superior and Lake Ontario. These low R²_m_ values indicate that most metrics capture relatively little of the within-site temporal variation in condition after differences among sites and years are accounted for. These results were especially striking when contrasted with adjusted R^2^ values from models that did not include random effects for site and year. For example, in analyses not presented here in detail, we found that selecting a random year for each site and fitting a simple linear model between FQI and WQLU for Lake Michigan substantially increased the variance explained by FQI to 27%. However, this higher R² largely reflects between-site differences and therefore overestimates the ability of FQI to detect changes in condition over time at individual sites. In the mixed-effects framework, the marginal R² for FQI in Lake Michigan was only 0.04, indicating that it provides little information about temporal change despite appearing effective for cross-sectional comparisons. Similar patterns were observed for many of the other metrics that we tested (Table 4). In contrast, the pIBC demonstrated consistently strong performance in both modelling frameworks – it explained meaningful between-site differences while still capturing within-site temporal changes, suggesting that it is effective both for differentiating wetland condition among sites and for tracking site-level changes through time.

**Table 4:** Results from simple linear models used to compare the performance of each lake-specific version of the plant-based Index of Biotic Condition (pIBC) to seven other vegetation metrics

Lake	Model/Effect	ß	SE	t value	*P* value	∆AICc	*R* ^2^_adj_
Lake Superior	pIBC_LS_	3.5	0.5	6.7	**< 0.001**	0**	0.38**
	MeanC	5.4	1.9	2.8	**< 0.01**	27.9	0.09
	FQI	0.6	0.3	2.4	**0.02**	30.2	0.06
	wMeanC	5	1.2	4	**< 0.001**	20.6	0.17
	wFQI	0.7	0.2	3.4	**< 0.01**	24.7	0.13
	IBI	72.4	12.3	5.9	**< 0.001**	6.9	0.32
	Invasive Cover	−1.4	0.2	−6.3	**< 0.001**	3.1*	0.35*
	Native Richness	0.3	0.2	1.8	0.08	32.6	0.03
Lake Michigan	pIBC_LM_	3.3	0.3	12.4	**< 0.001**	0**	0.64**
	MeanC	17.6	2.4	7.3	**< 0.001**	47*	0.38*
	FQI	1.8	0.2	7.1	**< 0.001**	49.2	0.36
	wMeanC	10	1.6	6.1	**< 0.001**	57.6	0.3
	wFQI	1.4	0.2	6.8	**< 0.001**	51.2	0.35
	IBI	88.7	14	6.3	**< 0.001**	55.6	0.32
	Invasive Cover	−1	0.2	−4.5	**< 0.001**	70.7	0.18
	Native Richness	0.6	0.1	4.6	**< 0.001**	70.1	0.19
Lake Huron	pIBC_LH_	2.6	0.3	9.8	**< 0.001**	12*	0.33*
	MeanC	9.6	1.3	7.4	**< 0.001**	42.9	0.22
	FQI	1.1	0.1	7.9	**< 0.001**	36.3	0.24
	wMeanC	6.9	0.7	9.7	**< 0.001**	13.5	0.33*
	wFQI	0.9	0.1	9.7	**< 0.001**	14.4	0.32
	IBI	75.9	7.1	10.7	**< 0.001**	0**	0.37**
	Invasive Cover	−1	0.1	−7.5	**< 0.001**	41.8	0.22
	Native Richness	0.3	0.1	4.5	**< 0.001**	72	0.09
Lake Erie	pIBC_LE_	2.7	0.4	7.1	**< 0.001**	0**	0.43**
	MeanC	11.4	2.6	4.3	**< 0.001**	21	0.21
	FQI	1.3	0.3	4.1	**< 0.001**	22.8	0.19
	wMeanC	3.1	1.5	2	**0.05**	33.9	0.04
	wFQI	0.9	0.3	3	**< 0.01**	29.4	0.11
	IBI	88.1	17.7	5	**< 0.001**	16.3*	0.27*
	Invasive Cover	−0.3	0.1	−2.6	**0.01**	31.2	0.08
	Native Richness	0.5	0.2	2.7	**< 0.01**	30.7	0.09
Lake Ontario	pIBC_LO_	3.2	0.3	11.4	**< 0.001**	0**	0.48**
	MeanC	17.7	1.9	9.2	**< 0.001**	25.9	0.37
	FQI	1.6	0.2	9.9	**< 0.001**	18.6*	0.4*
	wMeanC	7.4	1.6	4.5	**< 0.001**	74.2	0.12
	wFQI	1.3	0.2	7.4	**< 0.001**	46.8	0.27
	IBI	87.3	13.3	6.6	**< 0.001**	55.4	0.23
	Invasive Cover	−0.1	0.1	−0.8	0.41	92.9	0
	Native Richness	0.5	0.1	6.4	**< 0.001**	57.2	0.22

Each row of the table contains the results from a model (one per metric per Great Lake) with the Water Quality and Land Use (WQLU) Index as the response variable and one of the eight vegetation metrics as the predictor. The vegetation metrics compared to the lake-specific versions of the pIBC were mean coefficient of conservatism (meanC), floristic quality index (FQI), cover-weighted mean C (wMeanC), cover-weighted FQI (wFQI), a recently developed multi-metric index of biotic integrity (IBI), invasive species cover, and native species richness. The lake-specific pIBCs were the strongest predictors of WQLU for four of the five lakes (lowest ΔAICc and highest adjusted R^2^ [R²_adj_]). Statistically significant *P*-values are indicated in boldface (*P* < 0.05). The lowest ∆AICc and highest R^2^_adj_ values are indicated with a double asterisk, and the second-best values are indicated with a single asterisk

Our analyses provides insight into the strengths and limitations of FQA metrics as indices of disturbance in Great Lakes coastal wetlands. Most FQA metrics showed statistically significant positive associations with WQLU, but few explained meaningful variance (low R^2^_m_). None performed well in lakes Superior, Michigan, or Erie, only the two cover-weighted metrics did for Lake Huron, and only FQI did for Lake Ontario. The observed relationships between the FQA metrics tested and disturbance were generally consistent with other studies. For example, in a study describing the development of an index of biotic integrity, Grabas et al. (2012) found statistically significant relationships between both mean C and FQI and disturbance (Water Quality Index; Chow-Fraser 2006) based on submerged aquatic vegetation in coastal wetlands on Lake Ontario. Similarly, Dybiec et al. (2020) found significant relationships between mean C and disturbance (as measured by WQLU and AgDev [Host et al. 2019]) in submerged aquatic (with WQLU), emergent (with WQLU), and wet meadow (with AgDev) vegetation zones in coastal wetlands of Lake Huron. Bourdaghs et al. (2006) assessed the performance of multiple FQA metrics in coastal wetlands of northwestern Lake Huron, northern Lake Michigan, and southern Lake Superior and found similar statistically significant relationships with disturbance, although they did not analyze each lake individually. They also found little difference in metrics that were weighted or not weighted by abundance, which is generally consistent with our results (with the exception of Lake Huron) and others (briefly reviewed in Spyreas 2019). However, Bourdaghs et al. (2006) concluded that FQA metrics were acceptable ecological indicators of condition, whereas our results suggest more limited utility (with a few exceptions: the two cover-weighted metrics for Lake Huron and FQI for Lake Ontario). While many of these metrics seem to reflect broader, among-site differences in water quality and land use (suggesting that they are useful for differentiating or ranking sites by disturbance as a snapshot in time), their low marginal R² values in mixed models suggest limited responsiveness to site-level changes over time. An important caveat is that this conclusion is based on single lakes, whereas the Coefficients of Conservatism (C) values used for these analyses were based on larger regions (Michigan and southern Ontario). It is therefore possible that these FQA metrics would have performed better on larger scales, but that was beyond the scope of this study.

One possible advantage of FQA metrics is that all native species are assigned Coefficients of Conservatism. In contrast, although we generated weights for many species, there were cases where species observed during a site survey lacked weights. At the site level, an average of 83% of observed species (range: 33–100%) had assigned weights. We evaluated whether this could bias pIBC performance by fitting lake-specific linear mixed-effects models with the WQLU index as the response, pIBC, the proportion of species lacking weights, and their interaction as fixed effects, and site and year as random effects. Across all lakes, this interaction was not significant (*P* > 0.14), suggesting that incomplete species weight coverage is unlikely to affect pIBC-based assessment of wetland condition.

With respect to the other metrics compared, the IBI developed by Dybiec et al. (2020) was calibrated using lacustrine wetlands of Lake Huron, so it is unsurprising that it performed best in Lake Huron (∆AICc = 0, R^2^_m_ = 0.18). The pIBC performed adequately in Lake Huron (∆AICc = 5.4, R^2^_m_ = 0.15) but notably worse than the other lakes (∆AICc = 0, R^2^_m_ = 0.28–0.52). We suspect that none of the metrics performed exceptionally well for Lake Huron because many Lake Huron coastal wetlands share a narrow range of WQLU Index values (64% of sites had WQLU values between 60 and 90). This narrow range likely results in limited disturbance-related differentiation among plant communities that can be “picked up” by simple numerical indicators. Somewhat counterintuitively, invasive species cover was a good predictor of water quality and land-use disturbance in Lake Superior but did not perform well in any other lakes, all of which are more widely affected by invasive species. This finding likely reflects the fact that there are few wetlands, especially in the more southern parts of the Great Lakes, that are not extensively colonized by invasive species.

Conservation practitioners need to be able to put wetland condition (pIBC) values into context. Consequently, categorical descriptors of site condition (e.g., Good, Fair, Poor) are commonly used in monitoring projects and programs. For this reason, we have provided two options for categorical descriptors (three- and five-categories) that can be used depending on the application (Table 5). These categories are based on a percentile range of observed pIBC values from our full dataset for each lake. For the five-category framework, a pIBC score above the 80th percentile is considered to be in Excellent condition; between the 60th and 80th percentiles is considered Good condition; between the 40th and 60th percentiles is considered Fair condition; between the 20th and 40th percentiles is considered Poor condition; and below the 20th percentile is considered Very Poor condition. For the three-category framework, a pIBC score above the 67th percentile is considered to be in Good condition; between the 33rd and 67th percentile is considered Fair condition; and below the 33rd percentile is considered Poor condition. This framework is useful because the categories are unambiguous and meaningful; for instance, an Excellent score indicates that a wetland of interest is comparable to the top 20% of coastal wetlands for a given lake. It is, however, important to note that the use of categorical descriptors to describe site condition can occasionally be misleading. For example, two sites that score in the 79th and 81 st percentile using the five-category framework would receive different categorical scores (Good and Excellent, respectively), despite very similar indicator values, whereas two sites that score in the 60th and 79th percentile would receive the same categorical score (Good), despite a much larger difference in indicator values. While the categorical scores are useful for painting a general picture, more attention should be given to the raw indicator values.

**Table 5:** Percentile-based categorical descriptors of the plant-based Index of Biotic Condition (pIBC) for each of the Laurentian Great Lakes

			pIBC Range				
	Percentile	Descriptor	L. Superior	L. Michigan	L. Huron	L. Erie	L. Ontario
	80^th^ to100^th^	Excellent	> 10.1	> 18.4	> 16.7	> 9.0	> 13.2
	60^th^ to 80^th^	Good	7.7 to 10.1	15.2 to 18.4	14.7 to 16.7	7.3 to 9.0	11.5 to 13.2
5-cat.	40^th^ to 60^th^	Fair	5.0 to 7.6	10.8 to 15.1	12.0 to 14.6	4.7 to 7.2	10.0 to 11.4
	20^th^ to 40^th^	Poor	3.6 to 4.9	6.8 to 10.7	8.9 to 11.9	2.5 to 4.6	7.7 to 9.9
	<20^th^	Very Poor	< 3.6	< 6.8	< 8.9	< 2.5	< 7.7
	67^th^ to 100^th^	Good	> 8.6	> 15.1	> 15.1	> 7.6	> 12.0
3-cat.	33^rd^ to 67^th^	Fair	4.5 to 8.6	9.7 to 15.1	11.0 to 15.1	3.7 to 7.6	9.4 to 12.0
	<33^rd^	Poor	< 4.5	< 9.7	< 11.0	< 3.7	< 9.4

Shown are two frameworks (three- and five-categories) for describing wetland condition. For each lake, pIBC score ranges correspond to percentile thresholds derived from the full dataset of observed site values. pIBC values are lake-specific and generally should not be compared among lakes (but see Online Resource 3 for equations to standardize values among lakes)

One important feature of the pIBC is that the different versions were calibrated against the range of WQLU Index values for a given lake (where the greatest values of pIBC are associated with the greatest values of the WQLU Index for each lake). Calibrating pIBCs to be lake-specific is advantageous because it maximizes within-level resolution of the index, which is the scale that is often most relevant for conservation practitioners and land managers, but developing the index in this way means pIBC values cannot be directly compared among lakes because different lakes have different ranges of WQLU Index values (e.g., the highest WQLU Index value on Lake Erie was 72.9 whereas the highest on Lake Huron was 100). There will, however, be instances where site comparisons among lakes may be required. To allow for comparison of pIBC values among lakes, we developed equations to standardize lake-specific pIBC values to the range of WQLU Index values for a given lake (see Online Resource 3). However, users should interpret standardized pIBC values across lakes with caution, as these values reflect relative rankings within the original context of each lake’s disturbance gradient (i.e., standardization aligns the numerical range of pIBC values across lakes but does not fully account for underlying differences in lake-specific drivers of disturbance and associated plant community response). Therefore, while standardized pIBC scores can support broad comparisons or regional summaries, the pIBC was developed primarily for detailed assessments of wetland condition within lakes.

## Conclusion

The plant-based Index of Biotic Condition (pIBC) is a reliable tool for assessing the condition of Great Lakes coastal wetlands. The pIBC captures both between-site differences and within-site temporal changes, providing high resolution for detecting anthropogenic disturbance. Calibrating pIBC values to each lake ensures that the index reflects local variation in wetland condition, which is the scale most relevant for management decisions. It is also robust to high and low water levels and applicable across wetland hydrogeomorphic types. For these reasons, the pIBC represents a valuable metric for supporting conservation and monitoring efforts in the region.

While other plant-based metrics, such as FQA-derived indices, may capture different aspects of wetland ecology (e.g., species exclusivity to remnant habitats), the pIBC provides a precise and interpretable measure of anthropogenic disturbance at the lake scale. It is imperative, however, that conservation practitioners clearly define project goals and use tools that most appropriately serve those specific goals. Calculation of pIBCs or any other metric should only be a starting point for more in-depth site assessment. Disagreement among metrics may provide insight into prominent factors influencing the ecology of a site, including different elements of disturbance (e.g., presence of nuisance fauna), historical land use, or site-specific characteristics. By considering multiple metrics and critically interpreting the results, conservation practitioners can make informed decisions that balance restoration or conservation priorities in these ecologically, economically, and culturally important ecosystems.

## Supplementary Information

Online Resources 1–3 can be found online in the Supplementary Information section.

Supplementary Material 1

Supplementary Material 2

Supplementary Material 3

Supplementary Material 4

Supplementary Material 5

Supplementary Material 6

Supplementary Material 7

## Data Availability

Raw plant survey data and Water Quality and Land Use Index data are available upon request from the Great Lakes Coastal Wetlands Monitoring Program (https://www.greatlakeswetlands.org/Account/Request.vbhtml). The dataset generated and analyzed for this study is available in the Dryad Digital Repository at https://doi.org/10.5061/dryad.0cfxpnwg2.
