# Supplementary material for: Beyond Floristic Quality: A Data-Driven Approach to Assessing Coastal Wetland Condition Using Plants
Source: Wetlands (Wilmington). 2026 Oct 6;46(7):129. doi: 10.1007/s13157-026-02094-z (PMC13642451; doi:10.1007/s13157-026-02094-z)
Supplement: Supplementary file 1 [file 13157_2026_2094_MOESM1_ESM.pdf]

Wetlands

Online Resource 1: Lake Erie biotic response curves

Beyond floristic quality: A data-driven approach to assessing coastal wetland condition using plants

Giuseppe E. Fiorino[1]*, Simon T. Denomme-Brown[1], Hayley N. Rogers[1], Ian M. Smith[1], Kathryn L. Amatangelo[2], Matthew J. Cooper[3], Nicholas P. Danz[4], Robert W. Howe[5], Rachel E. Schultz[2], Bridget A. Wheelock[6], Douglas A. Wilcox[2], Greg P. Grabas[1]

[1]Environment and Climate Change Canada, 4905 Dufferin Street, Toronto, Ontario M3H 5T4, Canada

[2]Department of Environmental Science and Ecology, SUNY-Brockport, 350 New Campus Drive, Brockport, NY 14420, USA

[3]Department of Biology, Grand Valley State University, Allendale, MI 49401, USA

[4]Department of Natural Sciences, University of Wisconsin-Superior, 1605 Catlin Avenue, Superior, WI 54880, USA

[5]Cofrin Center for Biodiversity, University of Wisconsin-Green Bay, Green Bay, WI 54311-7001, USA

[6]Institute for Great Lakes Research and Department of Biology, Central Michigan University, Mount Pleasant, MI, USA

*Corresponding Author (email: giuseppe.fiorino@ec.gc.ca, ORCID: 0000-0002-1569-0767)

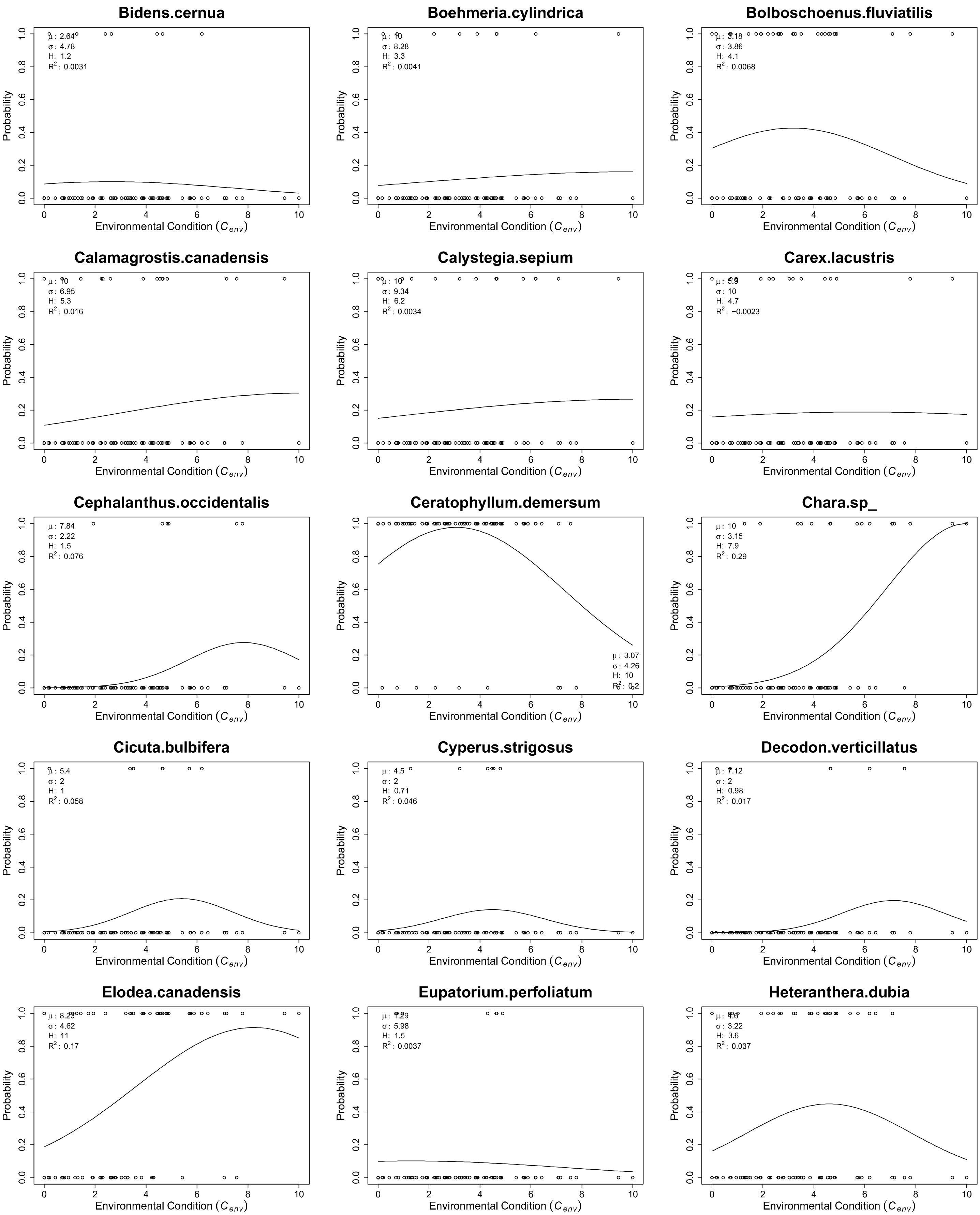

Bidens.cernua
μ: 2.64
σ: 4.78
H: 1.2
R²: 0.0031
Boehmeria.cylindrica
μ: 10
σ: 8.28
H: 3.3
R²: 0.0041
Bolboschoenus.fluviatilis
μ: 3.18
σ: 3.86
H: 4.1
R²: 0.0068
Calamagrostis.canadensis
μ: 10
σ: 6.95
H: 5.3
R²: 0.016
Calystegia.sepium
μ: 10
σ: 9.34
H: 6.2
R²: 0.0034
Carex.lacustris
σ: 10
H: 4.7
R²: −0.0023
Cephalanthus.occidentalis
μ: 7.84
σ: 2.22
H: 1.5
R²: 0.076
Ceratophyllum.demersum
μ: 3.07
σ: 4.26
H: 10
Chara.sp_
μ: 10
σ: 3.15
H: 7.9
R²: 0.29
Cicuta.bulbifera
μ: 5.4
σ: 2
H: 1
R²: 0.058
Cyperus.strigosus
μ: 4.5
σ: 2
H: 0.71
R²: 0.046
Decodon.verticillatus
μ: 7.12
σ: 2
H: 0.98
R²: 0.017
Elodea.canadensis
μ: 8.23
σ: 4.62
H: 11
R²: 0.17
Eupatorium.perfoliatum
μ: 1.29
σ: 5.98
H: 1.5
R²: 0.0037
Heteranthera.dubia
σ: 3.22
H: 3.6
R²: 0.037
Probability
Environmental Condition (C_{env})

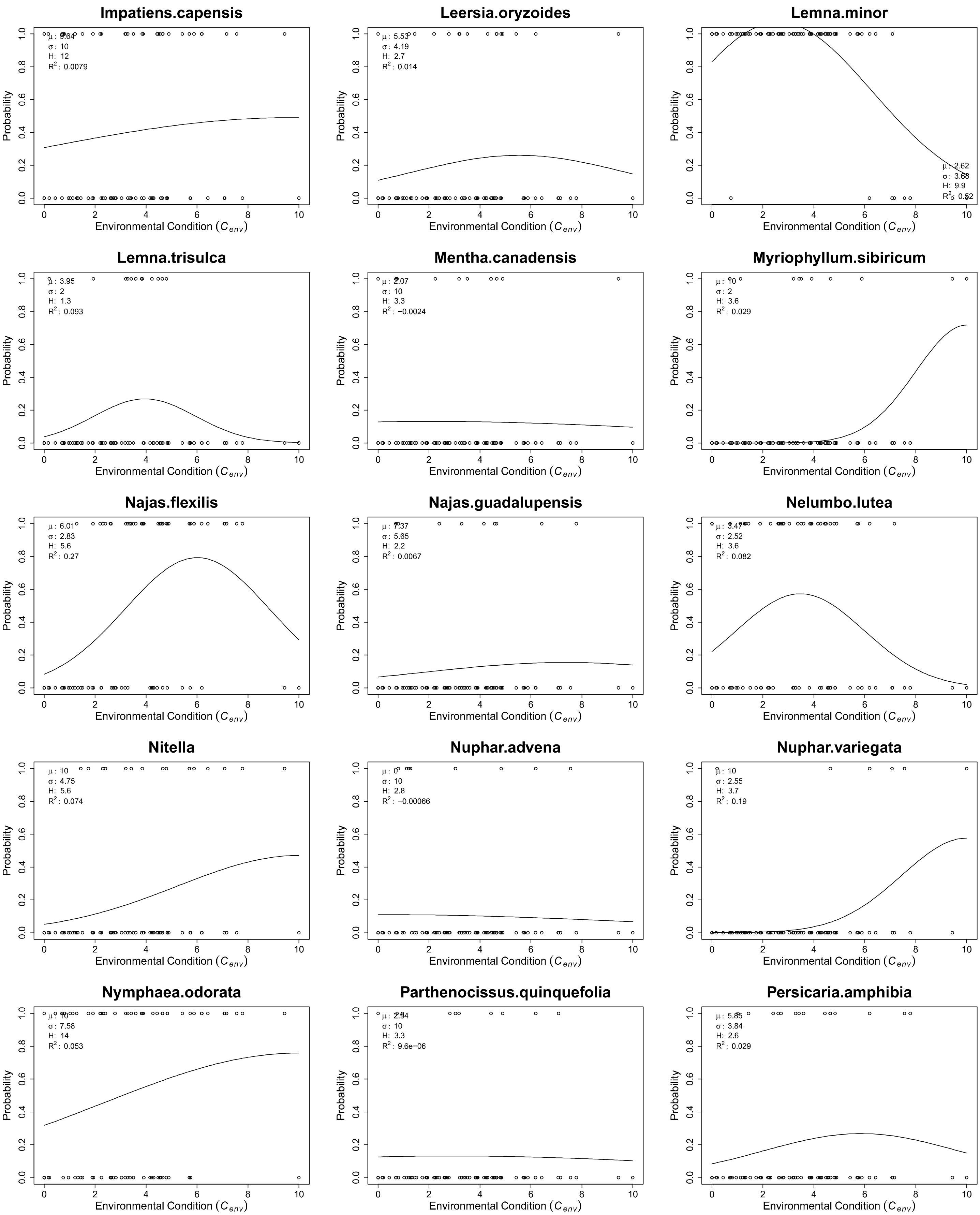
Impatiens.capensis
μ: 5.64
σ: 10
H: 12
R²: 0.0079
Leersia.oryzoides
μ: 5.53
σ: 4.19
H: 2.7
R²: 0.014
Lemna.minor
μ: 2.62
σ: 3.68
H: 9.9
R²: 0.52
Lemna.trisulca
μ: 3.95
σ: 2
H: 1.3
R²: 0.093
Mentha.canadensis
μ: 2.07
σ: 10
H: 3.3
R²: −0.0024
Myriophyllum.sibiricum
μ: 10
σ: 2
H: 3.6
R²: 0.029
Najas.flexilis
μ: 6.01
σ: 2.83
H: 5.6
R²: 0.27
Najas.guadalupensis
μ: 7.37
σ: 5.65
H: 2.2
R²: 0.0067
Nelumbo.lutea
μ: 3.47
σ: 2.52
H: 3.6
R²: 0.082
Nitella
μ: 10
σ: 4.75
H: 5.6
R²: 0.074
Nuphar.advena
μ: 0
σ: 10
H: 2.8
R²: −0.00066
Nuphar.variegata
μ: 10
σ: 2.55
H: 3.7
R²: 0.19
Nymphaea.odorata
μ: 10
σ: 7.58
H: 14
R²: 0.053
Parthenocissus.quinquefolia
μ: 2.94
σ: 10
H: 3.3
R²: 9.6e−06
Persicaria.amphibia
μ: 5.85
σ: 3.84
H: 2.6
R²: 0.029
Probability
0.0 0.2 0.4 0.6 0.8 1.0
Environmental Condition (C_{env})
0 2 4 6 8 10

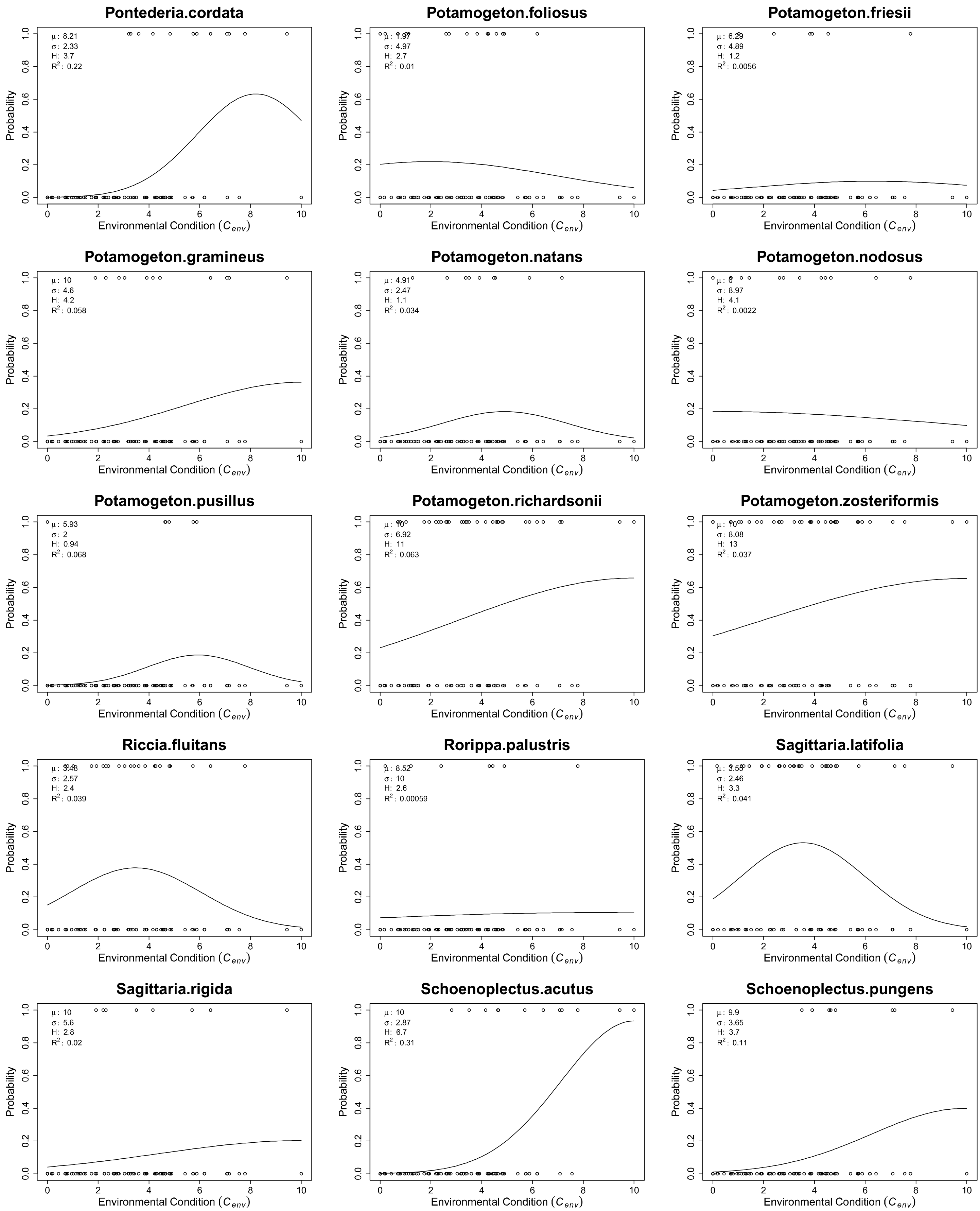

Pontederia.cordata
μ: 8.21
σ: 2.33
H: 3.7
R²: 0.22
Potamogeton.foliosus
μ: 1.97
σ: 4.97
H: 2.7
R²: 0.01
Potamogeton.friesii
μ: 6.29
σ: 4.89
H: 1.2
R²: 0.0056
Potamogeton.gramineus
μ: 10
σ: 4.6
H: 4.2
R²: 0.058
Potamogeton.natans
μ: 4.91
σ: 2.47
H: 1.1
R²: 0.034
Potamogeton.nodosus
μ: 0
σ: 8.97
H: 4.1
R²: 0.0022
Potamogeton.pusillus
μ: 5.93
σ: 2
H: 0.94
R²: 0.068
Potamogeton.richardsonii
μ: 10
σ: 6.92
H: 11
R²: 0.063
Potamogeton.zosteriformis
μ: 10
σ: 8.08
H: 13
R²: 0.037
Riccia.fluitans
μ: 3.48
σ: 2.57
H: 2.4
R²: 0.039
Rorippa.palustris
μ: 8.52
σ: 10
H: 2.6
R²: 0.00059
Sagittaria.latifolia
μ: 3.55
σ: 2.46
H: 3.3
R²: 0.041
Sagittaria.rigida
μ: 10
σ: 5.6
H: 2.8
R²: 0.02
Schoenoplectus.acutus
μ: 10
σ: 2.87
H: 6.7
R²: 0.31
Schoenoplectus.pungens
μ: 9.9
σ: 3.65
H: 3.7
R²: 0.11
Probability
Environmental Condition (C_{env})

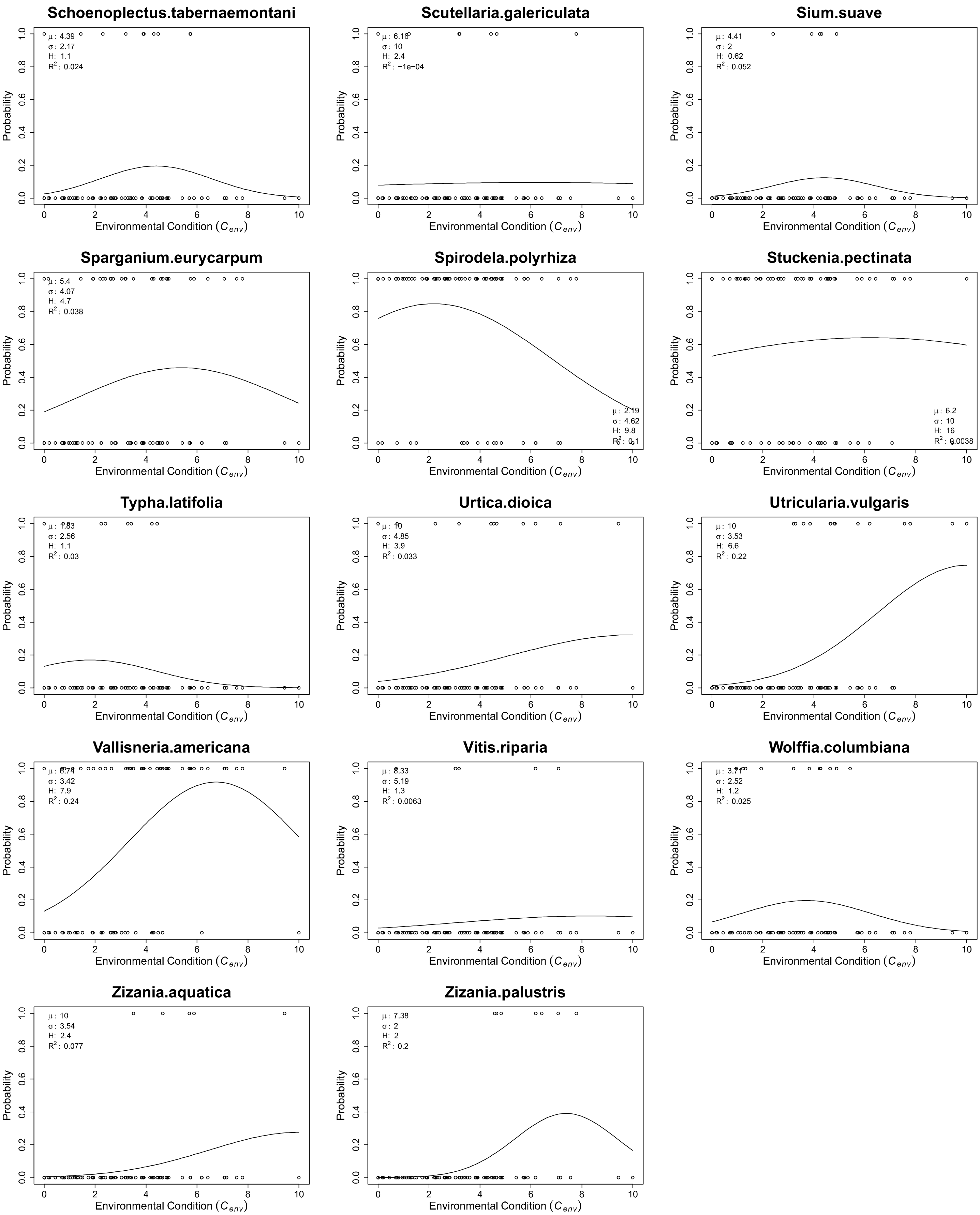

Schoenoplectus.tabernaemontani
μ: 4.39
σ: 2.17
H: 1.1
R^2: 0.024
Scutellaria.galericulata
σ: 10
H: 2.4
R^2: −1e−04
Sium.suave
μ: 4.41
σ: 2
H: 0.62
R^2: 0.052
Sparganium.eurycarpum
μ: 5.4
σ: 4.07
H: 4.7
R^2: 0.038
Spirodela.polyrhiza
μ: 2.19
σ: 4.62
H: 9.8
Stuckenia.pectinata
μ: 6.2
σ: 10
H: 16
Typha.latifolia
σ: 2.56
H: 1.1
R^2: 0.03
Urtica.dioica
σ: 4.85
H: 3.9
R^2: 0.033
Utricularia.vulgaris
μ: 10
σ: 3.53
H: 6.6
R^2: 0.22
Vallisneria.americana
σ: 3.42
H: 7.9
R^2: 0.24
Vitis.riparia
σ: 5.19
H: 1.3
R^2: 0.0063
Wolffia.columbiana
σ: 2.52
H: 1.2
R^2: 0.025
Zizania.aquatica
μ: 10
σ: 3.54
H: 2.4
R^2: 0.077
Zizania.palustris
μ: 7.38
σ: 2
H: 2
R^2: 0.2
Probability
Environmental Condition (C_{env})
