# Supplementary material for: Beyond Floristic Quality: A Data-Driven Approach to Assessing Coastal Wetland Condition Using Plants
Source: Wetlands (Wilmington). 2026 Oct 6;46(7):129. doi: 10.1007/s13157-026-02094-z (PMC13642451; doi:10.1007/s13157-026-02094-z)
Supplement: Supplementary file 2 [file 13157_2026_2094_MOESM2_ESM.pdf]

Wetlands

Online Resource 1: Lake Huron biotic response curves

Beyond floristic quality: A data-driven approach to assessing coastal wetland condition using plants

Giuseppe E. Fiorino[1]*, Simon T. Denomme-Brown[1], Hayley N. Rogers[1], Ian M. Smith[1], Kathryn L. Amatangelo[2], Matthew J. Cooper[3], Nicholas P. Danz[4], Robert W. Howe[5], Rachel E. Schultz[2], Bridget A. Wheelock[6], Douglas A. Wilcox[2], Greg P. Grabas[1]

[1]Environment and Climate Change Canada, 4905 Dufferin Street, Toronto, Ontario M3H 5T4, Canada

[2]Department of Environmental Science and Ecology, SUNY-Brockport, 350 New Campus Drive, Brockport, NY 14420, USA

[3]Department of Biology, Grand Valley State University, Allendale, MI 49401, USA

[4]Department of Natural Sciences, University of Wisconsin-Superior, 1605 Catlin Avenue, Superior, WI 54880, USA

[5]Cofrin Center for Biodiversity, University of Wisconsin-Green Bay, Green Bay, WI 54311-7001, USA

[6]Institute for Great Lakes Research and Department of Biology, Central Michigan University, Mount Pleasant, MI, USA

*Corresponding Author (email: giuseppe.fiorino@ec.gc.ca, ORCID: 0000-0002-1569-0767)

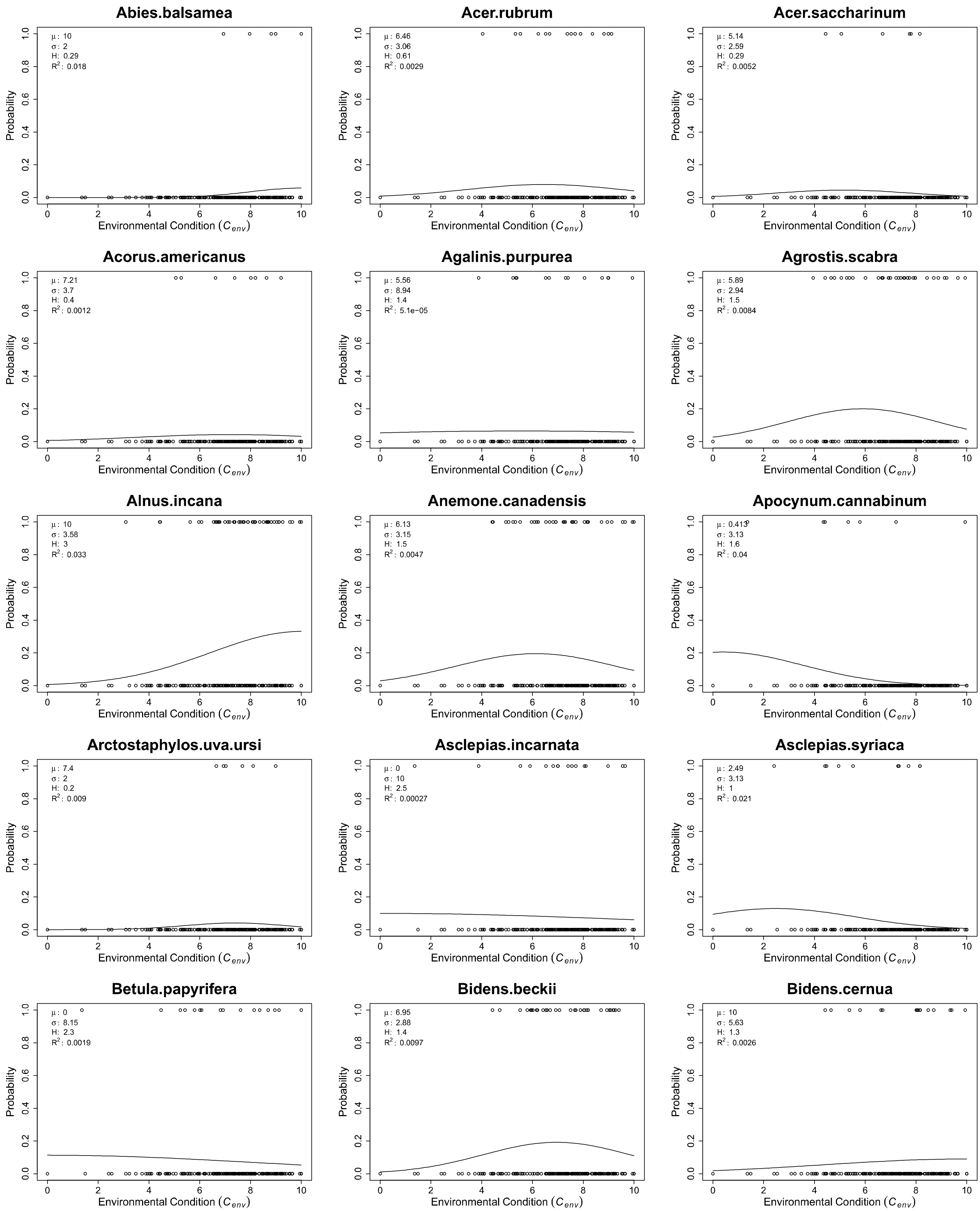
Abies.balsamea
μ: 10
σ: 2
H: 0.29
R²: 0.018
Acer.rubrum
μ: 6.46
σ: 3.06
H: 0.61
R²: 0.0029
Acer.saccharinum
μ: 5.14
σ: 2.59
H: 0.29
R²: 0.0052
Acorus.americanus
μ: 7.21
σ: 3.7
H: 0.4
R²: 0.0012
Agalinis.purpurea
μ: 5.56
σ: 8.94
H: 1.4
R²: 5.1e−05
Agrostis.scabra
μ: 5.89
σ: 2.94
H: 1.5
R²: 0.0084
Alnus.incana
μ: 10
σ: 3.58
H: 3
R²: 0.033
Anemone.canadensis
μ: 6.13
σ: 3.15
H: 1.5
R²: 0.0047
Apocynum.cannabinum
μ: 0.413
σ: 3.13
H: 1.6
R²: 0.04
Arctostaphylos.uva.ursi
μ: 7.4
σ: 2
H: 0.2
R²: 0.009
Asclepias.incarnata
μ: 0
σ: 10
H: 2.5
R²: 0.00027
Asclepias.syriaca
μ: 2.49
σ: 3.13
H: 1
R²: 0.021
Betula.papyrifera
μ: 0
σ: 8.15
H: 2.3
R²: 0.0019
Bidens.beckii
μ: 6.95
σ: 2.88
H: 1.4
R²: 0.0097
Bidens.cernua
μ: 10
σ: 5.63
H: 1.3
R²: 0.0026
Probability
Environmental Condition (Cenv)

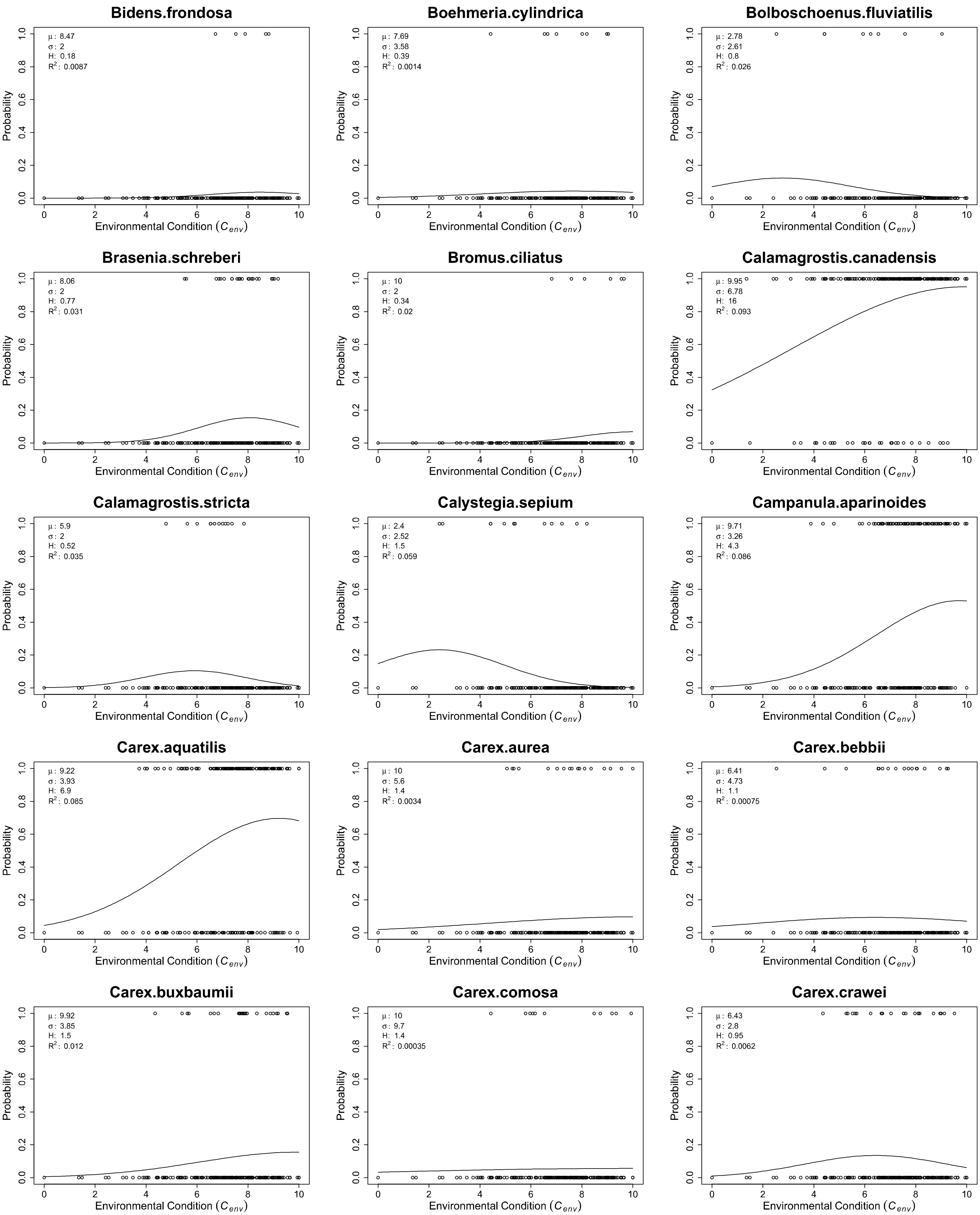
Bidens.frondosa
μ: 8.47
σ: 2
H: 0.18
R²: 0.0087
Boehmeria.cylindrica
μ: 7.69
σ: 3.58
H: 0.39
R²: 0.0014
Bolboschoenus.fluviatilis
μ: 2.78
σ: 2.61
H: 0.8
R²: 0.026
Brasenia.schreberi
μ: 8.06
σ: 2
H: 0.77
R²: 0.031
Bromus.ciliatus
μ: 10
σ: 2
H: 0.34
R²: 0.02
Calamagrostis.canadensis
μ: 9.95
σ: 6.78
H: 16
R²: 0.093
Calamagrostis.stricta
μ: 5.9
σ: 2
H: 0.52
R²: 0.035
Calystegia.sepium
μ: 2.4
σ: 2.52
H: 1.5
R²: 0.059
Campanula.aparinoides
μ: 9.71
σ: 3.26
H: 4.3
R²: 0.086
Carex.aquatilis
μ: 9.22
σ: 3.93
H: 6.9
R²: 0.085
Carex.aurea
μ: 10
σ: 5.6
H: 1.4
R²: 0.0034
Carex.bebbii
μ: 6.41
σ: 4.73
H: 1.1
R²: 0.00075
Carex.buxbaumii
μ: 9.92
σ: 3.85
H: 1.5
R²: 0.012
Carex.comosa
μ: 10
σ: 9.7
H: 1.4
R²: 0.00035
Carex.crawei
μ: 6.43
σ: 2.8
H: 0.95
R²: 0.0062
Probability
Environmental Condition (C_{env})

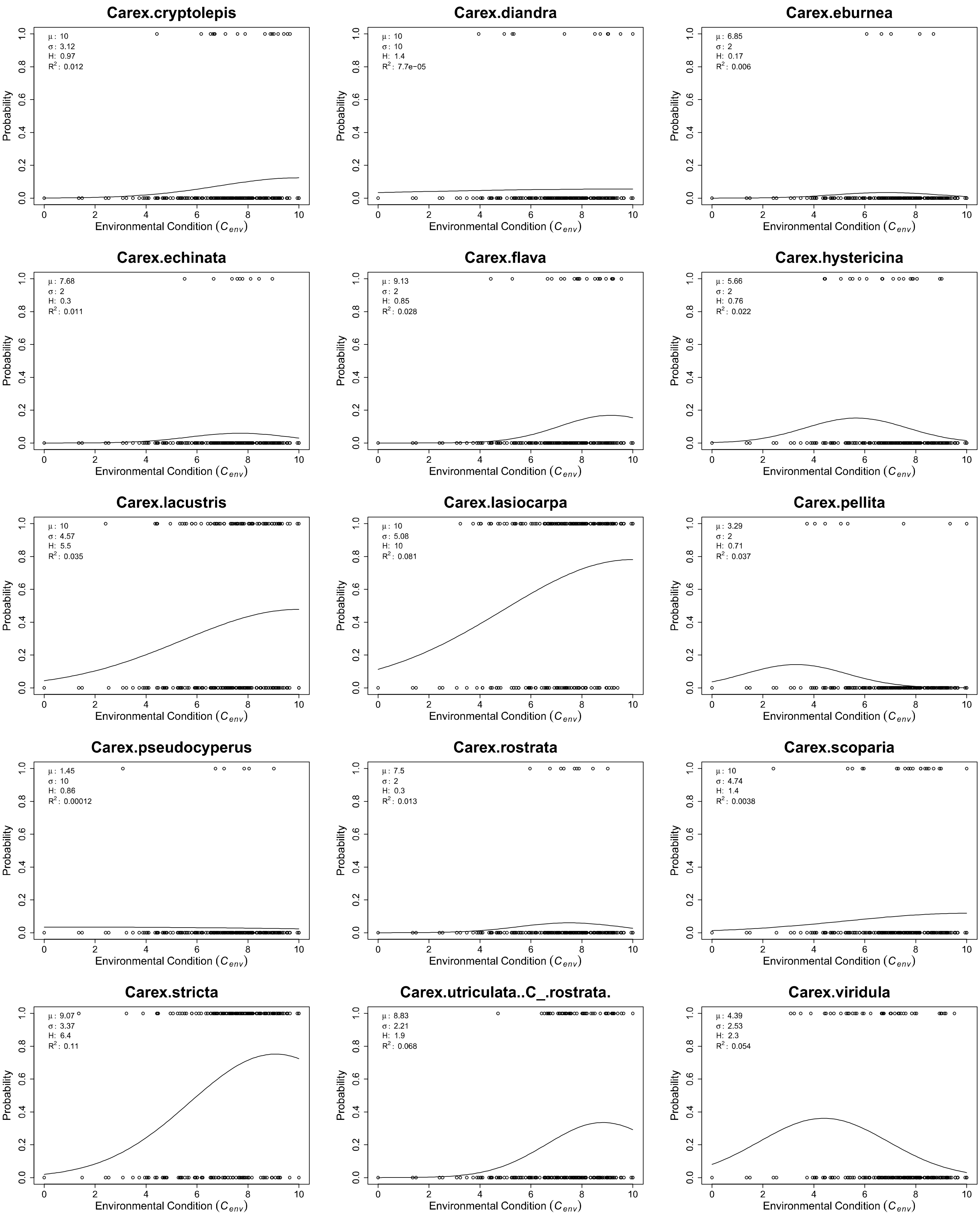

Carex.cryptolepis
μ: 10
σ: 3.12
H: 0.97
R²: 0.012
Carex.diandra
μ: 10
σ: 10
H: 1.4
R²: 7.7e−05
Carex.eburnea
μ: 6.85
σ: 2
H: 0.17
R²: 0.006
Carex.echinata
μ: 7.68
σ: 2
H: 0.3
R²: 0.011
Carex.flava
μ: 9.13
σ: 2
H: 0.85
R²: 0.028
Carex.hystericina
μ: 5.66
σ: 2
H: 0.76
R²: 0.022
Carex.lacustris
μ: 10
σ: 4.57
H: 5.5
R²: 0.035
Carex.lasiocarpa
μ: 10
σ: 5.08
H: 10
R²: 0.081
Carex.pellita
μ: 3.29
σ: 2
H: 0.71
R²: 0.037
Carex.pseudocyperus
μ: 1.45
σ: 10
H: 0.86
R²: 0.00012
Carex.rostrata
μ: 7.5
σ: 2
H: 0.3
R²: 0.013
Carex.scoparia
μ: 10
σ: 4.74
H: 1.4
R²: 0.0038
Carex.stricta
μ: 9.07
σ: 3.37
H: 6.4
R²: 0.11
Carex.utriculata..C_.rostrata.
μ: 8.83
σ: 2.21
H: 1.9
R²: 0.068
Carex.viridula
μ: 4.39
σ: 2.53
H: 2.3
R²: 0.054
Probability
Environmental Condition (C_{env})

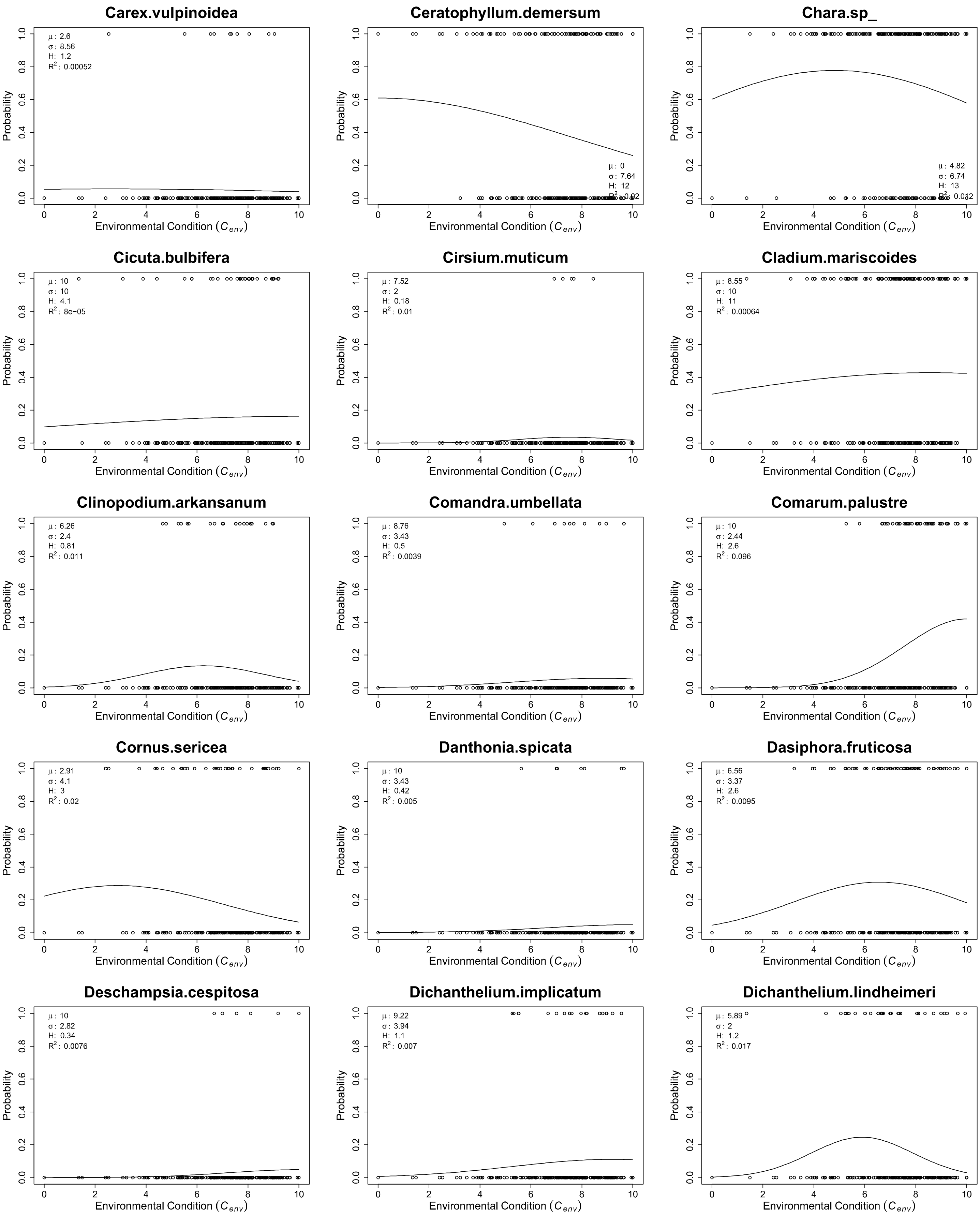
Carex.vulpinoidea
μ: 2.6
σ: 8.56
H: 1.2
R²: 0.00052
Ceratophyllum.demersum
μ: 0
σ: 7.64
H: 12
Chara.sp_
μ: 4.82
σ: 6.74
H: 13
Cicuta.bulbifera
μ: 10
σ: 10
H: 4.1
R²: 8e−05
Cirsium.muticum
μ: 7.52
σ: 2
H: 0.18
R²: 0.01
Cladium.mariscoides
μ: 8.55
σ: 10
H: 11
R²: 0.00064
Clinopodium.arkansanum
μ: 6.26
σ: 2.4
H: 0.81
R²: 0.011
Comandra.umbellata
μ: 8.76
σ: 3.43
H: 0.5
R²: 0.0039
Comarum.palustre
μ: 10
σ: 2.44
H: 2.6
R²: 0.096
Cornus.sericea
μ: 2.91
σ: 4.1
H: 3
R²: 0.02
Danthonia.spicata
μ: 10
σ: 3.43
H: 0.42
R²: 0.005
Dasiphora.fruticosa
μ: 6.56
σ: 3.37
H: 2.6
R²: 0.0095
Deschampsia.cespitosa
μ: 10
σ: 2.82
H: 0.34
R²: 0.0076
Dichanthelium.implicatum
μ: 9.22
σ: 3.94
H: 1.1
R²: 0.007
Dichanthelium.lindheimeri
μ: 5.89
σ: 2
H: 1.2
R²: 0.017
Probability
Environmental Condition (C_{env})

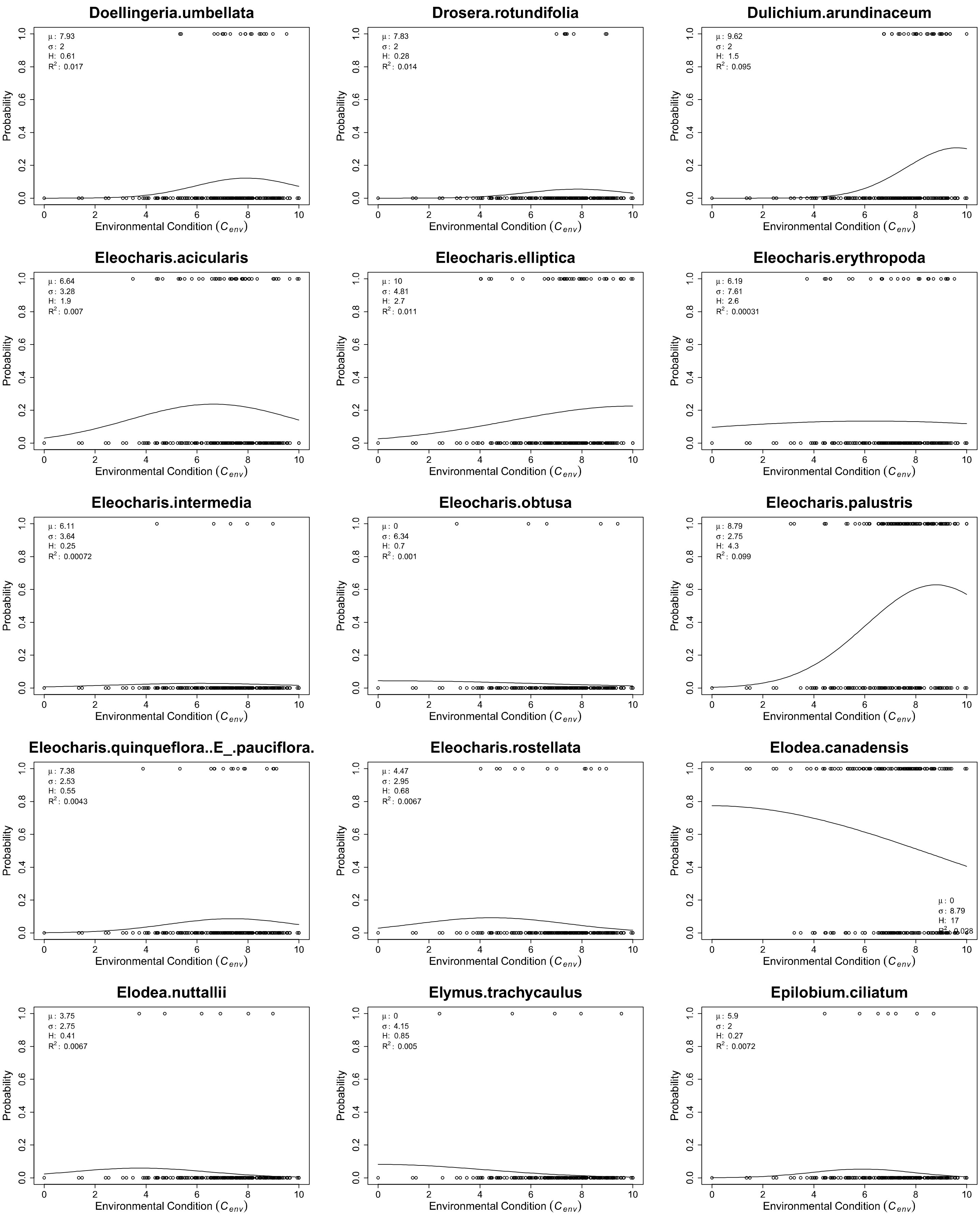
Doellingeria.umbellata
μ: 7.93
σ: 2
H: 0.61
R2: 0.017
Drosera.rotundifolia
μ: 7.83
σ: 2
H: 0.28
R2: 0.014
Dulichium.arundinaceum
μ: 9.62
σ: 2
H: 1.5
R2: 0.095
Eleocharis.acicularis
μ: 6.64
σ: 3.28
H: 1.9
R2: 0.007
Eleocharis.elliptica
μ: 10
σ: 4.81
H: 2.7
R2: 0.011
Eleocharis.erythropoda
μ: 6.19
σ: 7.61
H: 2.6
R2: 0.00031
Eleocharis.intermedia
μ: 6.11
σ: 3.64
H: 0.25
R2: 0.00072
Eleocharis.obtusa
μ: 0
σ: 6.34
H: 0.7
R2: 0.001
Eleocharis.palustris
μ: 8.79
σ: 2.75
H: 4.3
R2: 0.099
Eleocharis.quinqueflora..E_.pauciflora.
μ: 7.38
σ: 2.53
H: 0.55
R2: 0.0043
Eleocharis.rostellata
μ: 4.47
σ: 2.95
H: 0.68
R2: 0.0067
Elodea.canadensis
μ: 0
σ: 8.79
H: 17
R2: 0.028
Elodea.nuttallii
μ: 3.75
σ: 2.75
H: 0.41
R2: 0.0067
Elymus.trachycaulus
μ: 0
σ: 4.15
H: 0.85
R2: 0.005
Epilobium.ciliatum
μ: 5.9
σ: 2
H: 0.27
R2: 0.0072
Probability
Environmental Condition (C_{env})
0.0 0.2 0.4 0.6 0.8 1.0
0 2 4 6 8 10

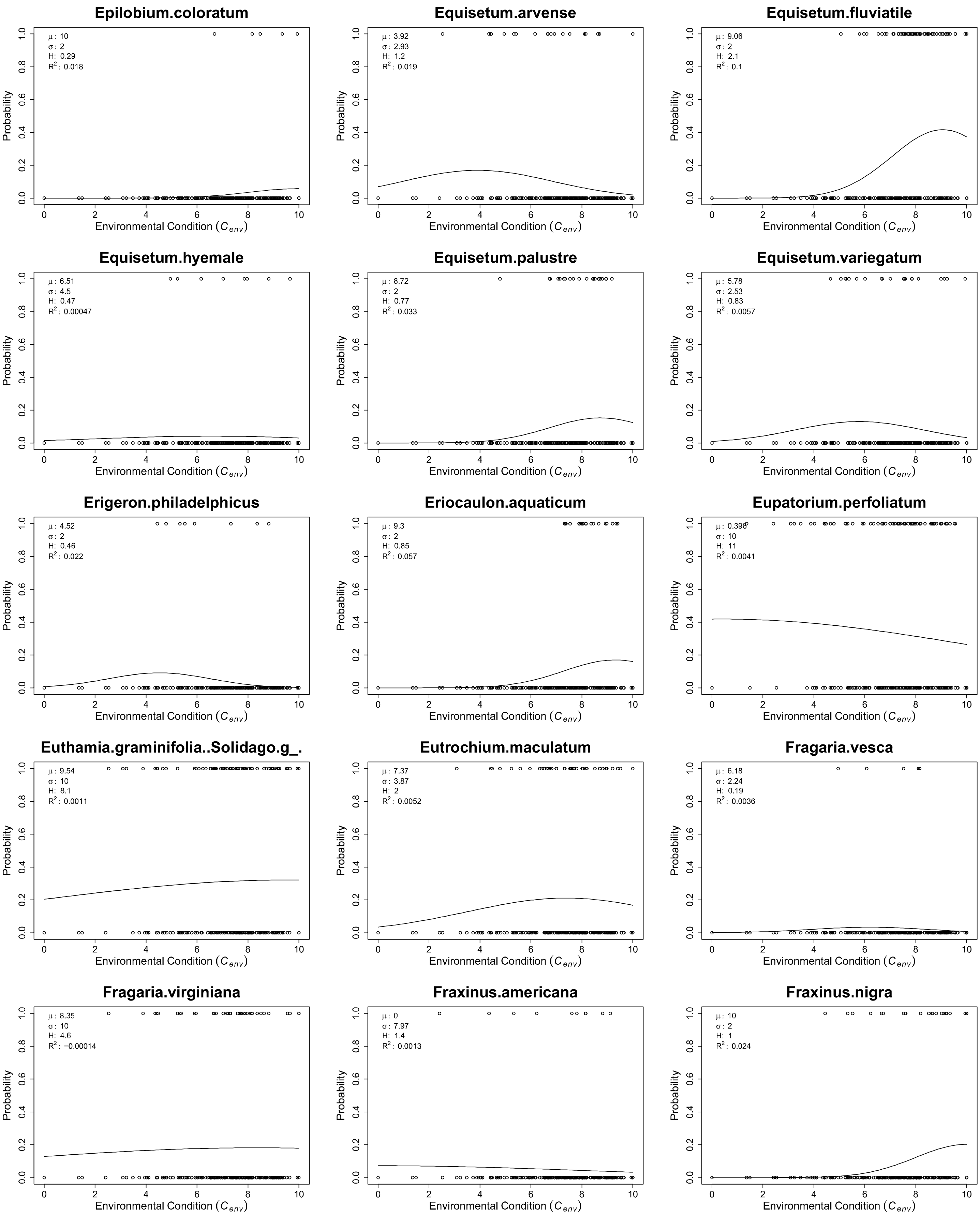
Epilobium.coloratum
μ: 10
σ: 2
H: 0.29
R²: 0.018
Equisetum.arvense
μ: 3.92
σ: 2.93
H: 1.2
R²: 0.019
Equisetum.fluviatile
μ: 9.06
σ: 2
H: 2.1
R²: 0.1
Equisetum.hyemale
μ: 6.51
σ: 4.5
H: 0.47
R²: 0.00047
Equisetum.palustre
μ: 8.72
σ: 2
H: 0.77
R²: 0.033
Equisetum.variegatum
μ: 5.78
σ: 2.53
H: 0.83
R²: 0.0057
Erigeron.philadelphicus
μ: 4.52
σ: 2
H: 0.46
R²: 0.022
Eriocaulon.aquaticum
μ: 9.3
σ: 2
H: 0.85
R²: 0.057
Eupatorium.perfoliatum
μ: 0.396
σ: 10
H: 11
R²: 0.0041
Euthamia.graminifolia..Solidago.g_.
μ: 9.54
σ: 10
H: 8.1
R²: 0.0011
Eutrochium.maculatum
μ: 7.37
σ: 3.87
H: 2
R²: 0.0052
Fragaria.vesca
μ: 6.18
σ: 2.24
H: 0.19
R²: 0.0036
Fragaria.virginiana
μ: 8.35
σ: 10
H: 4.6
R²: −0.00014
Fraxinus.americana
μ: 0
σ: 7.97
H: 1.4
R²: 0.0013
Fraxinus.nigra
μ: 10
σ: 2
H: 1
R²: 0.024
Probability
Environmental Condition (C_{env})

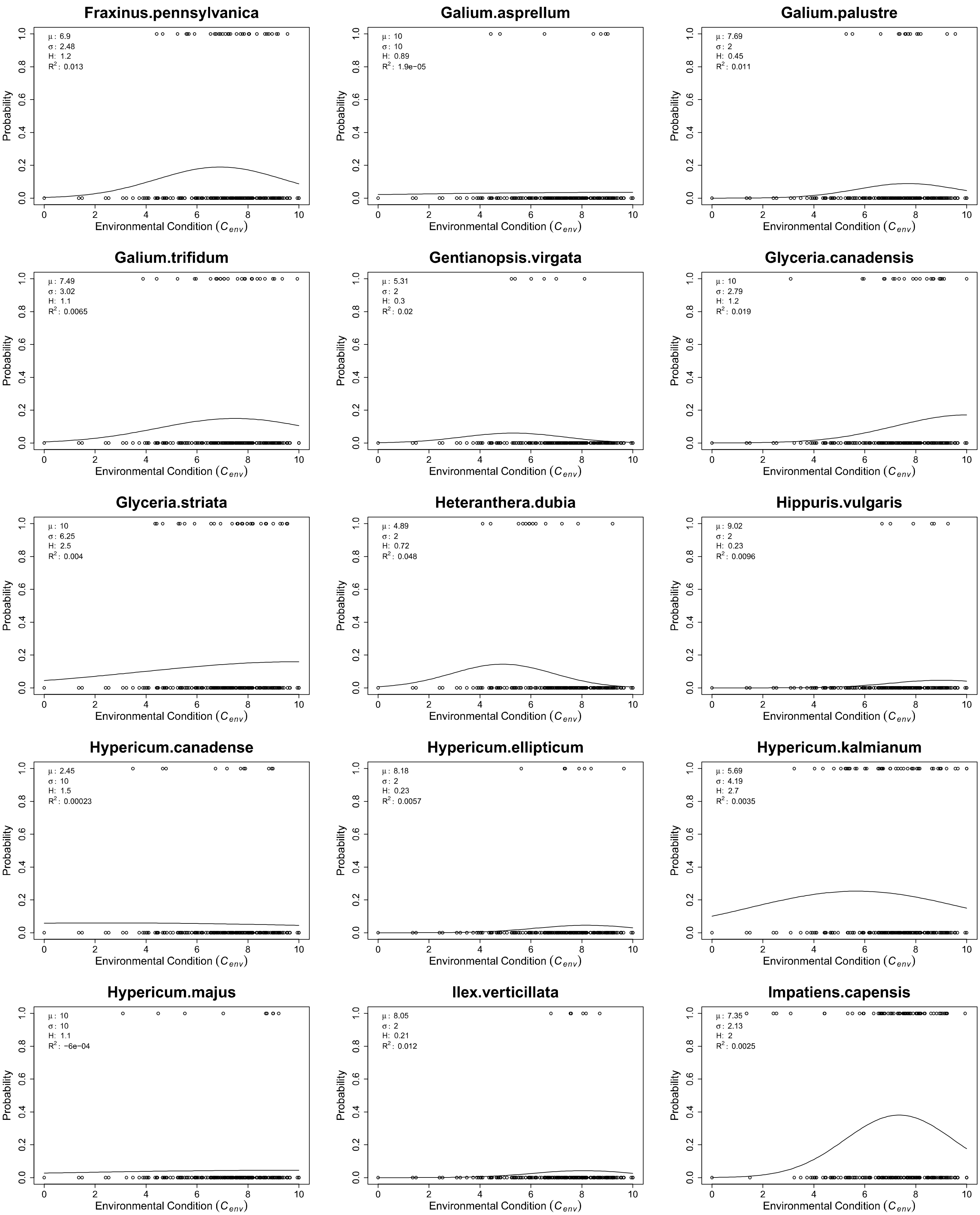
Fraxinus.pennsylvanica
μ: 6.9
σ: 2.48
H: 1.2
R²: 0.013
Galium.asprellum
μ: 10
σ: 10
H: 0.89
R²: 1.9e−05
Galium.palustre
μ: 7.69
σ: 2
H: 0.45
R²: 0.011
Galium.trifidum
μ: 7.49
σ: 3.02
H: 1.1
R²: 0.0065
Gentianopsis.virgata
μ: 5.31
σ: 2
H: 0.3
R²: 0.02
Glyceria.canadensis
μ: 10
σ: 2.79
H: 1.2
R²: 0.019
Glyceria.striata
μ: 10
σ: 6.25
H: 2.5
R²: 0.004
Heteranthera.dubia
μ: 4.89
σ: 2
H: 0.72
R²: 0.048
Hippuris.vulgaris
μ: 9.02
σ: 2
H: 0.23
R²: 0.0096
Hypericum.canadense
μ: 2.45
σ: 10
H: 1.5
R²: 0.00023
Hypericum.ellipticum
μ: 8.18
σ: 2
H: 0.23
R²: 0.0057
Hypericum.kalmianum
μ: 5.69
σ: 4.19
H: 2.7
R²: 0.0035
Hypericum.majus
μ: 10
σ: 10
H: 1.1
R²: −6e−04
Ilex.verticillata
μ: 8.05
σ: 2
H: 0.21
R²: 0.012
Impatiens.capensis
μ: 7.35
σ: 2.13
H: 2
R²: 0.0025
Probability
Environmental Condition (C_env)

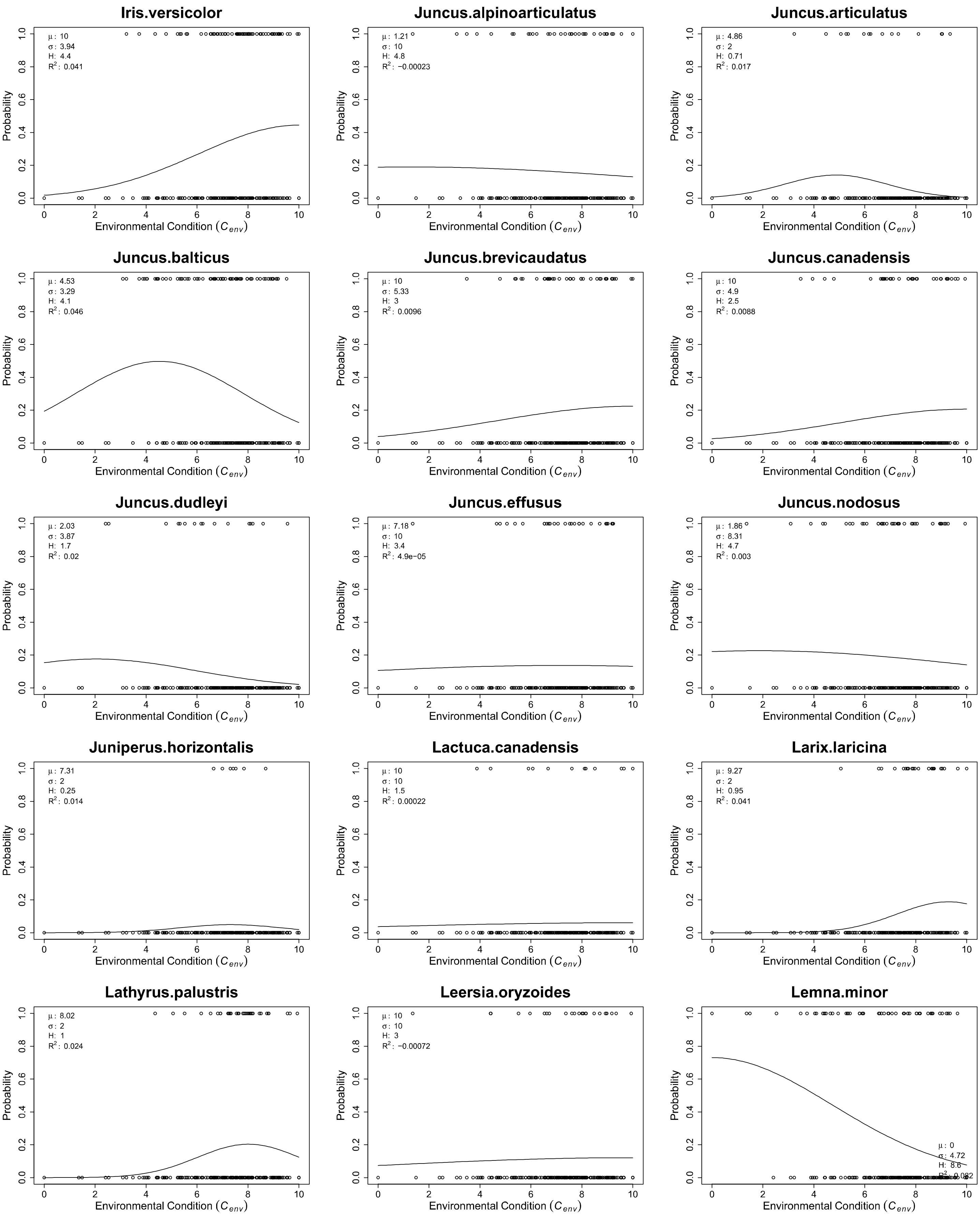

Iris.versicolor
μ: 10
σ: 3.94
H: 4.4
R²: 0.041
Juncus.alpinoarticulatus
μ: 1.21
σ: 10
H: 4.8
R²: −0.00023
Juncus.articulatus
μ: 4.86
σ: 2
H: 0.71
R²: 0.017
Juncus.balticus
μ: 4.53
σ: 3.29
H: 4.1
R²: 0.046
Juncus.brevicaudatus
μ: 10
σ: 5.33
H: 3
R²: 0.0096
Juncus.canadensis
μ: 10
σ: 4.9
H: 2.5
R²: 0.0088
Juncus.dudleyi
μ: 2.03
σ: 3.87
H: 1.7
R²: 0.02
Juncus.effusus
μ: 7.18
σ: 10
H: 3.4
R²: 4.9e−05
Juncus.nodosus
μ: 1.86
σ: 8.31
H: 4.7
R²: 0.003
Juniperus.horizontalis
μ: 7.31
σ: 2
H: 0.25
R²: 0.014
Lactuca.canadensis
μ: 10
σ: 10
H: 1.5
R²: 0.00022
Larix.laricina
μ: 9.27
σ: 2
H: 0.95
R²: 0.041
Lathyrus.palustris
μ: 8.02
σ: 2
H: 1
R²: 0.024
Leersia.oryzoides
μ: 10
σ: 10
H: 3
R²: −0.00072
Lemna.minor
μ: 0
σ: 4.72
H: 8.6
R²: 0.082
Probability
Environmental Condition (C_{env})
0.0
0.2
0.4
0.6
0.8
1.0
0
2
4
6
8
10

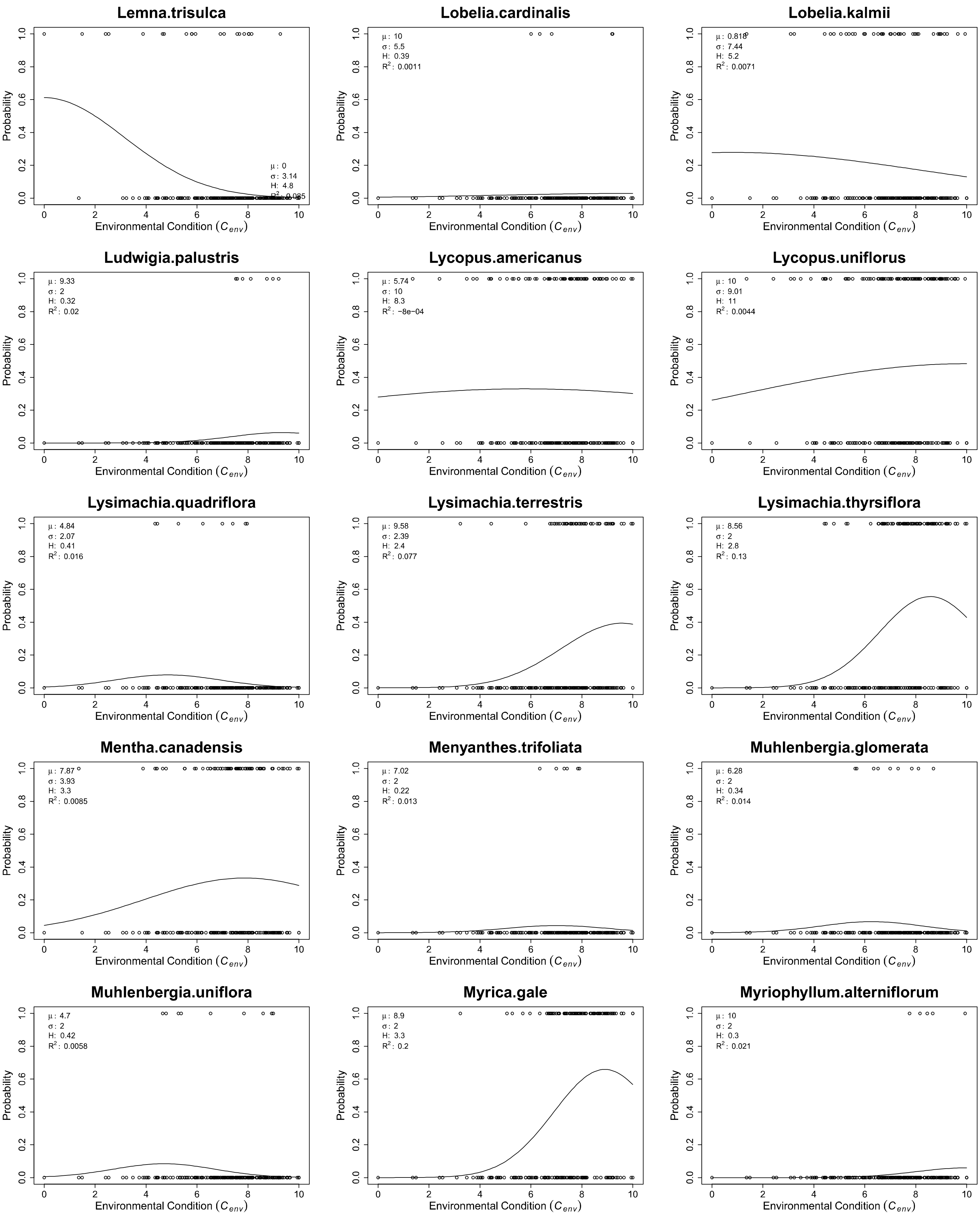
Lemna.trisulca
μ: 0
σ: 3.14
H: 4.8
R²: 0.085
Lobelia.cardinalis
μ: 10
σ: 5.5
H: 0.39
R²: 0.0011
Lobelia.kalmii
μ: 0.818
σ: 7.44
H: 5.2
R²: 0.0071
Ludwigia.palustris
μ: 9.33
σ: 2
H: 0.32
R²: 0.02
Lycopus.americanus
μ: 5.74
σ: 10
H: 8.3
R²: -8e-04
Lycopus.uniflorus
μ: 10
σ: 9.01
H: 11
R²: 0.0044
Lysimachia.quadriflora
μ: 4.84
σ: 2.07
H: 0.41
R²: 0.016
Lysimachia.terrestris
μ: 9.58
σ: 2.39
H: 2.4
R²: 0.077
Lysimachia.thyrsiflora
μ: 8.56
σ: 2
H: 2.8
R²: 0.13
Mentha.canadensis
μ: 7.87
σ: 3.93
H: 3.3
R²: 0.0085
Menyanthes.trifoliata
μ: 7.02
σ: 2
H: 0.22
R²: 0.013
Muhlenbergia.glomerata
μ: 6.28
σ: 2
H: 0.34
R²: 0.014
Muhlenbergia.uniflora
μ: 4.7
σ: 2
H: 0.42
R²: 0.0058
Myrica.gale
μ: 8.9
σ: 2
H: 3.3
R²: 0.2
Myriophyllum.alterniflorum
μ: 10
σ: 2
H: 0.3
R²: 0.021
Probability
Environmental Condition (C_{env})

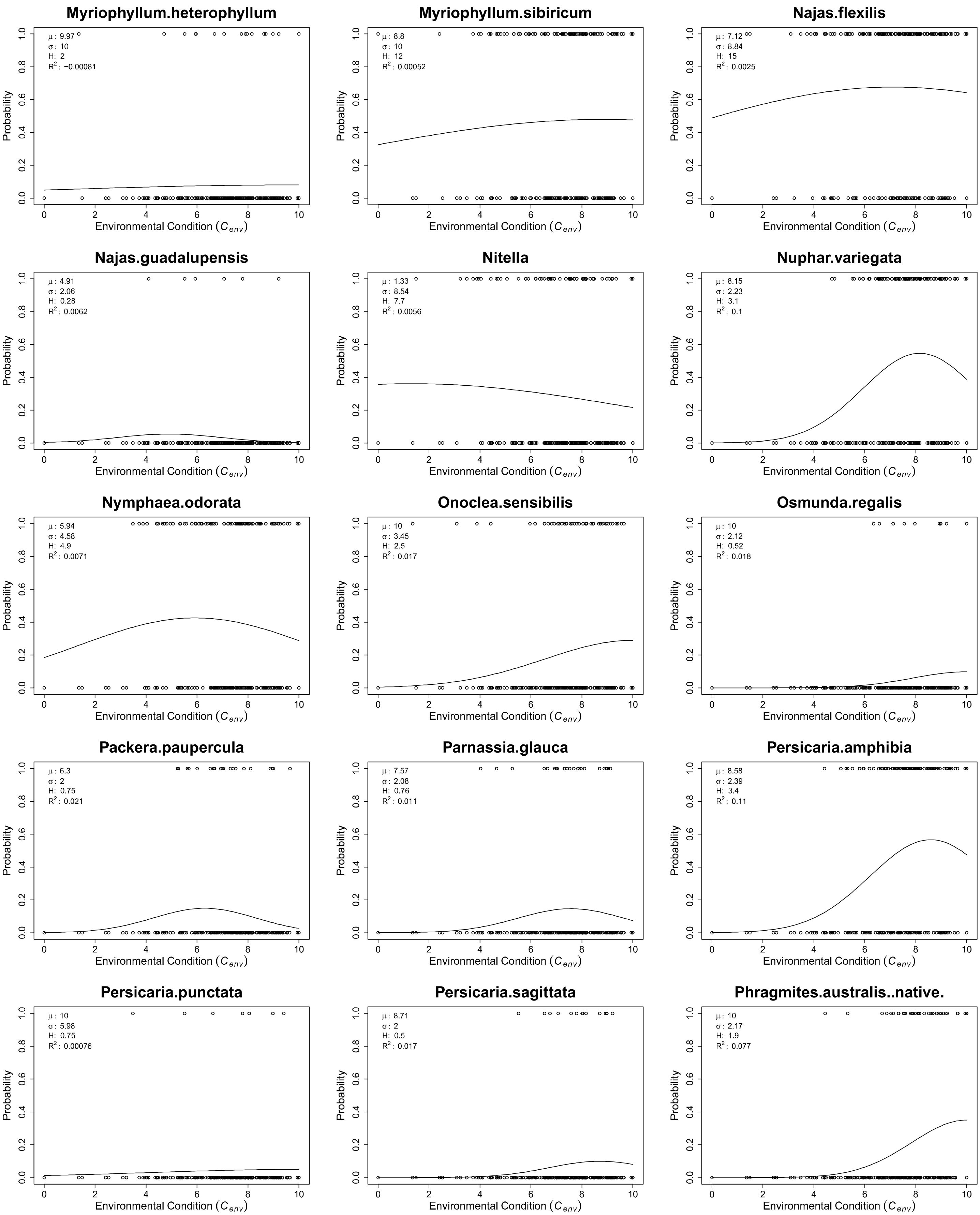

Myriophyllum.heterophyllum
μ: 9.97
σ: 10
H: 2
R²: −0.00081
Myriophyllum.sibiricum
μ: 8.8
σ: 10
H: 12
R²: 0.00052
Najas.flexilis
μ: 7.12
σ: 8.84
H: 15
R²: 0.0025
Najas.guadalupensis
μ: 4.91
σ: 2.06
H: 0.28
R²: 0.0062
Nitella
μ: 1.33
σ: 8.54
H: 7.7
R²: 0.0056
Nuphar.variegata
μ: 8.15
σ: 2.23
H: 3.1
R²: 0.1
Nymphaea.odorata
μ: 5.94
σ: 4.58
H: 4.9
R²: 0.0071
Onoclea.sensibilis
μ: 10
σ: 3.45
H: 2.5
R²: 0.017
Osmunda.regalis
μ: 10
σ: 2.12
H: 0.52
R²: 0.018
Packera.paupercula
μ: 6.3
σ: 2
H: 0.75
R²: 0.021
Parnassia.glauca
μ: 7.57
σ: 2.08
H: 0.76
R²: 0.011
Persicaria.amphibia
μ: 8.58
σ: 2.39
H: 3.4
R²: 0.11
Persicaria.punctata
μ: 10
σ: 5.98
H: 0.75
R²: 0.00076
Persicaria.sagittata
μ: 8.71
σ: 2
H: 0.5
R²: 0.017
Phragmites.australis..native.
μ: 10
σ: 2.17
H: 1.9
R²: 0.077
Probability
Environmental Condition (C_{env})

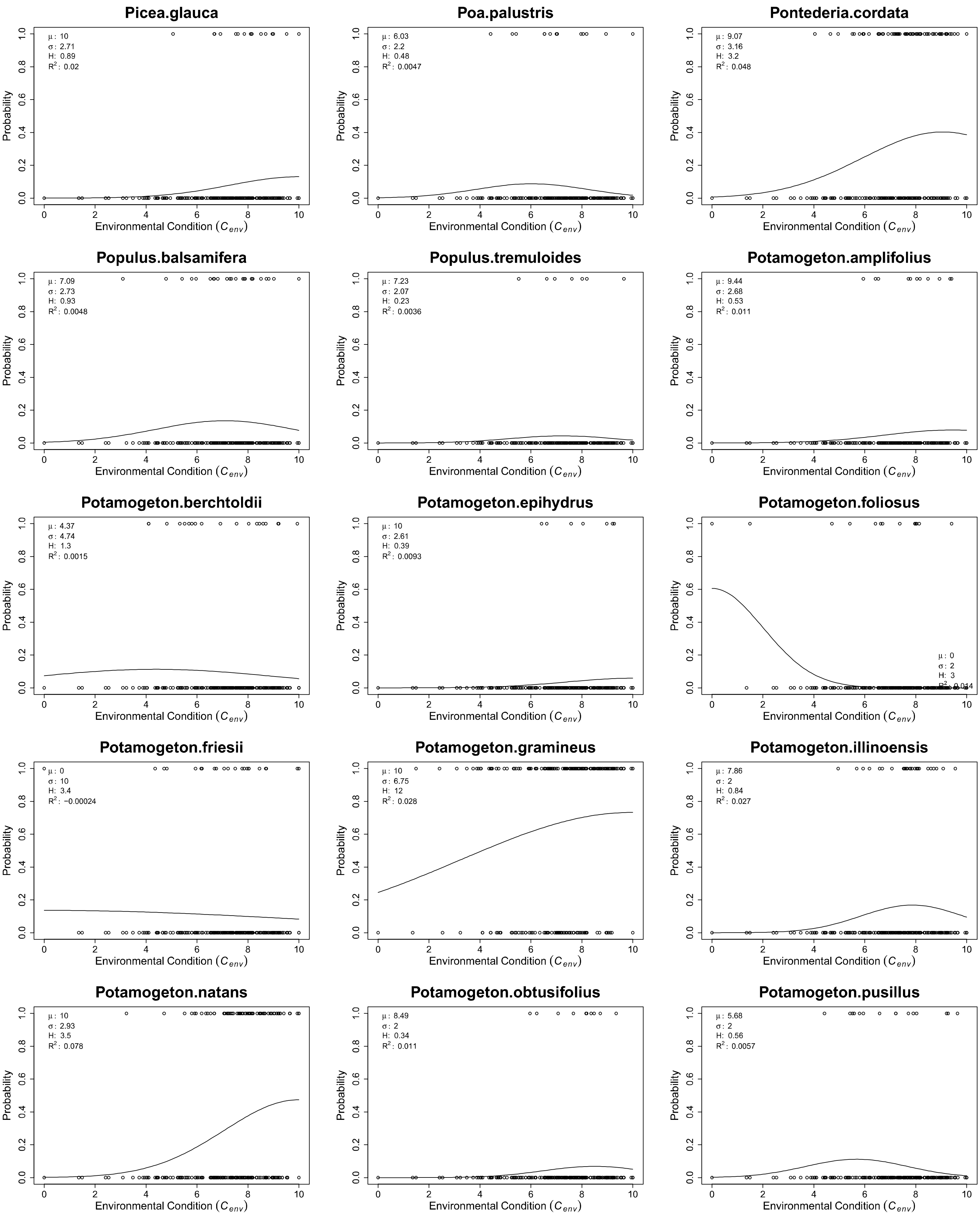

Picea.glauca
μ: 10
σ: 2.71
H: 0.89
R2: 0.02
Poa.palustris
μ: 6.03
σ: 2.2
H: 0.48
R2: 0.0047
Pontederia.cordata
μ: 9.07
σ: 3.16
H: 3.2
R2: 0.048
Populus.balsamifera
μ: 7.09
σ: 2.73
H: 0.93
R2: 0.0048
Populus.tremuloides
μ: 7.23
σ: 2.07
H: 0.23
R2: 0.0036
Potamogeton.amplifolius
μ: 9.44
σ: 2.68
H: 0.53
R2: 0.011
Potamogeton.berchtoldii
μ: 4.37
σ: 4.74
H: 1.3
R2: 0.0015
Potamogeton.epihydrus
μ: 10
σ: 2.61
H: 0.39
R2: 0.0093
Potamogeton.foliosus
μ: 0
σ: 2
H: 3
R2: 0.014
Potamogeton.friesii
μ: 0
σ: 10
H: 3.4
R2: −0.00024
Potamogeton.gramineus
μ: 10
σ: 6.75
H: 12
R2: 0.028
Potamogeton.illinoensis
μ: 7.86
σ: 2
H: 0.84
R2: 0.027
Potamogeton.natans
μ: 10
σ: 2.93
H: 3.5
R2: 0.078
Potamogeton.obtusifolius
μ: 8.49
σ: 2
H: 0.34
R2: 0.011
Potamogeton.pusillus
μ: 5.68
σ: 2
H: 0.56
R2: 0.0057
Probability
Environmental Condition (Cenv)

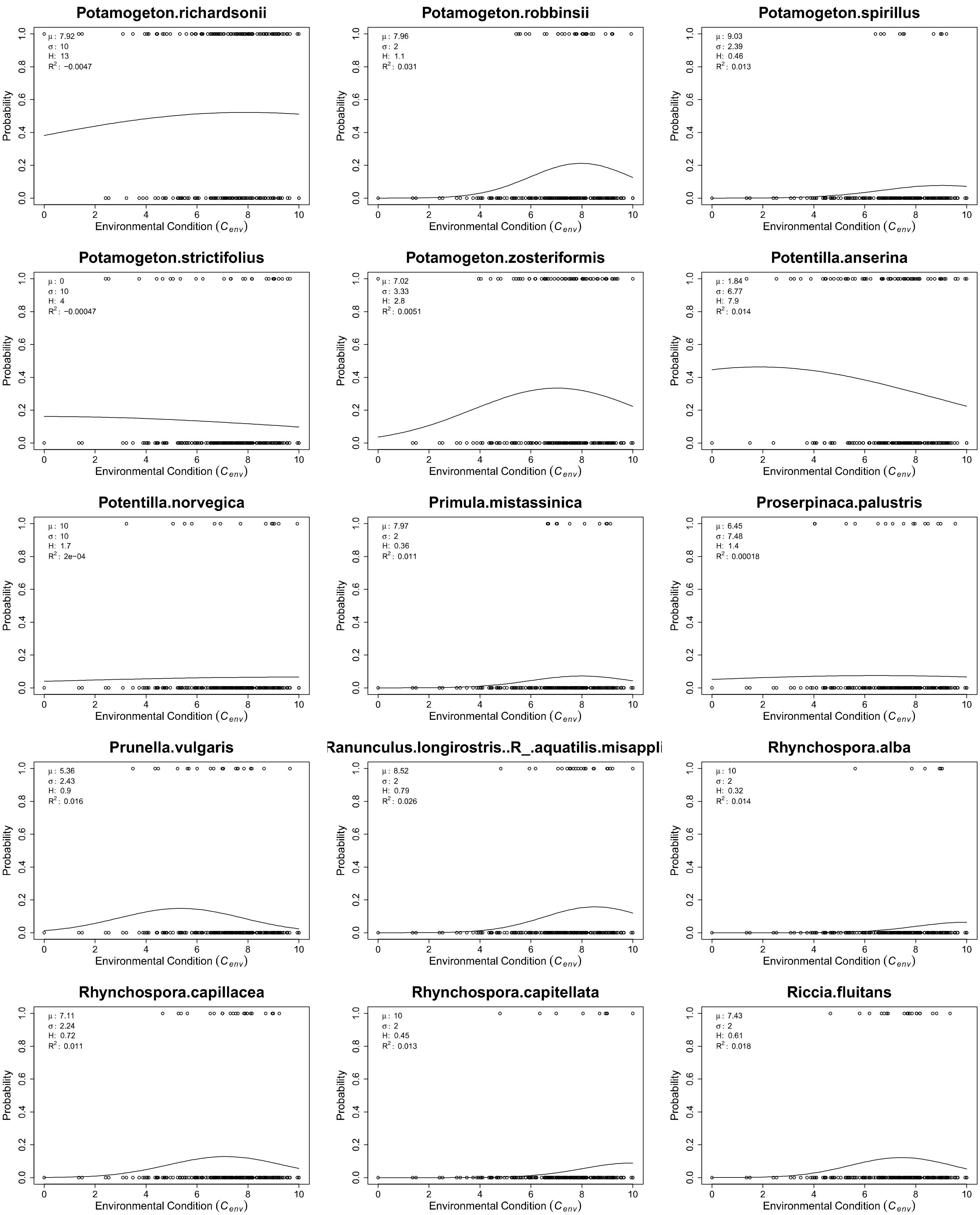

Potamogeton.richardsonii
μ: 7.92
σ: 10
H: 13
R²: −0.0047
Potamogeton.robbinsii
μ: 7.96
σ: 2
H: 1.1
R²: 0.031
Potamogeton.spirillus
μ: 9.03
σ: 2.39
H: 0.46
R²: 0.013
Potamogeton.strictifolius
μ: 0
σ: 10
H: 4
R²: −0.00047
Potamogeton.zosteriformis
μ: 7.02
σ: 3.33
H: 2.8
R²: 0.0051
Potentilla.anserina
μ: 1.84
σ: 6.77
H: 7.9
R²: 0.014
Potentilla.norvegica
μ: 10
σ: 10
H: 1.7
R²: 2e−04
Primula.mistassinica
μ: 7.97
σ: 2
H: 0.36
R²: 0.011
Proserpinaca.palustris
μ: 6.45
σ: 7.48
H: 1.4
R²: 0.00018
Prunella.vulgaris
μ: 5.36
σ: 2.43
H: 0.9
R²: 0.016
Ranunculus.longirostris..R_.aquatilis.misappli
μ: 8.52
σ: 2
H: 0.79
R²: 0.026
Rhynchospora.alba
μ: 10
σ: 2
H: 0.32
R²: 0.014
Rhynchospora.capillacea
μ: 7.11
σ: 2.24
H: 0.72
R²: 0.011
Rhynchospora.capitellata
μ: 10
σ: 2
H: 0.45
R²: 0.013
Riccia.fluitans
μ: 7.43
σ: 2
H: 0.61
R²: 0.018
Probability
Environmental Condition (C_{env})

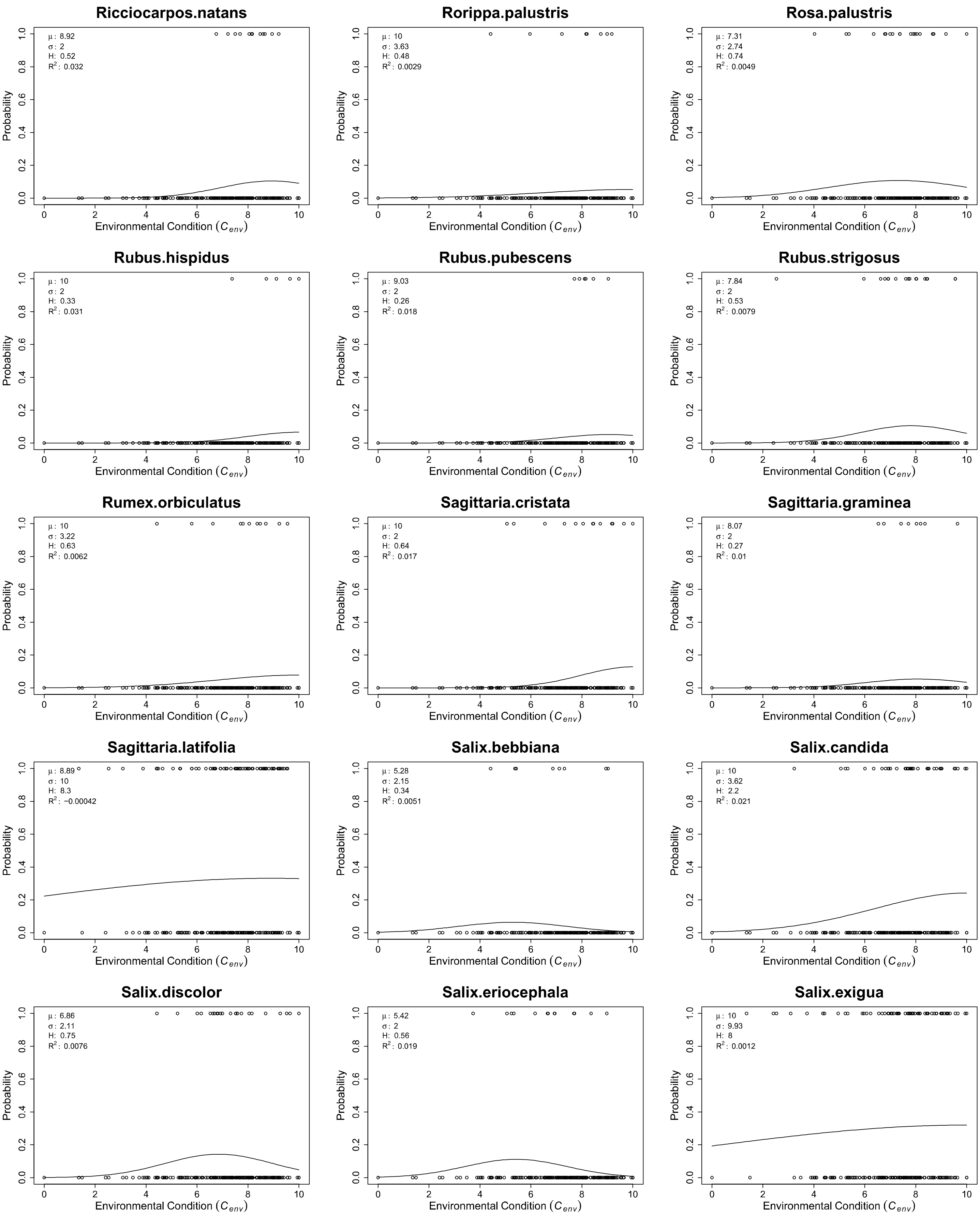

Ricciocarpos.natans
μ: 8.92
σ: 2
H: 0.52
R²: 0.032
Rorippa.palustris
μ: 10
σ: 3.63
H: 0.48
R²: 0.0029
Rosa.palustris
μ: 7.31
σ: 2.74
H: 0.74
R²: 0.0049
Rubus.hispidus
μ: 10
σ: 2
H: 0.33
R²: 0.031
Rubus.pubescens
μ: 9.03
σ: 2
H: 0.26
R²: 0.018
Rubus.strigosus
μ: 7.84
σ: 2
H: 0.53
R²: 0.0079
Rumex.orbiculatus
μ: 10
σ: 3.22
H: 0.63
R²: 0.0062
Sagittaria.cristata
μ: 10
σ: 2
H: 0.64
R²: 0.017
Sagittaria.graminea
μ: 8.07
σ: 2
H: 0.27
R²: 0.01
Sagittaria.latifolia
μ: 8.89
σ: 10
H: 8.3
R²: −0.00042
Salix.bebbiana
μ: 5.28
σ: 2.15
H: 0.34
R²: 0.0051
Salix.candida
μ: 10
σ: 3.62
H: 2.2
R²: 0.021
Salix.discolor
μ: 6.86
σ: 2.11
H: 0.75
R²: 0.0076
Salix.eriocephala
μ: 5.42
σ: 2
H: 0.56
R²: 0.019
Salix.exigua
μ: 10
σ: 9.93
H: 8
R²: 0.0012
Probability
Environmental Condition (C_{env})

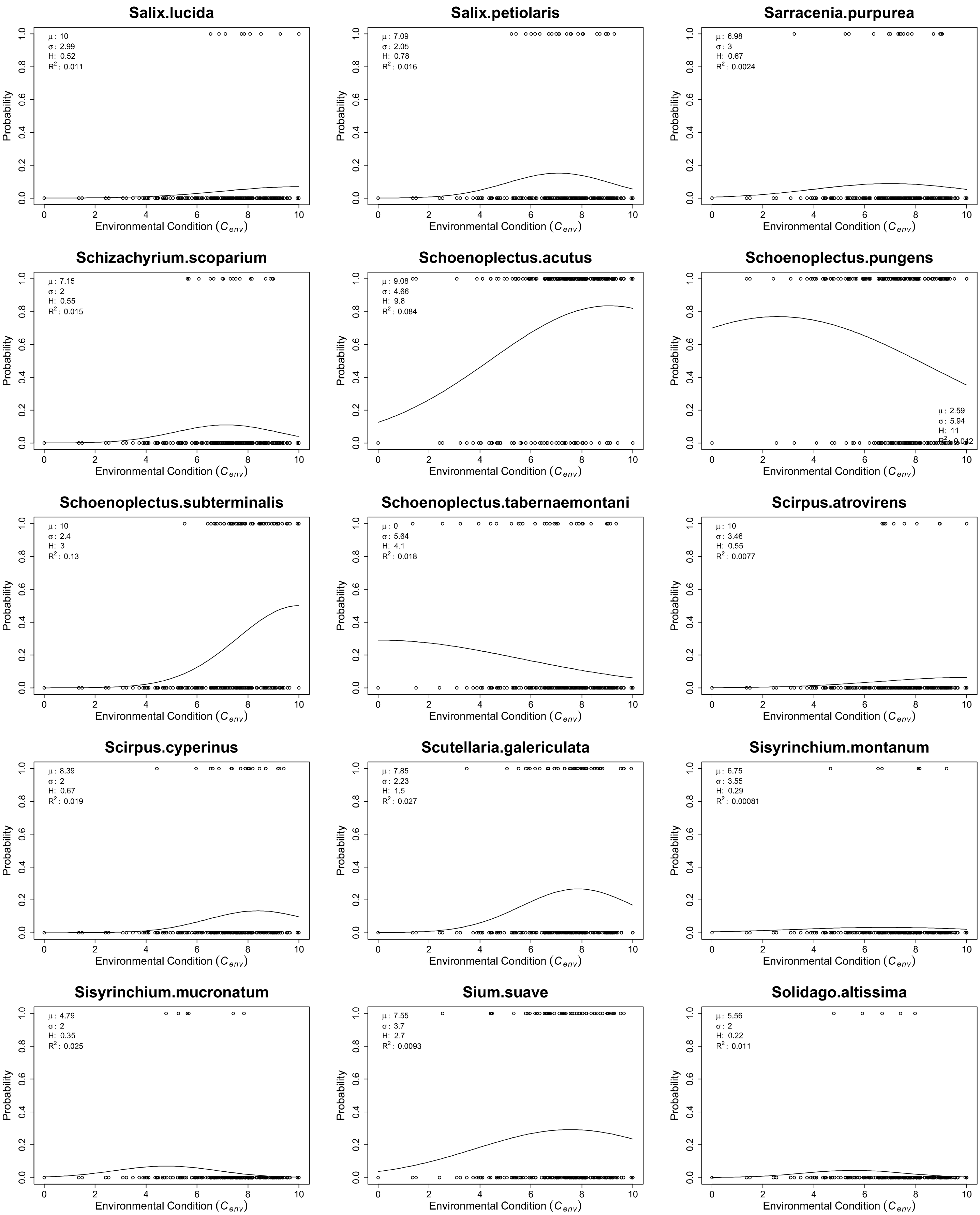
Salix.lucida
μ: 10
σ: 2.99
H: 0.52
R²: 0.011
Salix.petiolaris
μ: 7.09
σ: 2.05
H: 0.78
R²: 0.016
Sarracenia.purpurea
μ: 6.98
σ: 3
H: 0.67
R²: 0.0024
Schizachyrium.scoparium
μ: 7.15
σ: 2
H: 0.55
R²: 0.015
Schoenoplectus.acutus
μ: 9.08
σ: 4.66
H: 9.8
R²: 0.084
Schoenoplectus.pungens
μ: 2.59
σ: 5.94
H: 11
R²: 0.042
Schoenoplectus.subterminalis
μ: 10
σ: 2.4
H: 3
R²: 0.13
Schoenoplectus.tabernaemontani
μ: 0
σ: 5.64
H: 4.1
R²: 0.018
Scirpus.atrovirens
μ: 10
σ: 3.46
H: 0.55
R²: 0.0077
Scirpus.cyperinus
μ: 8.39
σ: 2
H: 0.67
R²: 0.019
Scutellaria.galericulata
μ: 7.85
σ: 2.23
H: 1.5
R²: 0.027
Sisyrinchium.montanum
μ: 6.75
σ: 3.55
H: 0.29
R²: 0.00081
Sisyrinchium.mucronatum
μ: 4.79
σ: 2
H: 0.35
R²: 0.025
Sium.suave
μ: 7.55
σ: 3.7
H: 2.7
R²: 0.0093
Solidago.altissima
μ: 5.56
σ: 2
H: 0.22
R²: 0.011
Probability
Environmental Condition (C_{env})

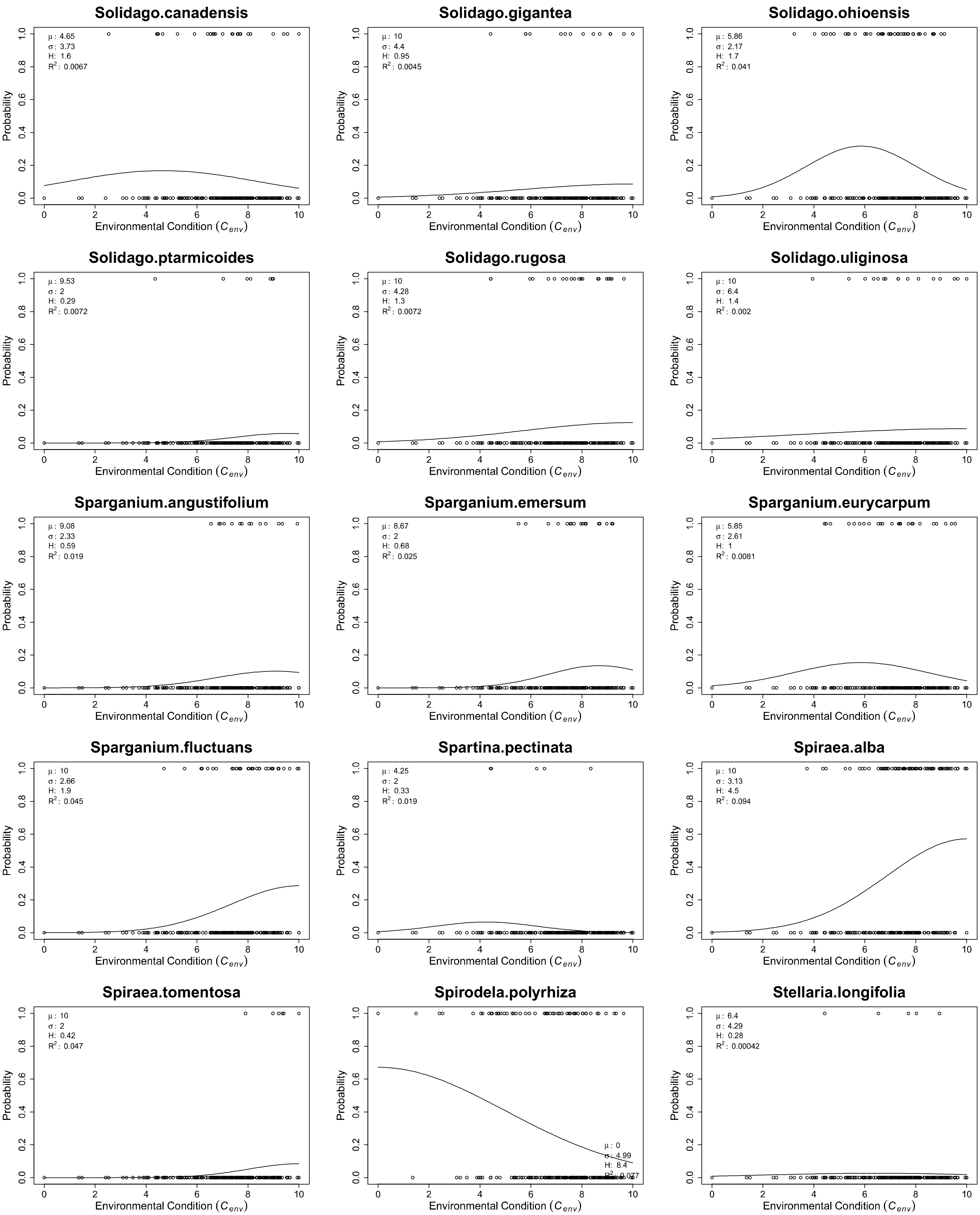
Solidago.canadensis
μ: 4.65
σ: 3.73
H: 1.6
R²: 0.0067
Solidago.gigantea
μ: 10
σ: 4.4
H: 0.95
R²: 0.0045
Solidago.ohioensis
μ: 5.86
σ: 2.17
H: 1.7
R²: 0.041
Solidago.ptarmicoides
μ: 9.53
σ: 2
H: 0.29
R²: 0.0072
Solidago.rugosa
μ: 10
σ: 4.28
H: 1.3
R²: 0.0072
Solidago.uliginosa
μ: 10
σ: 6.4
H: 1.4
R²: 0.002
Sparganium.angustifolium
μ: 9.08
σ: 2.33
H: 0.59
R²: 0.019
Sparganium.emersum
μ: 8.67
σ: 2
H: 0.68
R²: 0.025
Sparganium.eurycarpum
μ: 5.85
σ: 2.61
H: 1
R²: 0.0081
Sparganium.fluctuans
μ: 10
σ: 2.66
H: 1.9
R²: 0.045
Spartina.pectinata
μ: 4.25
σ: 2
H: 0.33
R²: 0.019
Spiraea.alba
μ: 10
σ: 3.13
H: 4.5
R²: 0.094
Spiraea.tomentosa
μ: 10
σ: 2
H: 0.42
R²: 0.047
Spirodela.polyrhiza
μ: 0
σ: 4.99
H: 8.4
Stellaria.longifolia
μ: 6.4
σ: 4.29
H: 0.28
R²: 0.00042
Probability
Environmental Condition (C_{env})

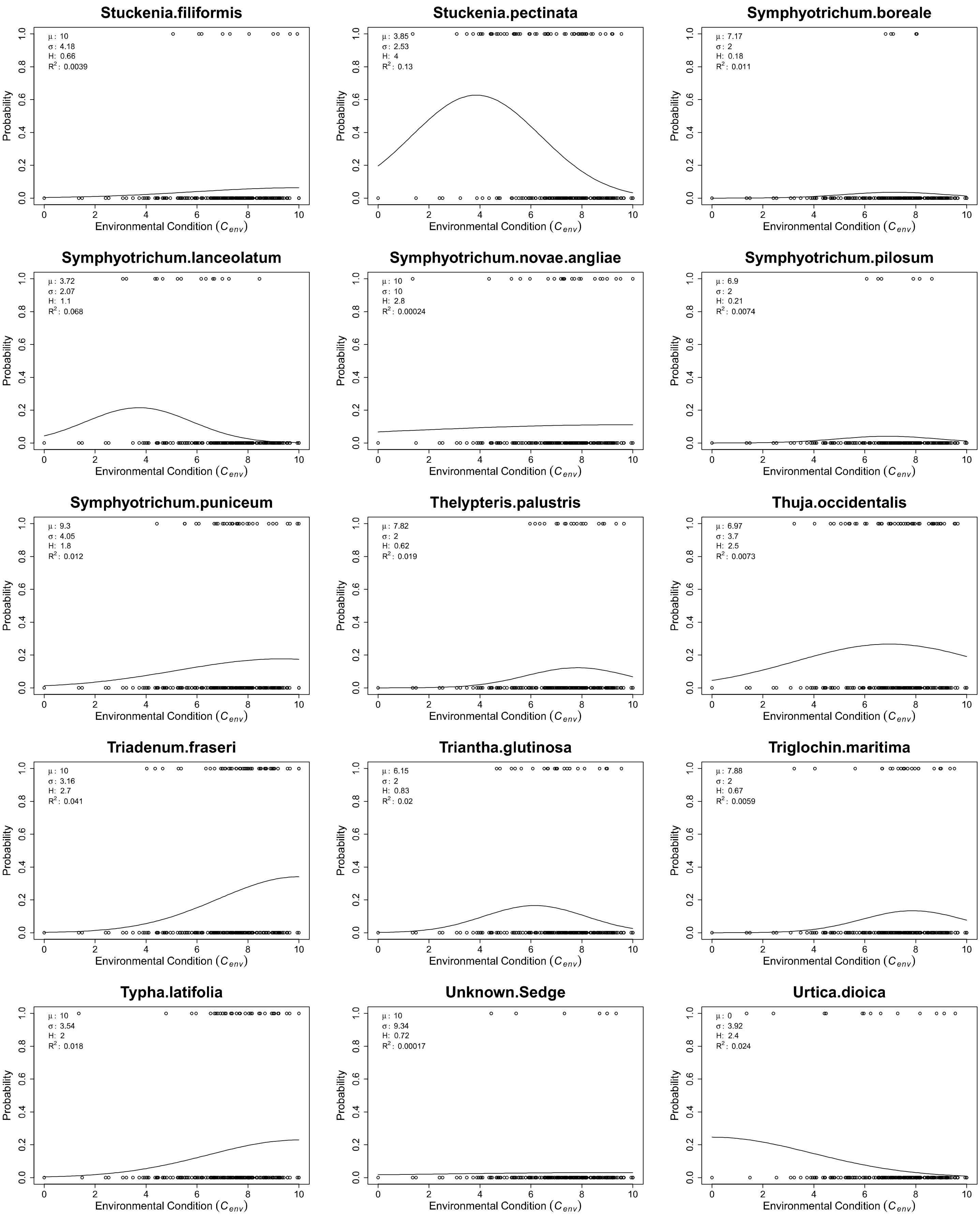

Stuckenia.filiformis
μ: 10
σ: 4.18
H: 0.66
R^2: 0.0039
Stuckenia.pectinata
μ: 3.85
σ: 2.53
H: 4
R^2: 0.13
Symphyotrichum.boreale
μ: 7.17
σ: 2
H: 0.18
R^2: 0.011
Symphyotrichum.lanceolatum
μ: 3.72
σ: 2.07
H: 1.1
R^2: 0.068
Symphyotrichum.novae.angliae
μ: 10
σ: 10
H: 2.8
R^2: 0.00024
Symphyotrichum.pilosum
μ: 6.9
σ: 2
H: 0.21
R^2: 0.0074
Symphyotrichum.puniceum
μ: 9.3
σ: 4.05
H: 1.8
R^2: 0.012
Thelypteris.palustris
μ: 7.82
σ: 2
H: 0.62
R^2: 0.019
Thuja.occidentalis
μ: 6.97
σ: 3.7
H: 2.5
R^2: 0.0073
Triadenum.fraseri
μ: 10
σ: 3.16
H: 2.7
R^2: 0.041
Triantha.glutinosa
μ: 6.15
σ: 2
H: 0.83
R^2: 0.02
Triglochin.maritima
μ: 7.88
σ: 2
H: 0.67
R^2: 0.0059
Typha.latifolia
μ: 10
σ: 3.54
H: 2
R^2: 0.018
Unknown.Sedge
μ: 10
σ: 9.34
H: 0.72
R^2: 0.00017
Urtica.dioica
μ: 0
σ: 3.92
H: 2.4
R^2: 0.024
Probability
Environmental Condition (C_{env})

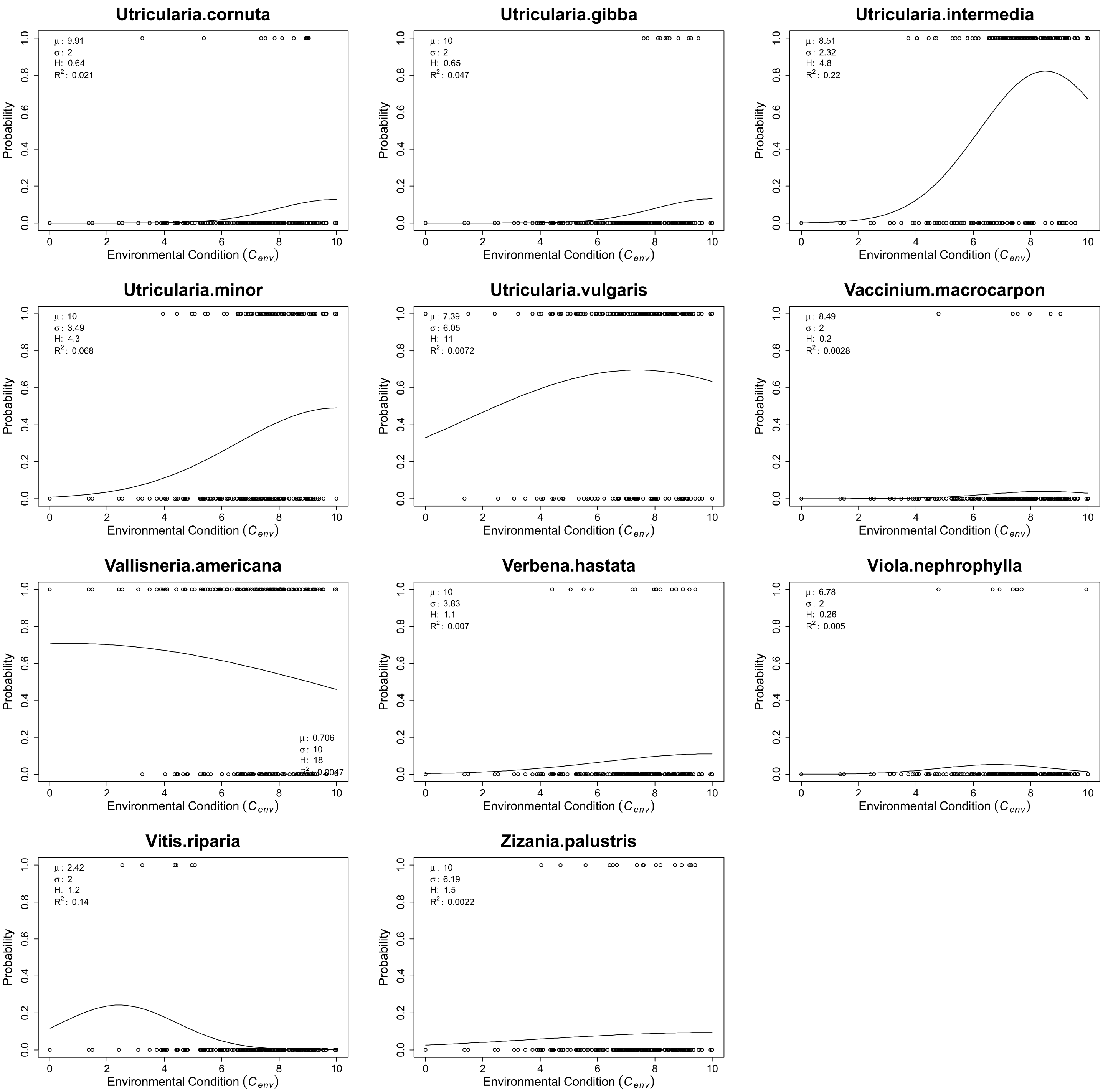

Utricularia.cornuta
μ: 9.91
σ: 2
H: 0.64
R²: 0.021
Utricularia.gibba
μ: 10
σ: 2
H: 0.65
R²: 0.047
Utricularia.intermedia
μ: 8.51
σ: 2.32
H: 4.8
R²: 0.22
Utricularia.minor
μ: 10
σ: 3.49
H: 4.3
R²: 0.068
Utricularia.vulgaris
μ: 7.39
σ: 6.05
H: 11
R²: 0.0072
Vaccinium.macrocarpon
μ: 8.49
σ: 2
H: 0.2
R²: 0.0028
Vallisneria.americana
μ: 0.706
σ: 10
H: 18
R²: 0.0047
Verbena.hastata
μ: 10
σ: 3.83
H: 1.1
R²: 0.007
Viola.nephrophylla
μ: 6.78
σ: 2
H: 0.26
R²: 0.005
Vitis.riparia
μ: 2.42
σ: 2
H: 1.2
R²: 0.14
Zizania.palustris
μ: 10
σ: 6.19
H: 1.5
R²: 0.0022
Probability
Environmental Condition (C_{env})
