# Supplementary material for: Beyond Floristic Quality: A Data-Driven Approach to Assessing Coastal Wetland Condition Using Plants
Source: Wetlands (Wilmington). 2026 Oct 6;46(7):129. doi: 10.1007/s13157-026-02094-z (PMC13642451; doi:10.1007/s13157-026-02094-z)
Supplement: Supplementary file 3 [file 13157_2026_2094_MOESM3_ESM.pdf]

Wetlands

Online Resource 1: Lake Michigan biotic response curves

Beyond floristic quality: A data-driven approach to assessing coastal wetland condition using plants

Giuseppe E. Fiorino[1]*, Simon T. Denomme-Brown[1], Hayley N. Rogers[1], Ian M. Smith[1], Kathryn L. Amatangelo[2], Matthew J. Cooper[3], Nicholas P. Danz[4], Robert W. Howe[5], Rachel E. Schultz[2], Bridget A. Wheelock[6], Douglas A. Wilcox[2], Greg P. Grabas[1]

[1]Environment and Climate Change Canada, 4905 Dufferin Street, Toronto, Ontario M3H 5T4, Canada

[2]Department of Environmental Science and Ecology, SUNY-Brockport, 350 New Campus Drive, Brockport, NY 14420, USA

[3]Department of Biology, Grand Valley State University, Allendale, MI 49401, USA

[4]Department of Natural Sciences, University of Wisconsin-Superior, 1605 Catlin Avenue, Superior, WI 54880, USA

[5]Cofrin Center for Biodiversity, University of Wisconsin-Green Bay, Green Bay, WI 54311-7001, USA

[6]Institute for Great Lakes Research and Department of Biology, Central Michigan University, Mount Pleasant, MI, USA

*Corresponding Author (email: giuseppe.fiorino@ec.gc.ca, ORCID: 0000-0002-1569-0767)

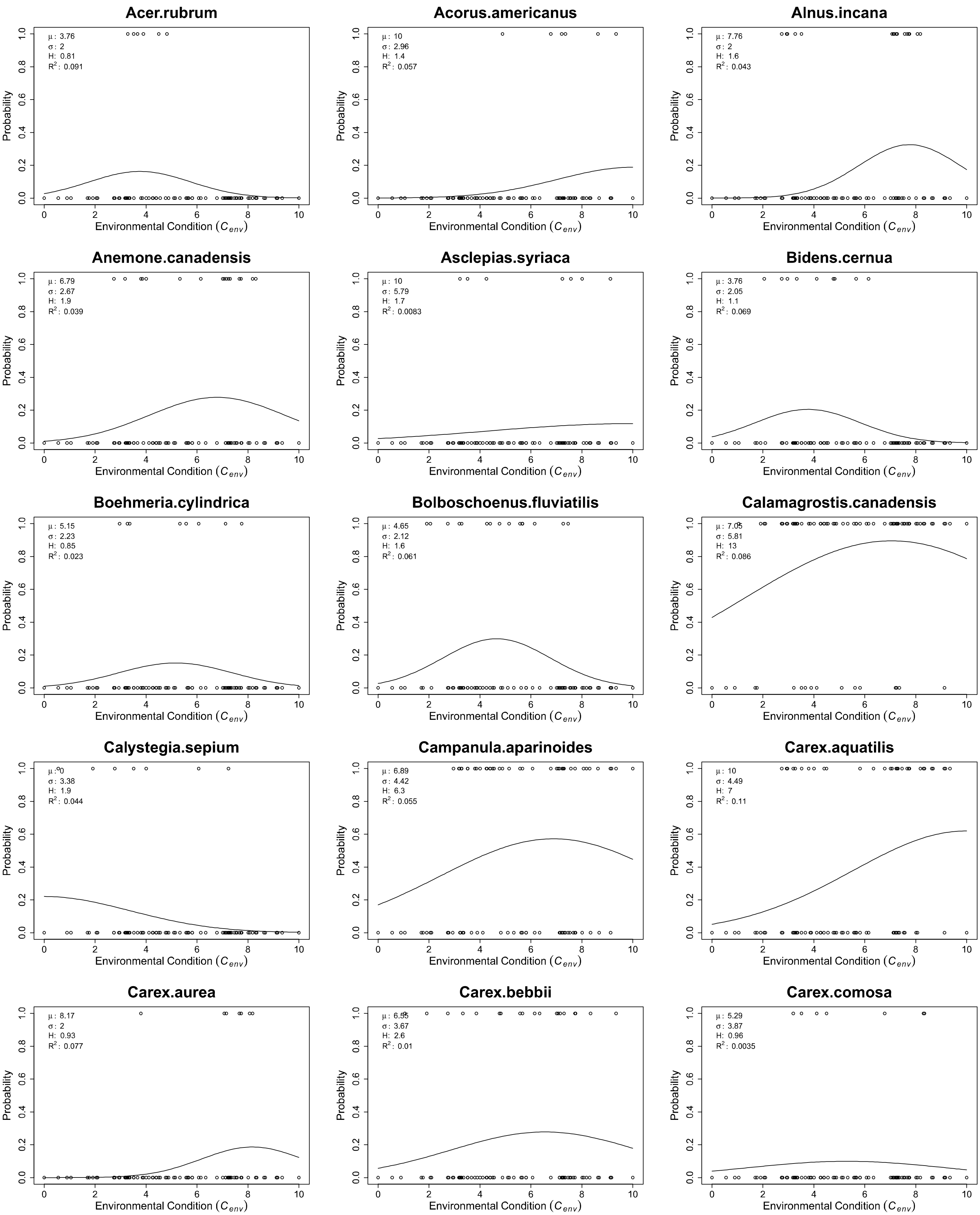
Acer.rubrum
μ: 3.76
σ: 2
H: 0.81
R²: 0.091
Acorus.americanus
μ: 10
σ: 2.96
H: 1.4
R²: 0.057
Alnus.incana
μ: 7.76
σ: 2
H: 1.6
R²: 0.043
Anemone.canadensis
μ: 6.79
σ: 2.67
H: 1.9
R²: 0.039
Asclepias.syriaca
μ: 10
σ: 5.79
H: 1.7
R²: 0.0083
Bidens.cernua
μ: 3.76
σ: 2.05
H: 1.1
R²: 0.069
Boehmeria.cylindrica
μ: 5.15
σ: 2.23
H: 0.85
R²: 0.023
Bolboschoenus.fluviatilis
μ: 4.65
σ: 2.12
H: 1.6
R²: 0.061
Calamagrostis.canadensis
σ: 5.81
H: 13
R²: 0.086
Calystegia.sepium
σ: 3.38
H: 1.9
R²: 0.044
Campanula.aparinoides
μ: 6.89
σ: 4.42
H: 6.3
R²: 0.055
Carex.aquatilis
μ: 10
σ: 4.49
H: 7
R²: 0.11
Carex.aurea
μ: 8.17
σ: 2
H: 0.93
R²: 0.077
Carex.bebbii
σ: 3.67
H: 2.6
R²: 0.01
Carex.comosa
μ: 5.29
σ: 3.87
H: 0.96
R²: 0.0035
Probability
Environmental Condition (C_{env})

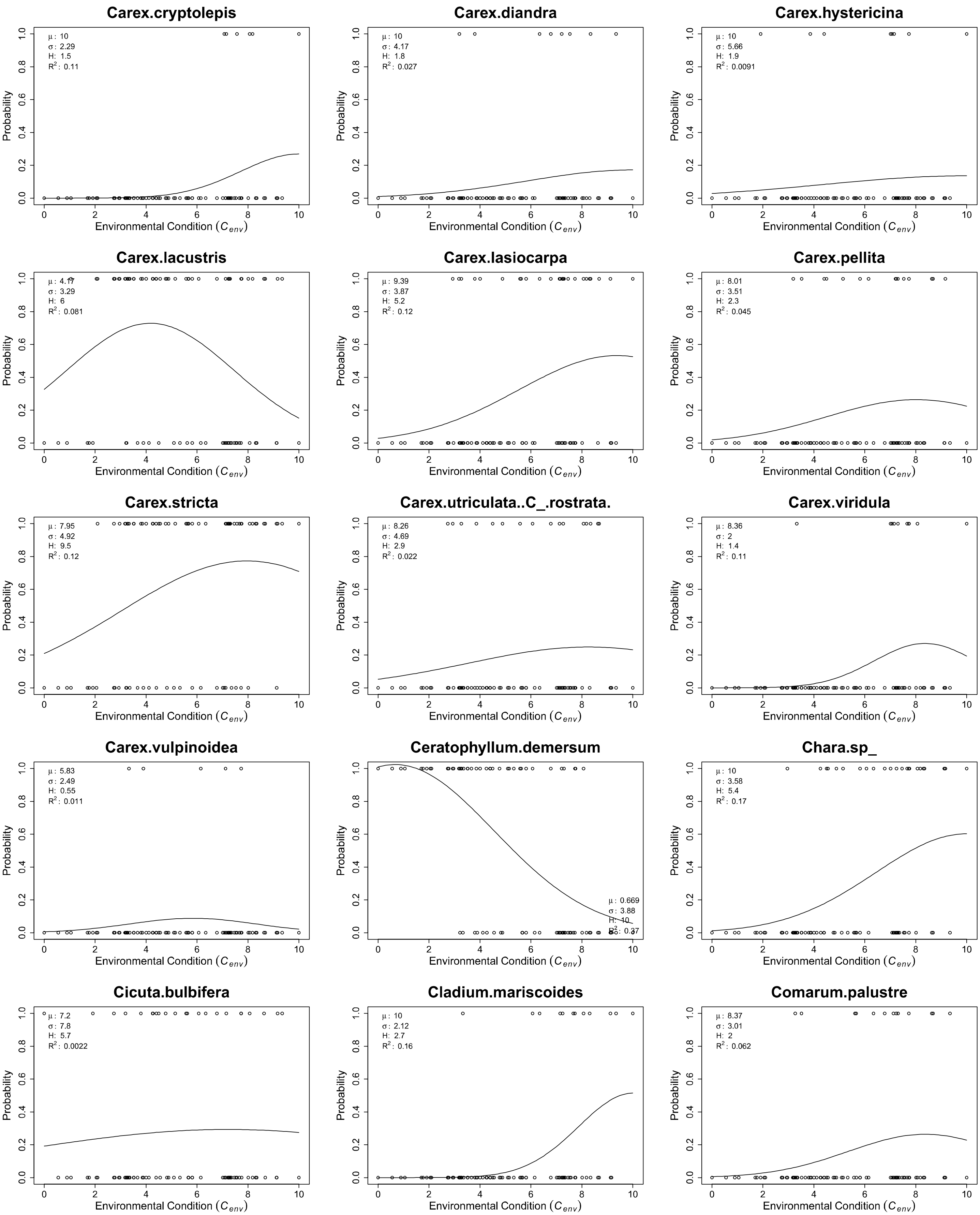

Carex.cryptolepis
μ: 10
σ: 2.29
H: 1.5
R²: 0.11
Carex.diandra
μ: 10
σ: 4.17
H: 1.8
R²: 0.027
Carex.hystericina
μ: 10
σ: 5.66
H: 1.9
R²: 0.0091
Carex.lacustris
μ: 4.17
σ: 3.29
H: 6
R²: 0.081
Carex.lasiocarpa
μ: 9.39
σ: 3.87
H: 5.2
R²: 0.12
Carex.pellita
μ: 8.01
σ: 3.51
H: 2.3
R²: 0.045
Carex.stricta
μ: 7.95
σ: 4.92
H: 9.5
R²: 0.12
Carex.utriculata..C_.rostrata.
μ: 8.26
σ: 4.69
H: 2.9
R²: 0.022
Carex.viridula
μ: 8.36
σ: 2
H: 1.4
R²: 0.11
Carex.vulpinoidea
μ: 5.83
σ: 2.49
H: 0.55
R²: 0.011
Ceratophyllum.demersum
μ: 0.669
σ: 3.88
H: 10
R²: 0.37
Chara.sp_
μ: 10
σ: 3.58
H: 5.4
R²: 0.17
Cicuta.bulbifera
μ: 7.2
σ: 7.8
H: 5.7
R²: 0.0022
Cladium.mariscoides
μ: 10
σ: 2.12
H: 2.7
R²: 0.16
Comarum.palustre
μ: 8.37
σ: 3.01
H: 2
R²: 0.062
Probability
Environmental Condition (C_{env})

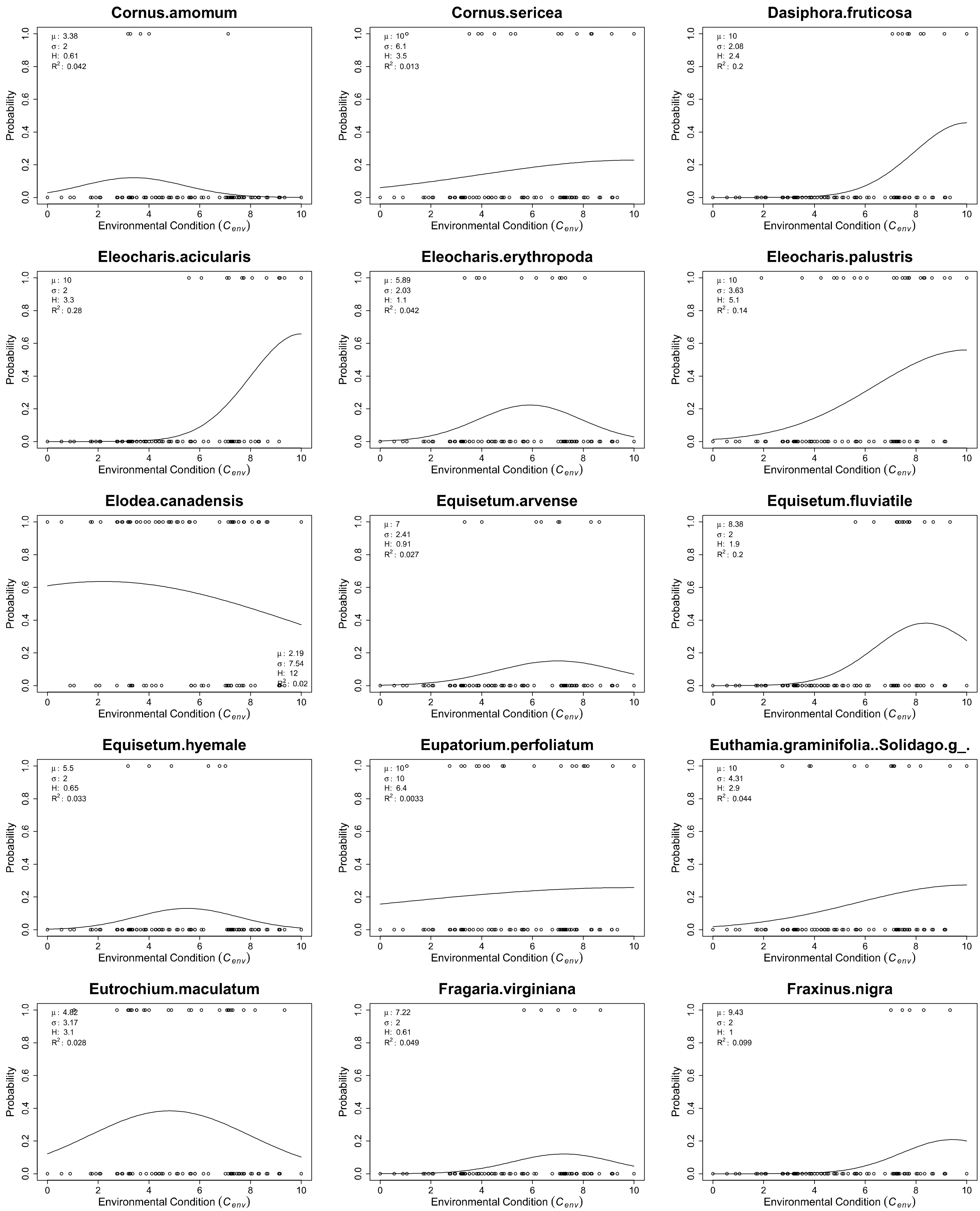
Cornus.amomum
μ: 3.38
σ: 2
H: 0.61
R²: 0.042
Cornus.sericea
μ: 10
σ: 6.1
H: 3.5
R²: 0.013
Dasiphora.fruticosa
μ: 10
σ: 2.08
H: 2.4
R²: 0.2
Eleocharis.acicularis
μ: 10
σ: 2
H: 3.3
R²: 0.28
Eleocharis.erythropoda
μ: 5.89
σ: 2.03
H: 1.1
R²: 0.042
Eleocharis.palustris
μ: 10
σ: 3.63
H: 5.1
R²: 0.14
Elodea.canadensis
μ: 2.19
σ: 7.54
H: 12
R²: 0.02
Equisetum.arvense
μ: 7
σ: 2.41
H: 0.91
R²: 0.027
Equisetum.fluviatile
μ: 8.38
σ: 2
H: 1.9
R²: 0.2
Equisetum.hyemale
μ: 5.5
σ: 2
H: 0.65
R²: 0.033
Eupatorium.perfoliatum
μ: 10
σ: 10
H: 6.4
R²: 0.0033
Euthamia.graminifolia..Solidago.g_.
μ: 10
σ: 4.31
H: 2.9
R²: 0.044
Eutrochium.maculatum
μ: 4.82
σ: 3.17
H: 3.1
R²: 0.028
Fragaria.virginiana
μ: 7.22
σ: 2
H: 0.61
R²: 0.049
Fraxinus.nigra
μ: 9.43
σ: 2
H: 1
R²: 0.099
Probability
Environmental Condition (C_{env})

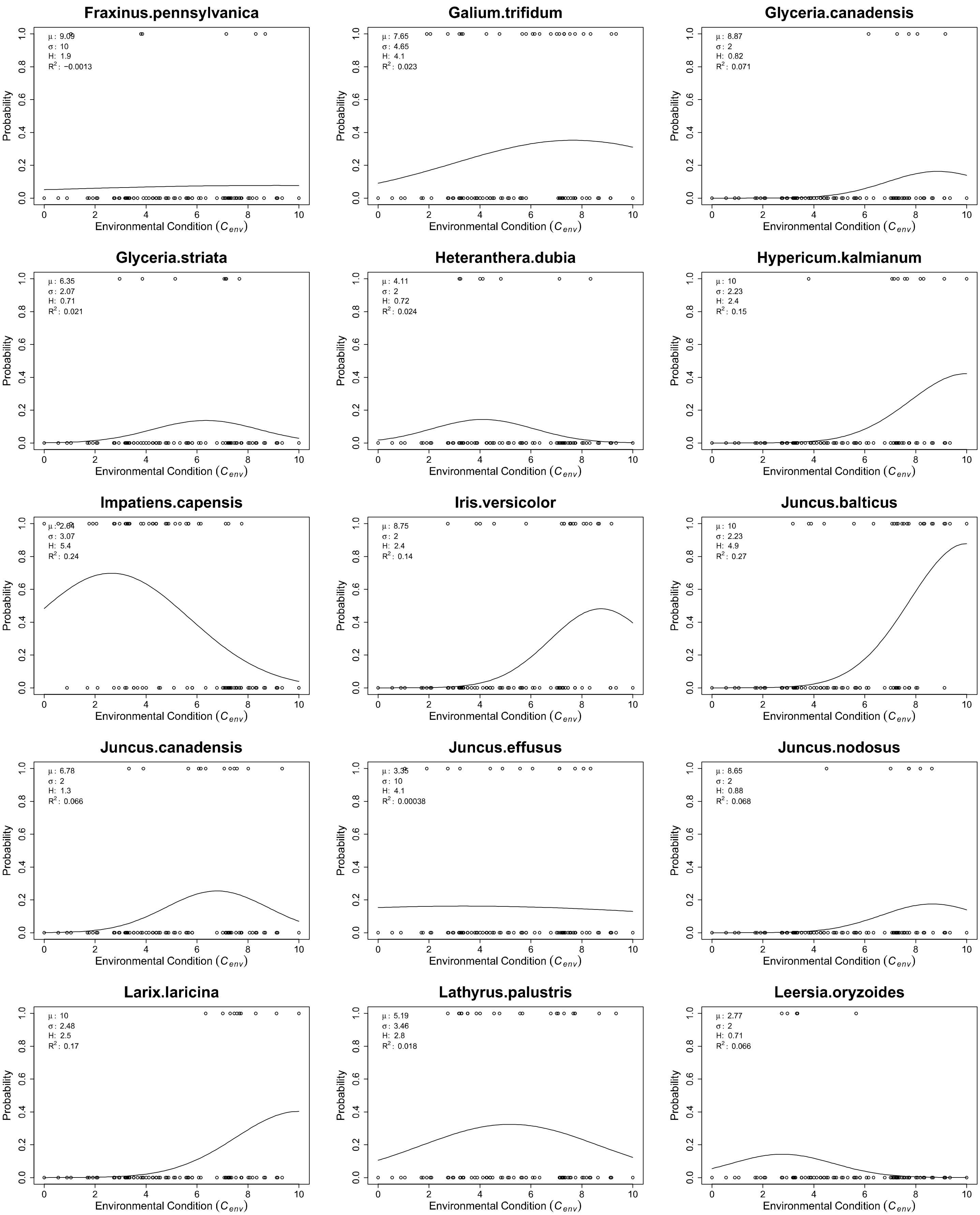
Fraxinus.pennsylvanica
μ: 9.09
σ: 10
H: 1.9
R²: −0.0013
Galium.trifidum
μ: 7.65
σ: 4.65
H: 4.1
R²: 0.023
Glyceria.canadensis
μ: 8.87
σ: 2
H: 0.82
R²: 0.071
Glyceria.striata
μ: 6.35
σ: 2.07
H: 0.71
R²: 0.021
Heteranthera.dubia
μ: 4.11
σ: 2
H: 0.72
R²: 0.024
Hypericum.kalmianum
μ: 10
σ: 2.23
H: 2.4
R²: 0.15
Impatiens.capensis
μ: 2.64
σ: 3.07
H: 5.4
R²: 0.24
Iris.versicolor
μ: 8.75
σ: 2
H: 2.4
R²: 0.14
Juncus.balticus
μ: 10
σ: 2.23
H: 4.9
R²: 0.27
Juncus.canadensis
μ: 6.78
σ: 2
H: 1.3
R²: 0.066
Juncus.effusus
μ: 3.35
σ: 10
H: 4.1
R²: 0.00038
Juncus.nodosus
μ: 8.65
σ: 2
H: 0.88
R²: 0.068
Larix.laricina
μ: 10
σ: 2.48
H: 2.5
R²: 0.17
Lathyrus.palustris
μ: 5.19
σ: 3.46
H: 2.8
R²: 0.018
Leersia.oryzoides
μ: 2.77
σ: 2
H: 0.71
R²: 0.066
Probability
Environmental Condition (C_{env})

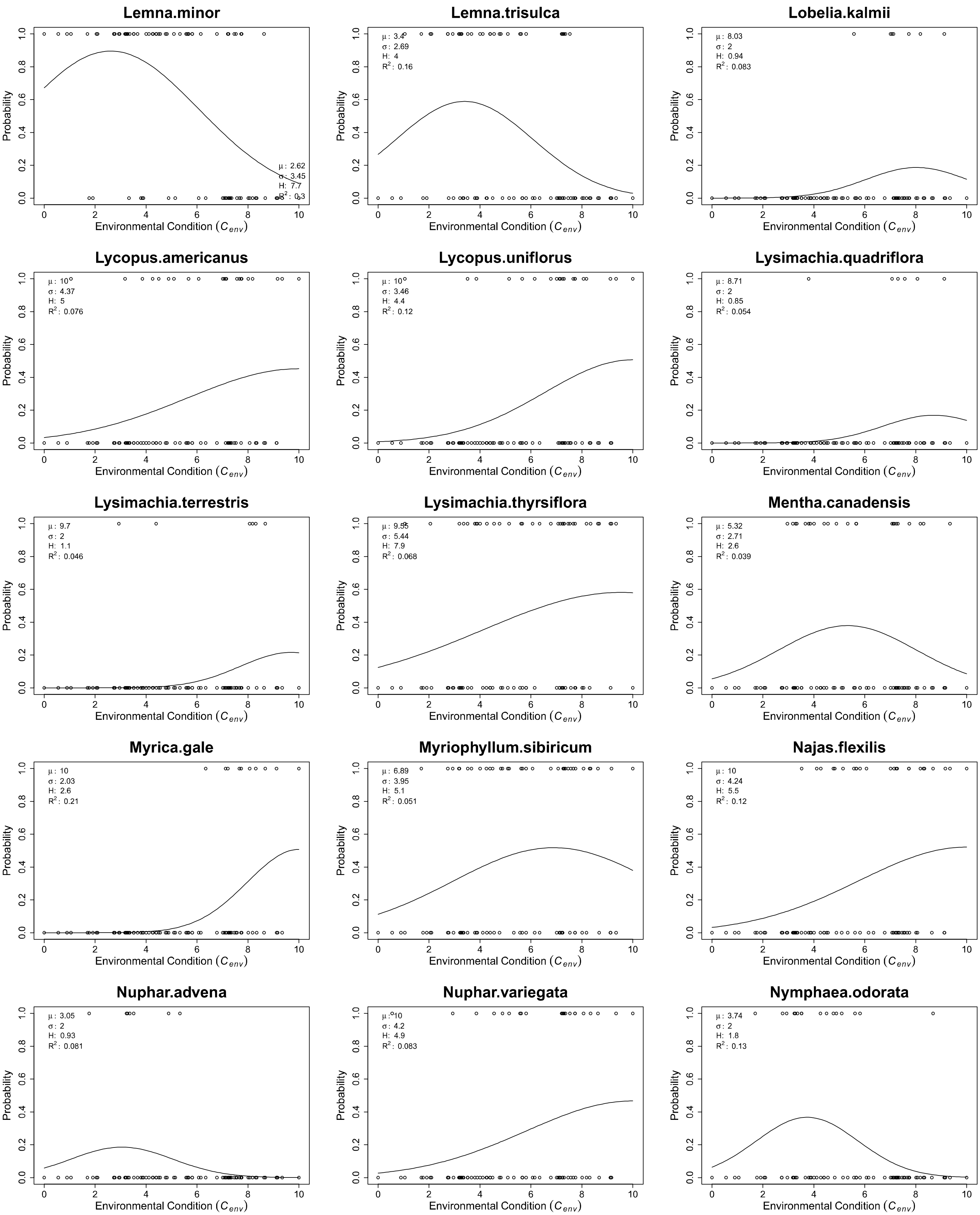
Lemna.minor
μ: 2.62
σ: 3.45
H: 7.7
R²: 0.3
Lemna.trisulca
μ: 3.4
σ: 2.69
H: 4
R²: 0.16
Lobelia.kalmii
μ: 8.03
σ: 2
H: 0.94
R²: 0.083
Lycopus.americanus
μ: 10
σ: 4.37
H: 5
R²: 0.076
Lycopus.uniflorus
μ: 10
σ: 3.46
H: 4.4
R²: 0.12
Lysimachia.quadriflora
μ: 8.71
σ: 2
H: 0.85
R²: 0.054
Lysimachia.terrestris
μ: 9.7
σ: 2
H: 1.1
R²: 0.046
Lysimachia.thyrsiflora
μ: 9.55
σ: 5.44
H: 7.9
R²: 0.068
Mentha.canadensis
μ: 5.32
σ: 2.71
H: 2.6
R²: 0.039
Myrica.gale
μ: 10
σ: 2.03
H: 2.6
R²: 0.21
Myriophyllum.sibiricum
μ: 6.89
σ: 3.95
H: 5.1
R²: 0.051
Najas.flexilis
μ: 10
σ: 4.24
H: 5.5
R²: 0.12
Nuphar.advena
μ: 3.05
σ: 2
H: 0.93
R²: 0.081
Nuphar.variegata
μ: 10
σ: 4.2
H: 4.9
R²: 0.083
Nymphaea.odorata
μ: 3.74
σ: 2
H: 1.8
R²: 0.13
Probability
Environmental Condition (C_{env})

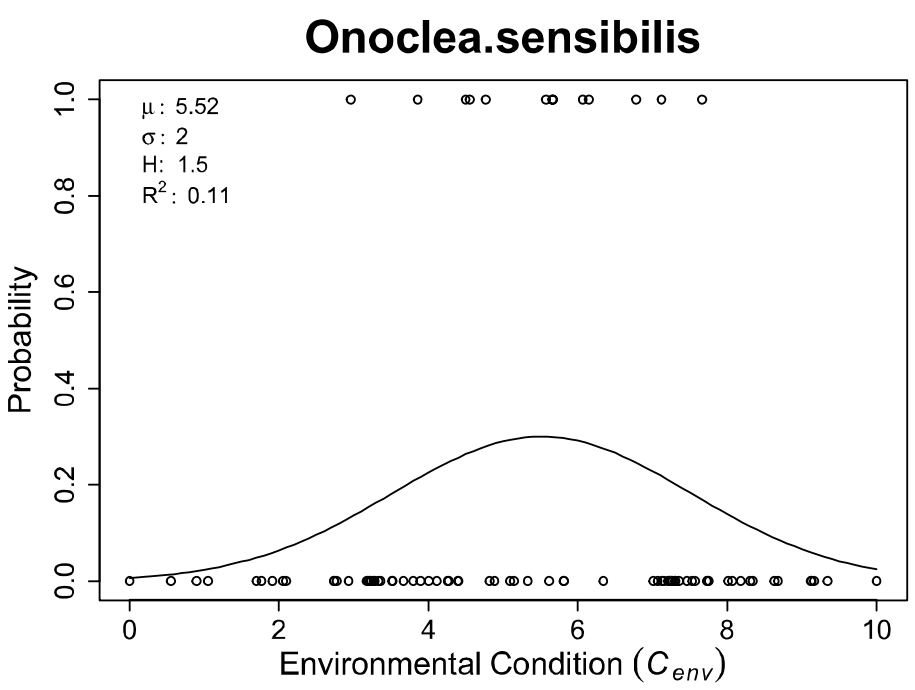
Onoclea.sensibilis
μ: 5.52
σ: 2
H: 1.5
R2: 0.11
Probability
Environmental Condition (C_{env})

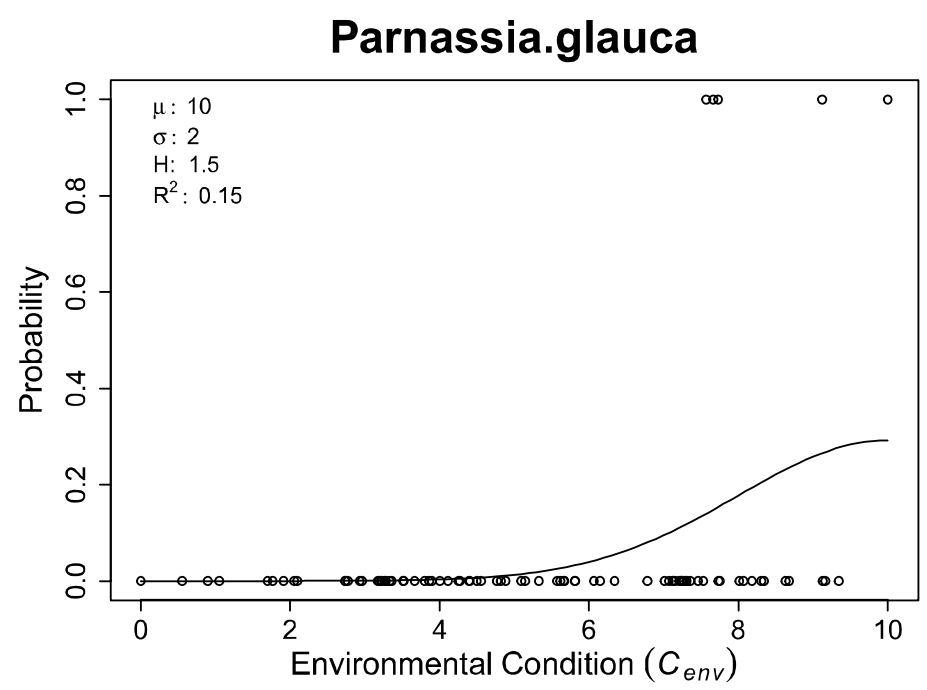
Parnassia.glauca
μ: 10
σ: 2
H: 1.5
R2: 0.15
Probability
Environmental Condition (C_{env})

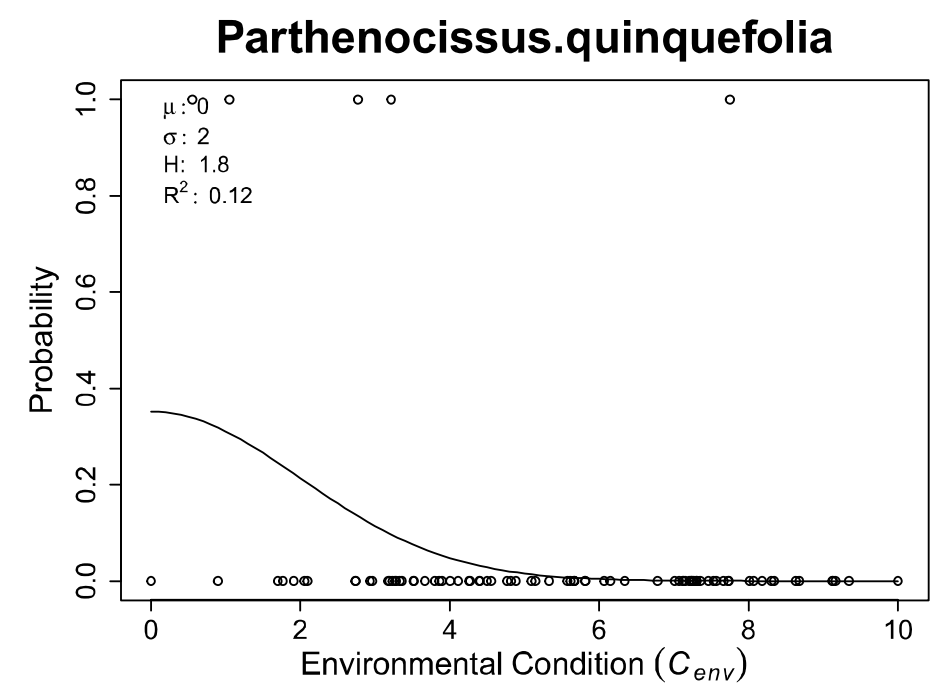
Parthenocissus.quinquefolia
μ: 0
σ: 2
H: 1.8
R2: 0.12
Probability
Environmental Condition (C_{env})

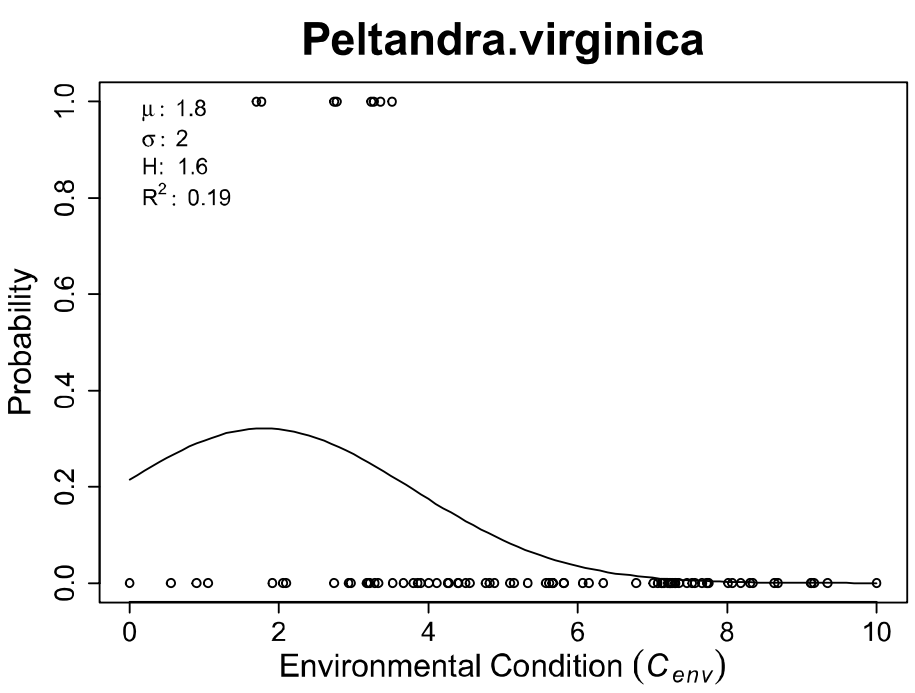
Peltandra.virginica
μ: 1.8
σ: 2
H: 1.6
R2: 0.19
Probability
Environmental Condition (C_{env})

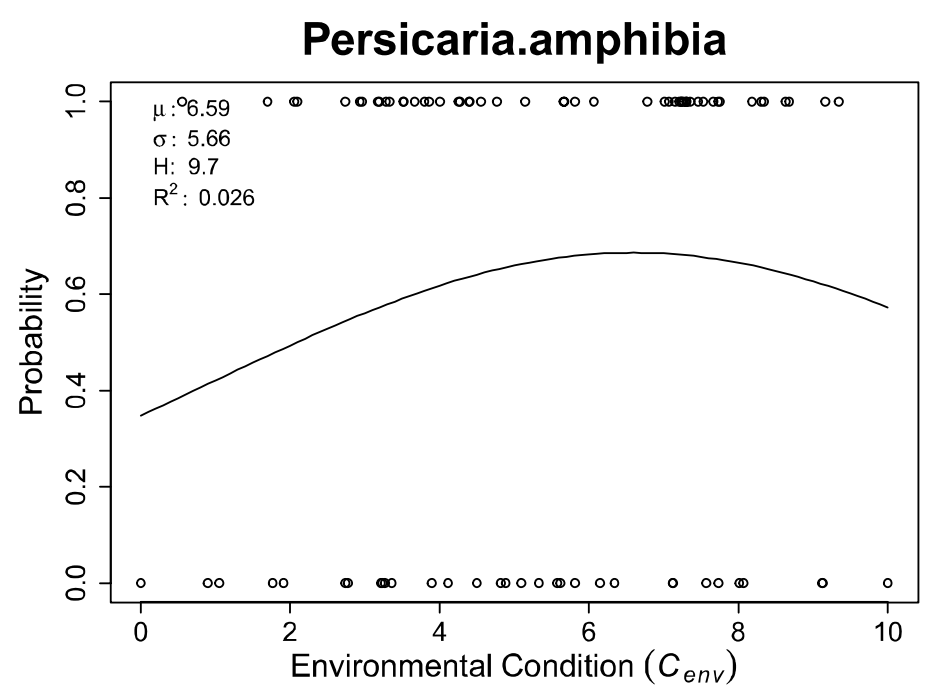
Persicaria.amphibia
μ: 6.59
σ: 5.66
H: 9.7
R2: 0.026
Probability
Environmental Condition (C_{env})

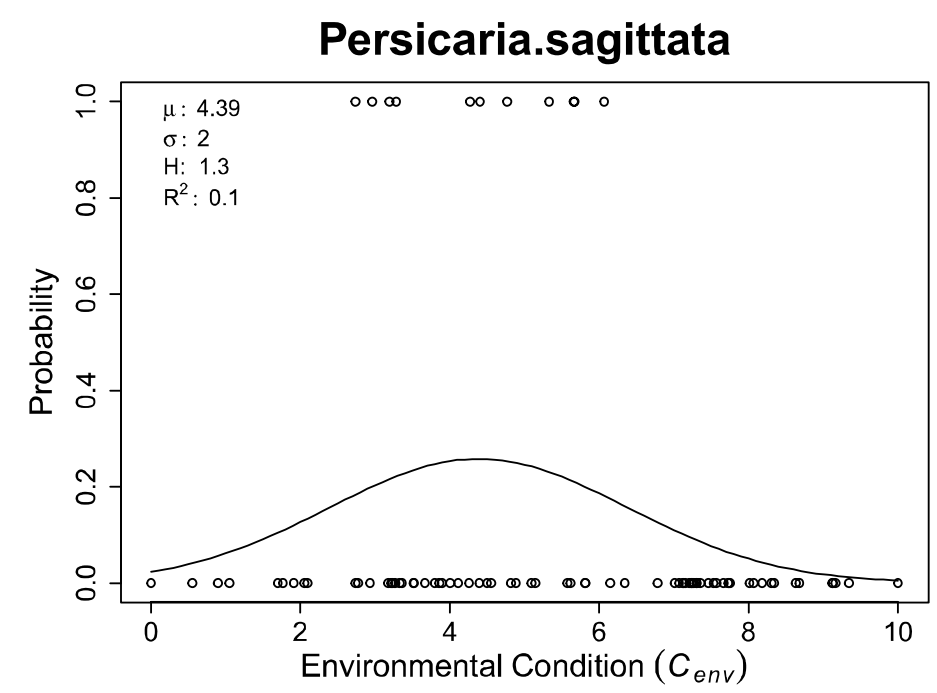
Persicaria.sagittata
μ: 4.39
σ: 2
H: 1.3
R2: 0.1
Probability
Environmental Condition (C_{env})

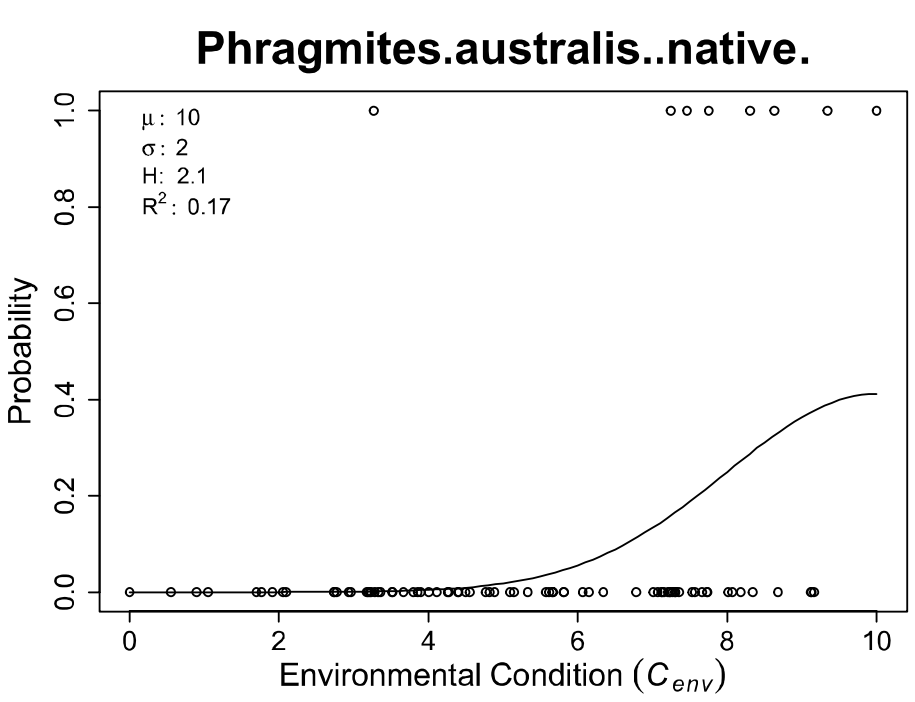
Phragmites.australis..native.
μ: 10
σ: 2
H: 2.1
R2: 0.17
Probability
Environmental Condition (C_{env})

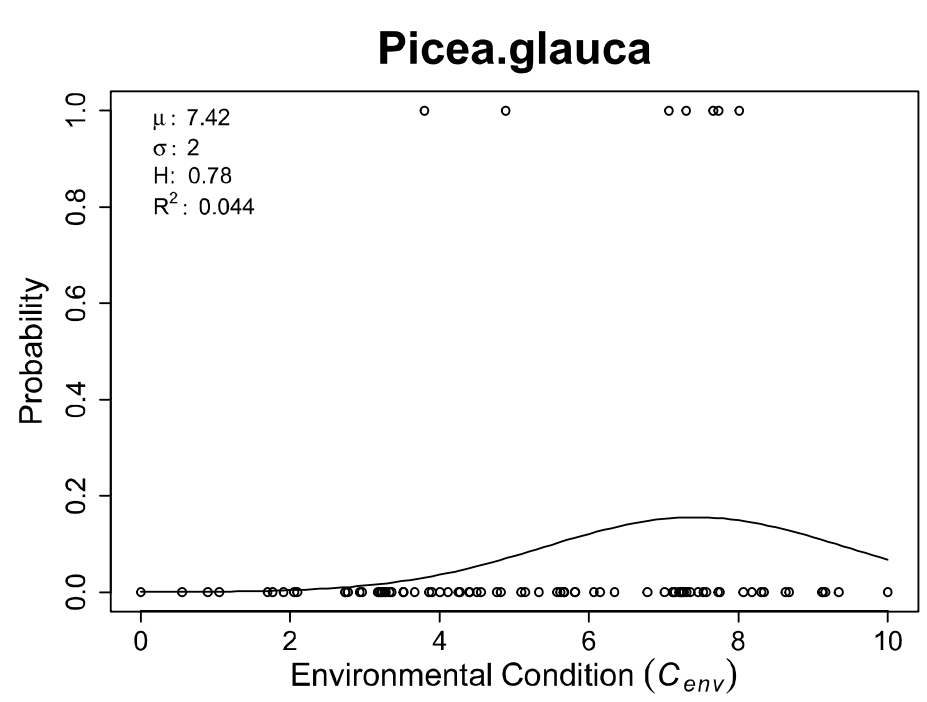
Picea.glauca
μ: 7.42
σ: 2
H: 0.78
R2: 0.044
Probability
Environmental Condition (C_{env})

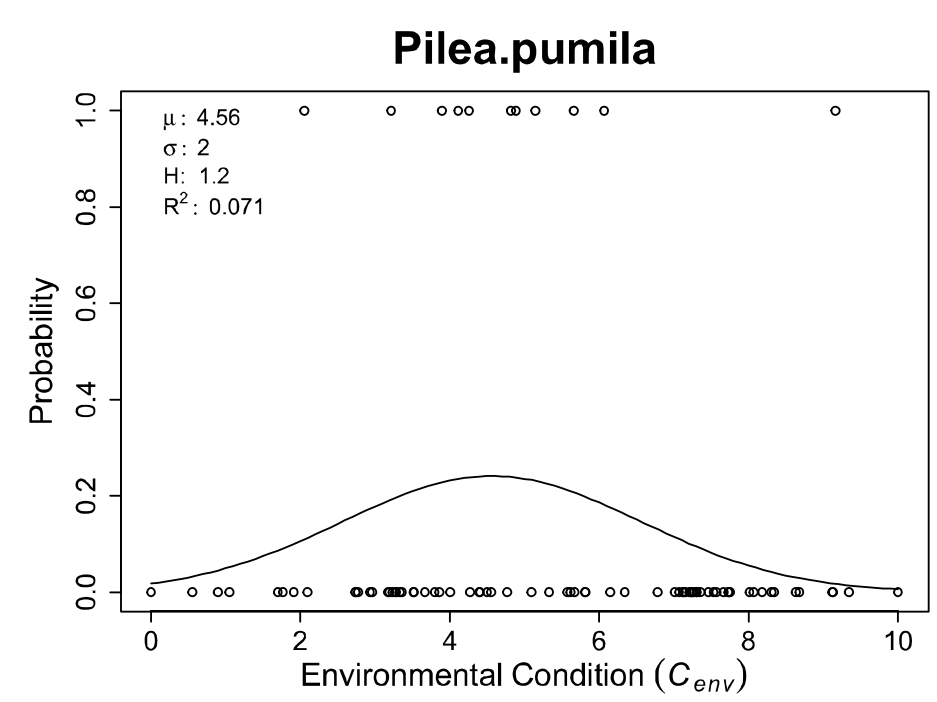
Pilea.pumila
μ: 4.56
σ: 2
H: 1.2
R2: 0.071
Probability
Environmental Condition (C_{env})

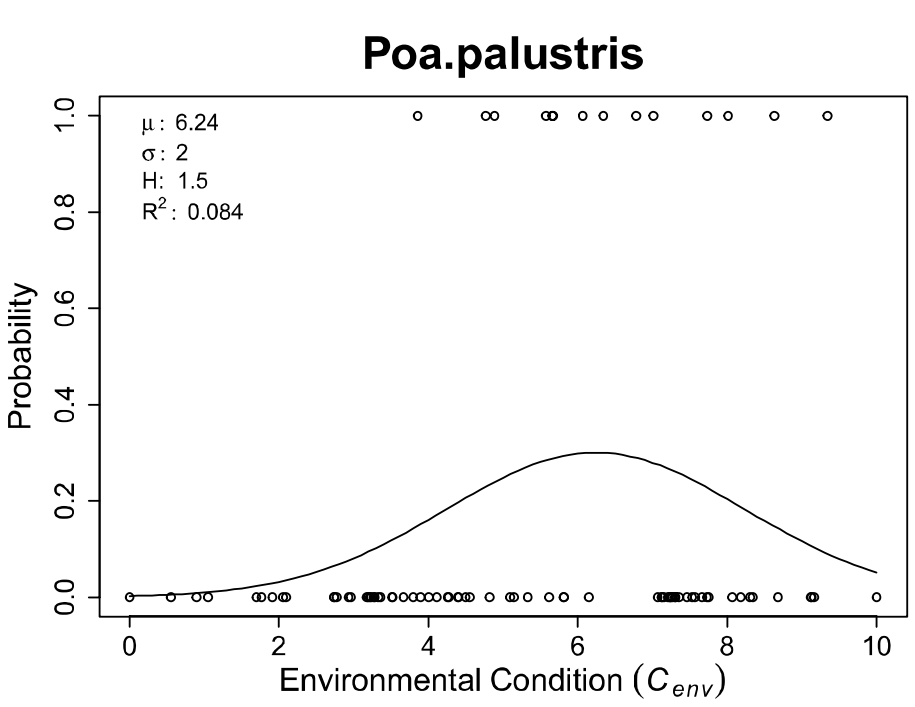
Poa.palustris
μ: 6.24
σ: 2
H: 1.5
R2: 0.084
Probability
Environmental Condition (C_{env})

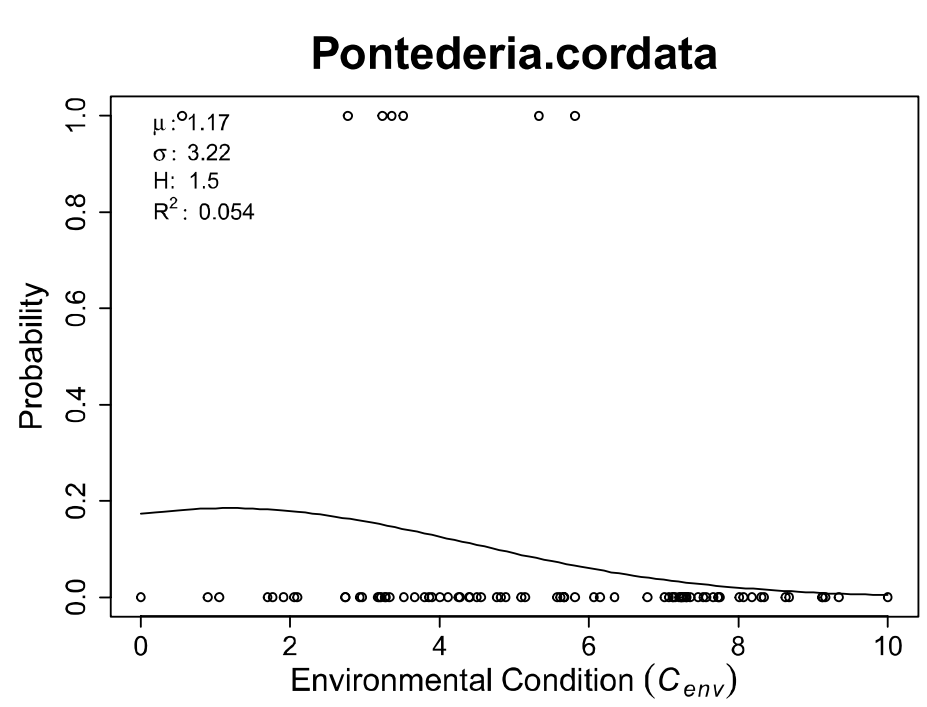
Pontederia.cordata
μ: 1.17
σ: 3.22
H: 1.5
R2: 0.054
Probability
Environmental Condition (C_{env})

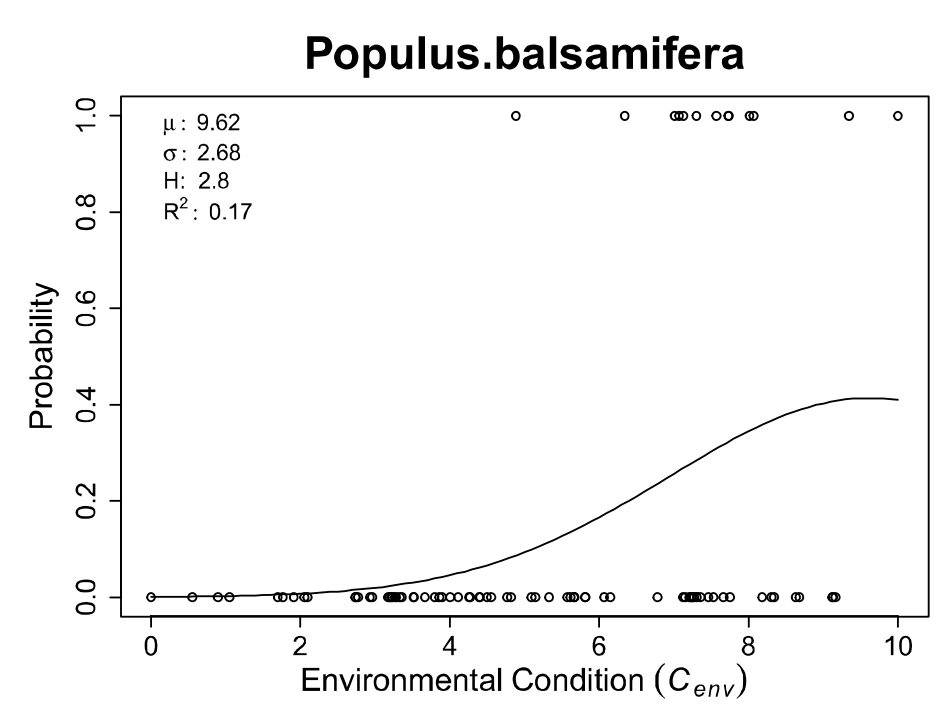
Populus.balsamifera
μ: 9.62
σ: 2.68
H: 2.8
R2: 0.17
Probability
Environmental Condition (C_{env})

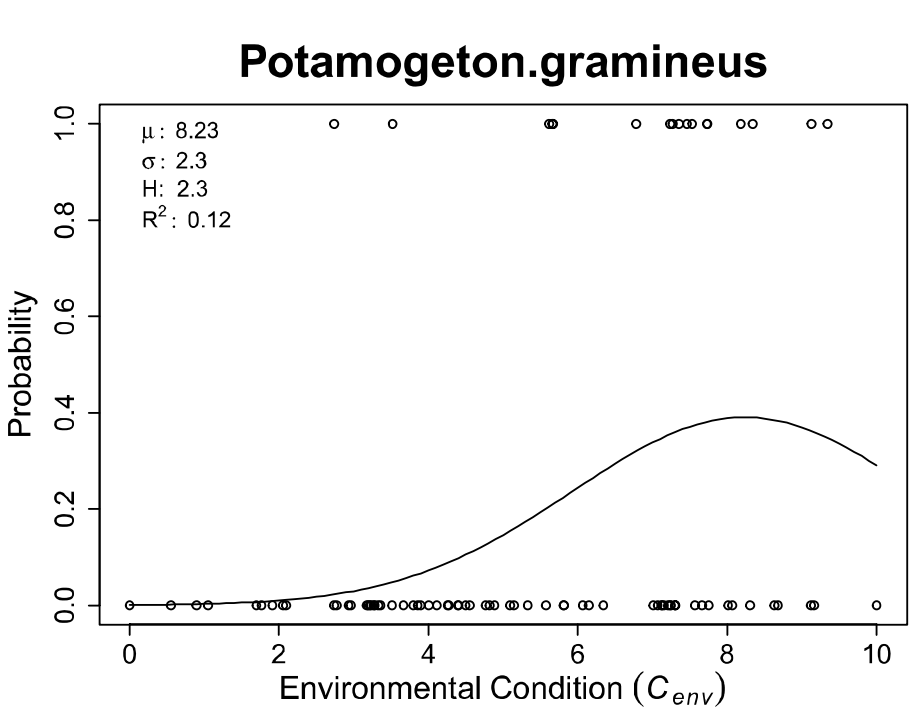
Potamogeton.gramineus
μ: 8.23
σ: 2.3
H: 2.3
R2: 0.12
Probability
Environmental Condition (C_{env})

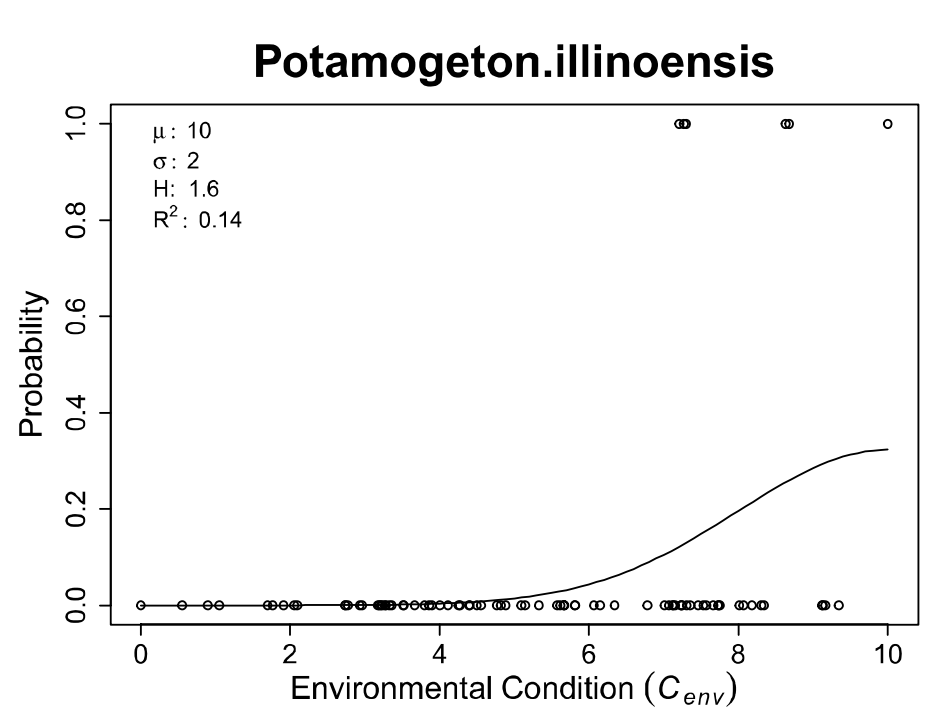
Potamogeton.illinoensis
μ: 10
σ: 2
H: 1.6
R2: 0.14
Probability
Environmental Condition (C_{env})

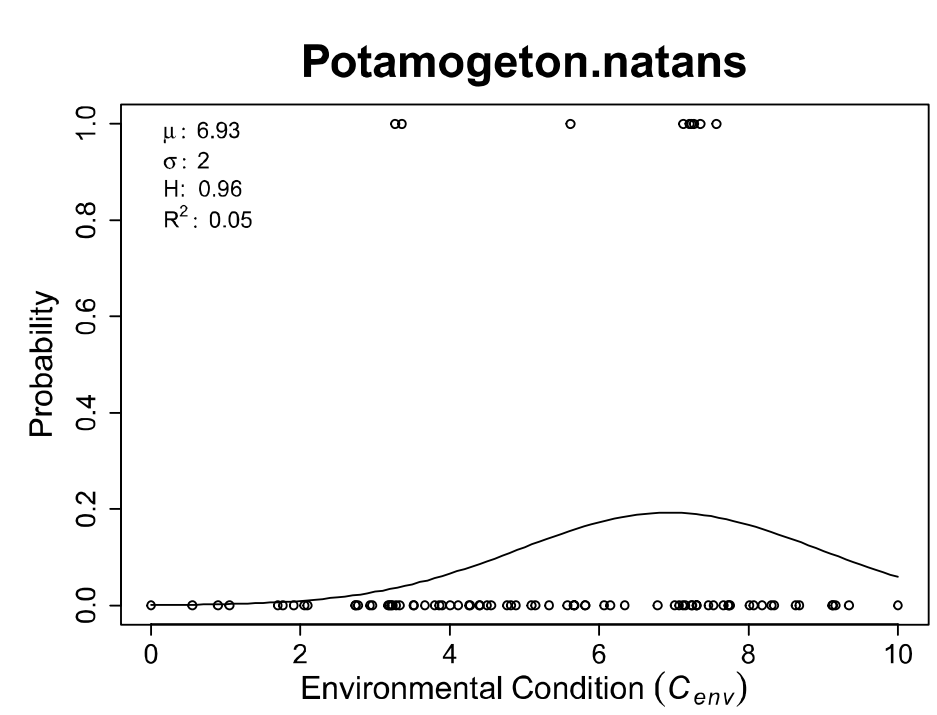
Potamogeton.natans
μ: 6.93
σ: 2
H: 0.96
R2: 0.05
Probability
Environmental Condition (C_{env})

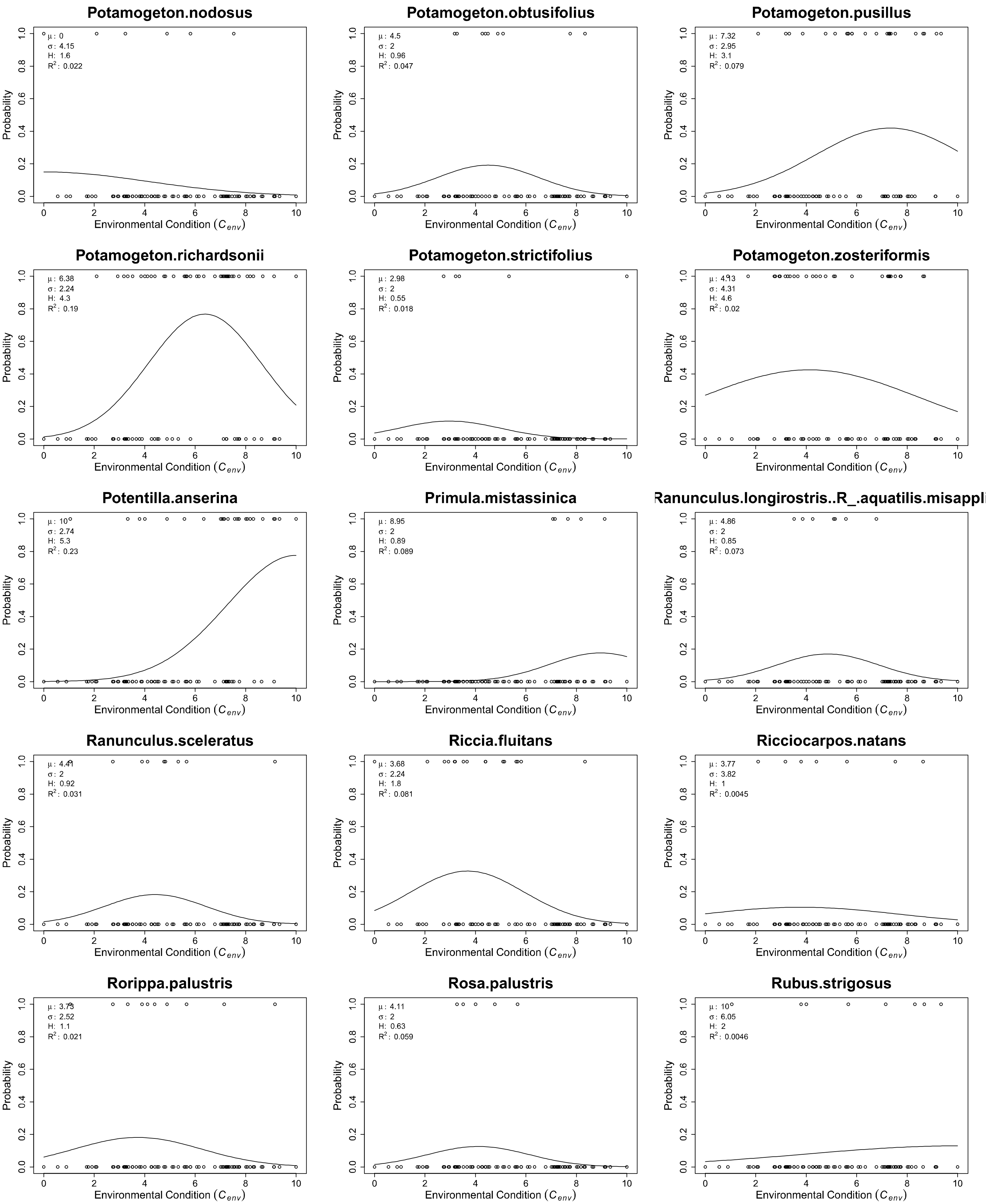
Potamogeton.nodosus
μ: 0
σ: 4.15
H: 1.6
R²: 0.022
Potamogeton.obtusifolius
μ: 4.5
σ: 2
H: 0.96
R²: 0.047
Potamogeton.pusillus
μ: 7.32
σ: 2.95
H: 3.1
R²: 0.079
Potamogeton.richardsonii
μ: 6.38
σ: 2.24
H: 4.3
R²: 0.19
Potamogeton.strictifolius
μ: 2.98
σ: 2
H: 0.55
R²: 0.018
Potamogeton.zosteriformis
μ: 4.13
σ: 4.31
H: 4.6
R²: 0.02
Potentilla.anserina
μ: 10
σ: 2.74
H: 5.3
R²: 0.23
Primula.mistassinica
μ: 8.95
σ: 2
H: 0.89
R²: 0.089
Ranunculus.longirostris..R_.aquatilis.misappli
μ: 4.86
σ: 2
H: 0.85
R²: 0.073
Ranunculus.sceleratus
μ: 4.41
σ: 2
H: 0.92
R²: 0.031
Riccia.fluitans
μ: 3.68
σ: 2.24
H: 1.8
R²: 0.081
Ricciocarpos.natans
μ: 3.77
σ: 3.82
H: 1
R²: 0.0045
Rorippa.palustris
μ: 3.73
σ: 2.52
H: 1.1
R²: 0.021
Rosa.palustris
μ: 4.11
σ: 2
H: 0.63
R²: 0.059
Rubus.strigosus
μ: 10
σ: 6.05
H: 2
R²: 0.0046
Probability
Environmental Condition (C_{env})

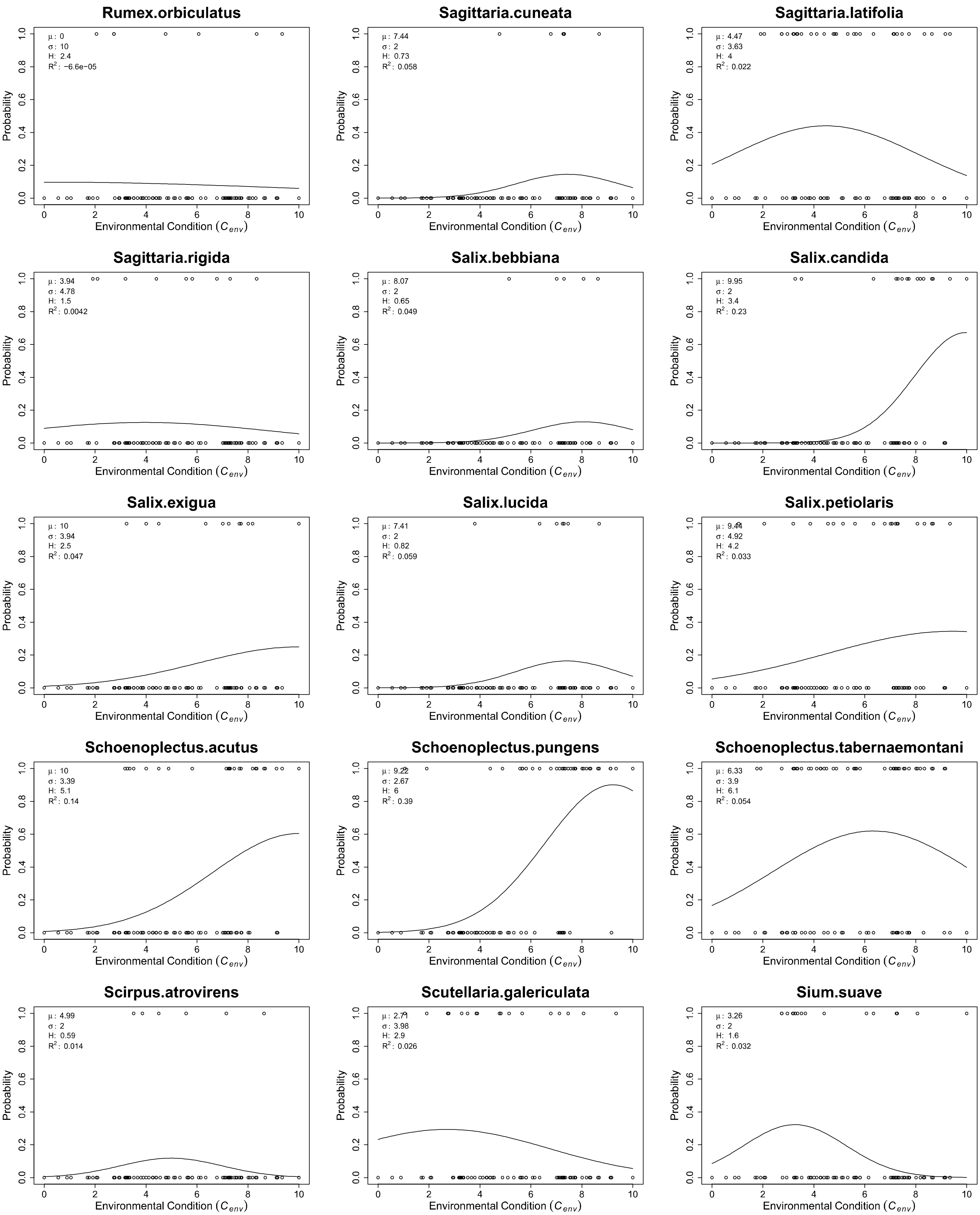

Rumex.orbiculatus
μ: 0
σ: 10
H: 2.4
R^2: −6.6e−05
Sagittaria.cuneata
μ: 7.44
σ: 2
H: 0.73
R^2: 0.058
Sagittaria.latifolia
μ: 4.47
σ: 3.63
H: 4
R^2: 0.022
Sagittaria.rigida
μ: 3.94
σ: 4.78
H: 1.5
R^2: 0.0042
Salix.bebbiana
μ: 8.07
σ: 2
H: 0.65
R^2: 0.049
Salix.candida
μ: 9.95
σ: 2
H: 3.4
R^2: 0.23
Salix.exigua
μ: 10
σ: 3.94
H: 2.5
R^2: 0.047
Salix.lucida
μ: 7.41
σ: 2
H: 0.82
R^2: 0.059
Salix.petiolaris
μ: 9.44
σ: 4.92
H: 4.2
R^2: 0.033
Schoenoplectus.acutus
μ: 10
σ: 3.39
H: 5.1
R^2: 0.14
Schoenoplectus.pungens
μ: 9.22
σ: 2.67
H: 6
R^2: 0.39
Schoenoplectus.tabernaemontani
μ: 6.33
σ: 3.9
H: 6.1
R^2: 0.054
Scirpus.atrovirens
μ: 4.99
σ: 2
H: 0.59
R^2: 0.014
Scutellaria.galericulata
μ: 2.71
σ: 3.98
H: 2.9
R^2: 0.026
Sium.suave
μ: 3.26
σ: 2
H: 1.6
R^2: 0.032
Probability
Environmental Condition (C_{env})

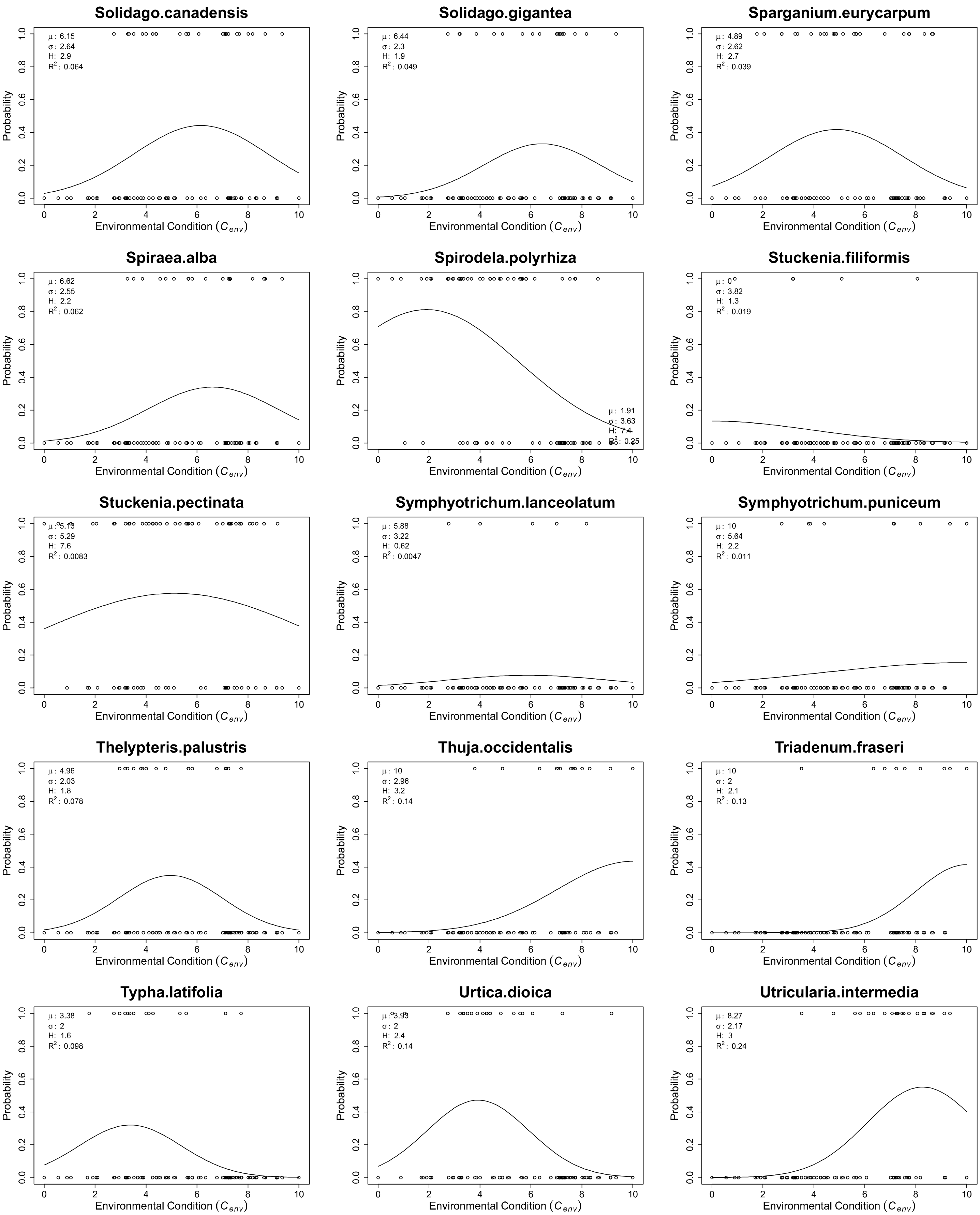
Solidago.canadensis
μ: 6.15
σ: 2.64
H: 2.9
R²: 0.064
Solidago.gigantea
μ: 6.44
σ: 2.3
H: 1.9
R²: 0.049
Sparganium.eurycarpum
μ: 4.89
σ: 2.62
H: 2.7
R²: 0.039
Spiraea.alba
μ: 6.62
σ: 2.55
H: 2.2
R²: 0.062
Spirodela.polyrhiza
μ: 1.91
σ: 3.63
H: 7.4
R²: 0.25
Stuckenia.filiformis
μ: 0
σ: 3.82
H: 1.3
R²: 0.019
Stuckenia.pectinata
μ: 5.13
σ: 5.29
H: 7.6
R²: 0.0083
Symphyotrichum.lanceolatum
μ: 5.88
σ: 3.22
H: 0.62
R²: 0.0047
Symphyotrichum.puniceum
μ: 10
σ: 5.64
H: 2.2
R²: 0.011
Thelypteris.palustris
μ: 4.96
σ: 2.03
H: 1.8
R²: 0.078
Thuja.occidentalis
μ: 10
σ: 2.96
H: 3.2
R²: 0.14
Triadenum.fraseri
μ: 10
σ: 2
H: 2.1
R²: 0.13
Typha.latifolia
μ: 3.38
σ: 2
H: 1.6
R²: 0.098
Urtica.dioica
μ: 3.93
σ: 2
H: 2.4
R²: 0.14
Utricularia.intermedia
μ: 8.27
σ: 2.17
H: 3
R²: 0.24
Probability
Environmental Condition (C_{env})

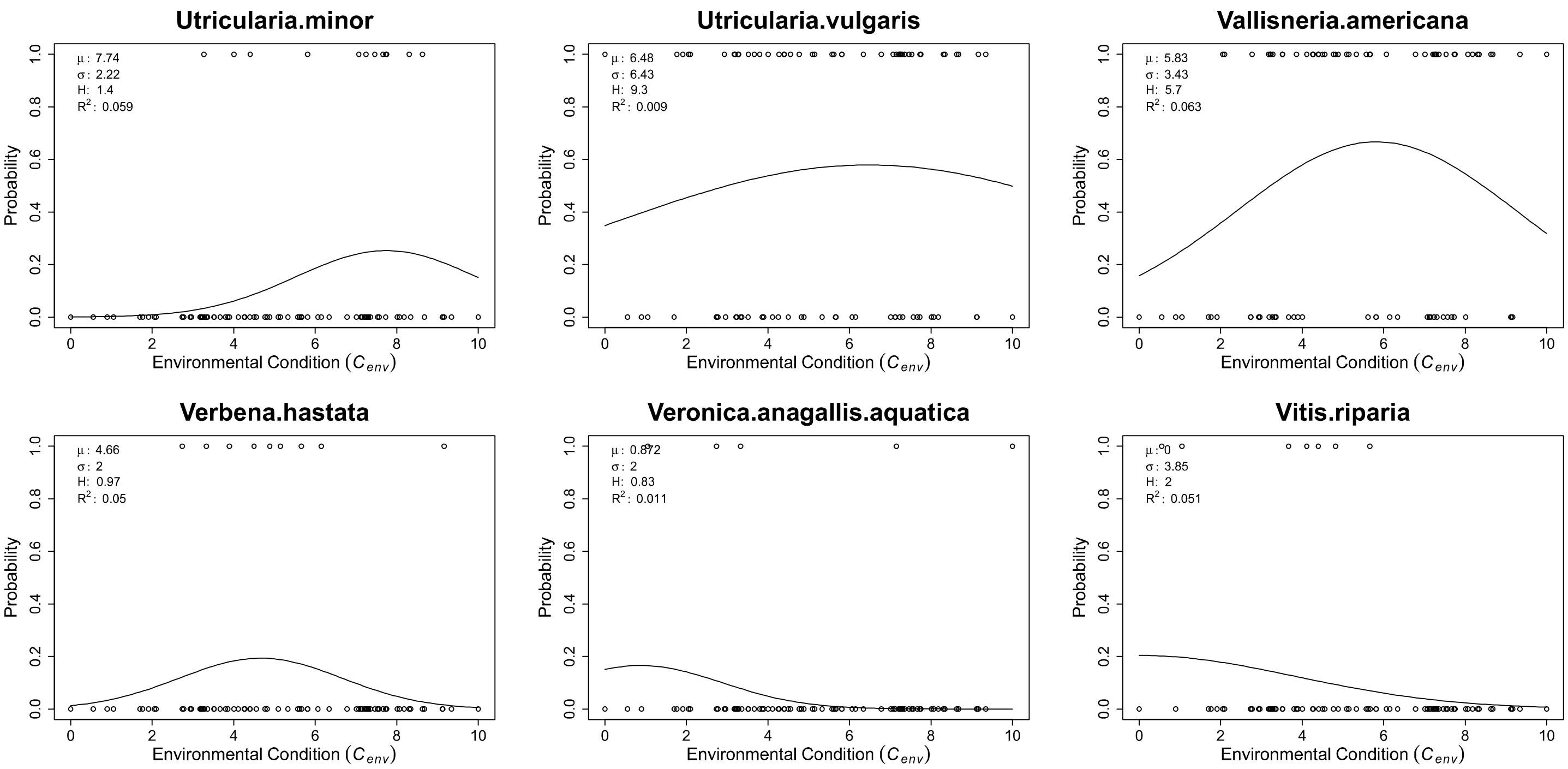
Utricularia.minor
μ: 7.74
σ: 2.22
H: 1.4
R²: 0.059
Utricularia.vulgaris
μ: 6.48
σ: 6.43
H: 9.3
R²: 0.009
Vallisneria.americana
μ: 5.83
σ: 3.43
H: 5.7
R²: 0.063
Verbena.hastata
μ: 4.66
σ: 2
H: 0.97
R²: 0.05
Veronica.anagallis.aquatica
μ: 0.872
σ: 2
H: 0.83
R²: 0.011
Vitis.riparia
μ: 0
σ: 3.85
H: 2
R²: 0.051
Probability
Environmental Condition (C_env)
0.0
0.2
0.4
0.6
0.8
1.0
0
2
4
6
8
10
