# Supplementary material for: Beyond Floristic Quality: A Data-Driven Approach to Assessing Coastal Wetland Condition Using Plants
Source: Wetlands (Wilmington). 2026 Oct 6;46(7):129. doi: 10.1007/s13157-026-02094-z (PMC13642451; doi:10.1007/s13157-026-02094-z)
Supplement: Supplementary file 4 [file 13157_2026_2094_MOESM4_ESM.pdf]

Wetlands

Online Resource 1: Lake Ontario biotic response curves

Beyond floristic quality: A data-driven approach to assessing coastal wetland condition using plants

Giuseppe E. Fiorino[1]*, Simon T. Denomme-Brown[1], Hayley N. Rogers[1], Ian M. Smith[1], Kathryn L. Amatangelo[2], Matthew J. Cooper[3], Nicholas P. Danz[4], Robert W. Howe[5], Rachel E. Schultz[2], Bridget A. Wheelock[6], Douglas A. Wilcox[2], Greg P. Grabas[1]

[1]Environment and Climate Change Canada, 4905 Dufferin Street, Toronto, Ontario M3H 5T4, Canada

[2]Department of Environmental Science and Ecology, SUNY-Brockport, 350 New Campus Drive, Brockport, NY 14420, USA

[3]Department of Biology, Grand Valley State University, Allendale, MI 49401, USA

[4]Department of Natural Sciences, University of Wisconsin-Superior, 1605 Catlin Avenue, Superior, WI 54880, USA

[5]Cofrin Center for Biodiversity, University of Wisconsin-Green Bay, Green Bay, WI 54311-7001, USA

[6]Institute for Great Lakes Research and Department of Biology, Central Michigan University, Mount Pleasant, MI, USA

*Corresponding Author (email: giuseppe.fiorino@ec.gc.ca, ORCID: 0000-0002-1569-0767)

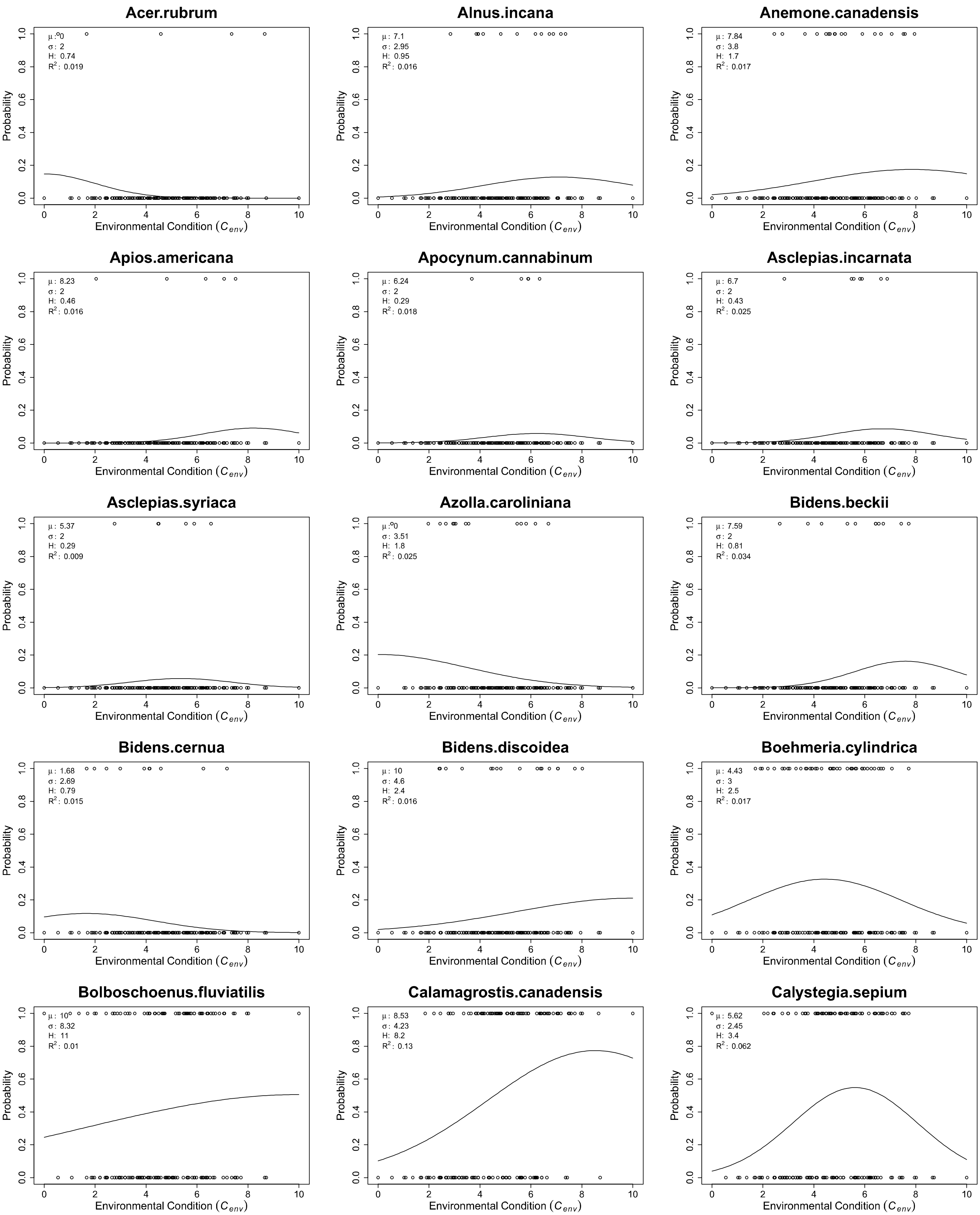
Acer.rubrum
μ: 0
σ: 2
H: 0.74
R²: 0.019
Alnus.incana
μ: 7.1
σ: 2.95
H: 0.95
R²: 0.016
Anemone.canadensis
μ: 7.84
σ: 3.8
H: 1.7
R²: 0.017
Apios.americana
μ: 8.23
σ: 2
H: 0.46
R²: 0.016
Apocynum.cannabinum
μ: 6.24
σ: 2
H: 0.29
R²: 0.018
Asclepias.incarnata
μ: 6.7
σ: 2
H: 0.43
R²: 0.025
Asclepias.syriaca
μ: 5.37
σ: 2
H: 0.29
R²: 0.009
Azolla.caroliniana
μ: 0
σ: 3.51
H: 1.8
R²: 0.025
Bidens.beckii
μ: 7.59
σ: 2
H: 0.81
R²: 0.034
Bidens.cernua
μ: 1.68
σ: 2.69
H: 0.79
R²: 0.015
Bidens.discoidea
μ: 10
σ: 4.6
H: 2.4
R²: 0.016
Boehmeria.cylindrica
μ: 4.43
σ: 3
H: 2.5
R²: 0.017
Bolboschoenus.fluviatilis
μ: 10
σ: 8.32
H: 11
R²: 0.01
Calamagrostis.canadensis
μ: 8.53
σ: 4.23
H: 8.2
R²: 0.13
Calystegia.sepium
μ: 5.62
σ: 2.45
H: 3.4
R²: 0.062
Probability
Environmental Condition (C_{env})

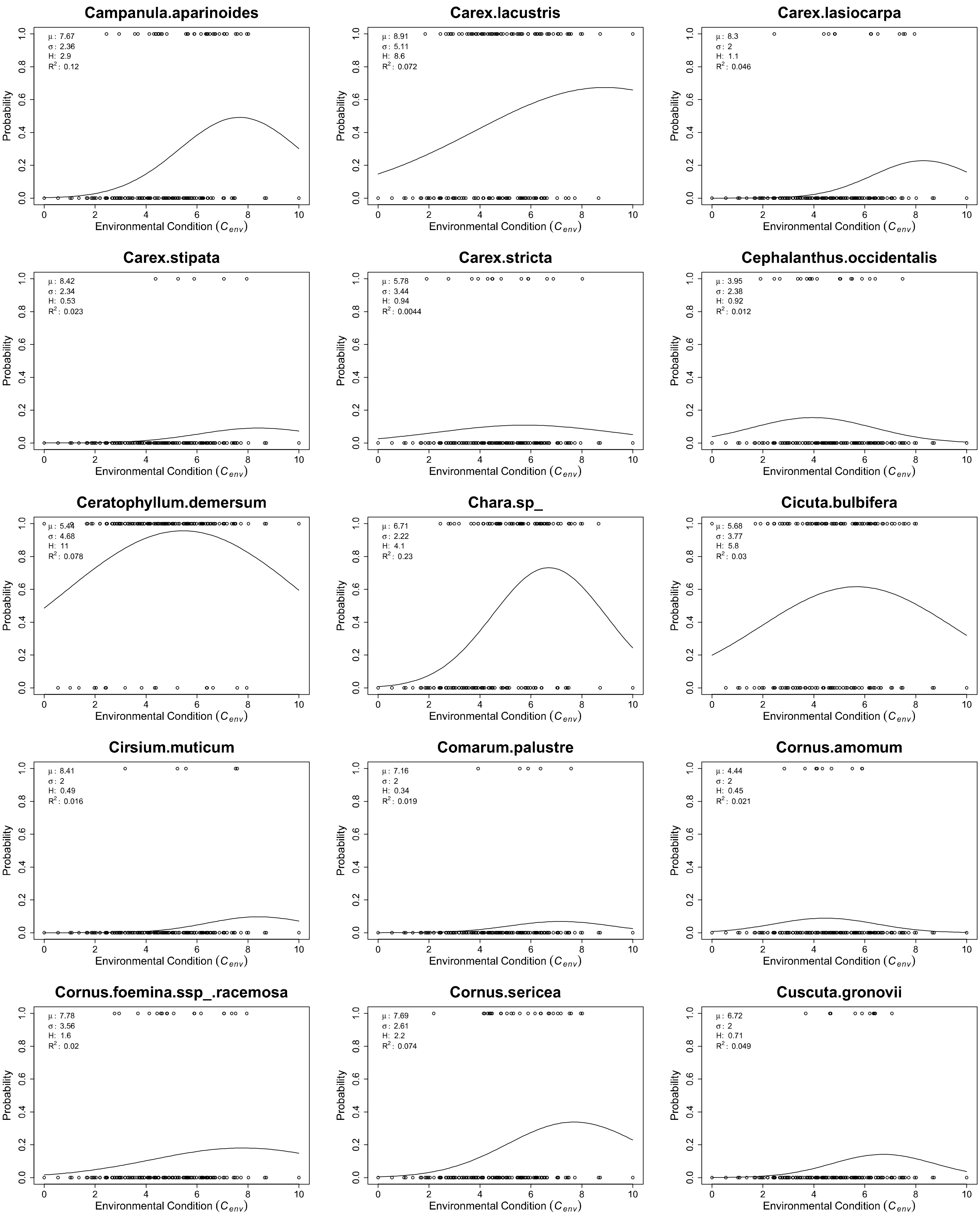
Campanula.aparinoides
μ: 7.67
σ: 2.36
H: 2.9
R²: 0.12
Carex.lacustris
μ: 8.91
σ: 5.11
H: 8.6
R²: 0.072
Carex.lasiocarpa
μ: 8.3
σ: 2
H: 1.1
R²: 0.046
Carex.stipata
μ: 8.42
σ: 2.34
H: 0.53
R²: 0.023
Carex.stricta
μ: 5.78
σ: 3.44
H: 0.94
R²: 0.0044
Cephalanthus.occidentalis
μ: 3.95
σ: 2.38
H: 0.92
R²: 0.012
Ceratophyllum.demersum
μ: 5.44
σ: 4.68
H: 11
R²: 0.078
Chara.sp_
μ: 6.71
σ: 2.22
H: 4.1
R²: 0.23
Cicuta.bulbifera
μ: 5.68
σ: 3.77
H: 5.8
R²: 0.03
Cirsium.muticum
μ: 8.41
σ: 2
H: 0.49
R²: 0.016
Comarum.palustre
μ: 7.16
σ: 2
H: 0.34
R²: 0.019
Cornus.amomum
μ: 4.44
σ: 2
H: 0.45
R²: 0.021
Cornus.foemina.ssp_.racemosa
μ: 7.78
σ: 3.56
H: 1.6
R²: 0.02
Cornus.sericea
μ: 7.69
σ: 2.61
H: 2.2
R²: 0.074
Cuscuta.gronovii
μ: 6.72
σ: 2
H: 0.71
R²: 0.049
Probability
Environmental Condition (C_{env})

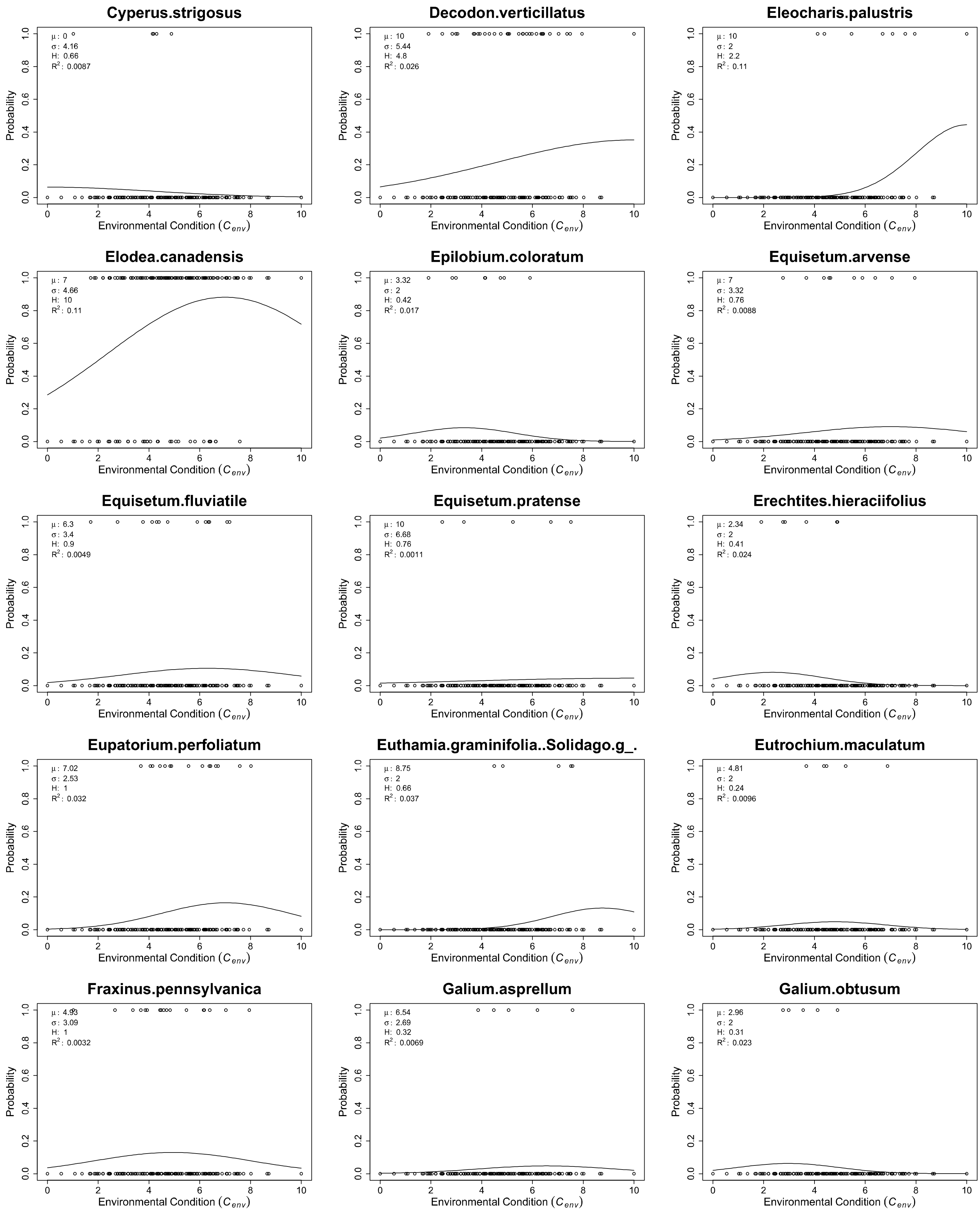
Cyperus.strigosus
μ: 0
σ: 4.16
H: 0.66
R^2: 0.0087
Decodon.verticillatus
μ: 10
σ: 5.44
H: 4.8
R^2: 0.026
Eleocharis.palustris
μ: 10
σ: 2
H: 2.2
R^2: 0.11
Elodea.canadensis
μ: 7
σ: 4.66
H: 10
R^2: 0.11
Epilobium.coloratum
μ: 3.32
σ: 2
H: 0.42
R^2: 0.017
Equisetum.arvense
μ: 7
σ: 3.32
H: 0.76
R^2: 0.0088
Equisetum.fluviatile
μ: 6.3
σ: 3.4
H: 0.9
R^2: 0.0049
Equisetum.pratense
μ: 10
σ: 6.68
H: 0.76
R^2: 0.0011
Erechtites.hieraciifolius
μ: 2.34
σ: 2
H: 0.41
R^2: 0.024
Eupatorium.perfoliatum
μ: 7.02
σ: 2.53
H: 1
R^2: 0.032
Euthamia.graminifolia..Solidago.g_.
μ: 8.75
σ: 2
H: 0.66
R^2: 0.037
Eutrochium.maculatum
μ: 4.81
σ: 2
H: 0.24
R^2: 0.0096
Fraxinus.pennsylvanica
μ: 4.93
σ: 3.09
H: 1
R^2: 0.0032
Galium.asprellum
μ: 6.54
σ: 2.69
H: 0.32
R^2: 0.0069
Galium.obtusum
μ: 2.96
σ: 2
H: 0.31
R^2: 0.023
Probability
Environmental Condition (C_{env})

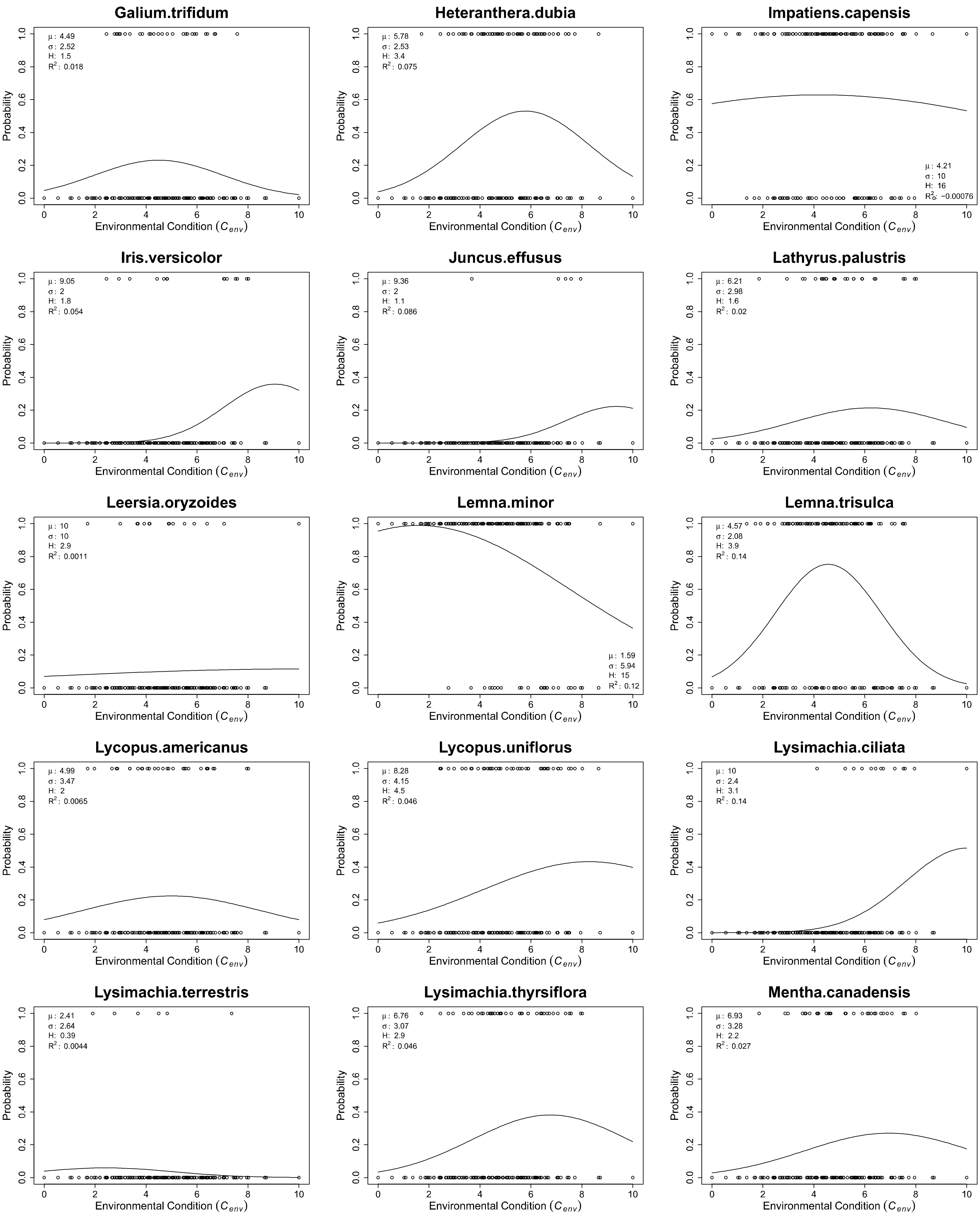
Galium.trifidum
μ: 4.49
σ: 2.52
H: 1.5
R2: 0.018
Heteranthera.dubia
μ: 5.78
σ: 2.53
H: 3.4
R2: 0.075
Impatiens.capensis
μ: 4.21
σ: 10
H: 16
R2: −0.00076
Iris.versicolor
μ: 9.05
σ: 2
H: 1.8
R2: 0.054
Juncus.effusus
μ: 9.36
σ: 2
H: 1.1
R2: 0.086
Lathyrus.palustris
μ: 6.21
σ: 2.98
H: 1.6
R2: 0.02
Leersia.oryzoides
μ: 10
σ: 10
H: 2.9
R2: 0.0011
Lemna.minor
μ: 1.59
σ: 5.94
H: 15
R2: 0.12
Lemna.trisulca
μ: 4.57
σ: 2.08
H: 3.9
R2: 0.14
Lycopus.americanus
μ: 4.99
σ: 3.47
H: 2
R2: 0.0065
Lycopus.uniflorus
μ: 8.28
σ: 4.15
H: 4.5
R2: 0.046
Lysimachia.ciliata
μ: 10
σ: 2.4
H: 3.1
R2: 0.14
Lysimachia.terrestris
μ: 2.41
σ: 2.64
H: 0.39
R2: 0.0044
Lysimachia.thyrsiflora
μ: 6.76
σ: 3.07
H: 2.9
R2: 0.046
Mentha.canadensis
μ: 6.93
σ: 3.28
H: 2.2
R2: 0.027
Probability
Environmental Condition (Cenv)

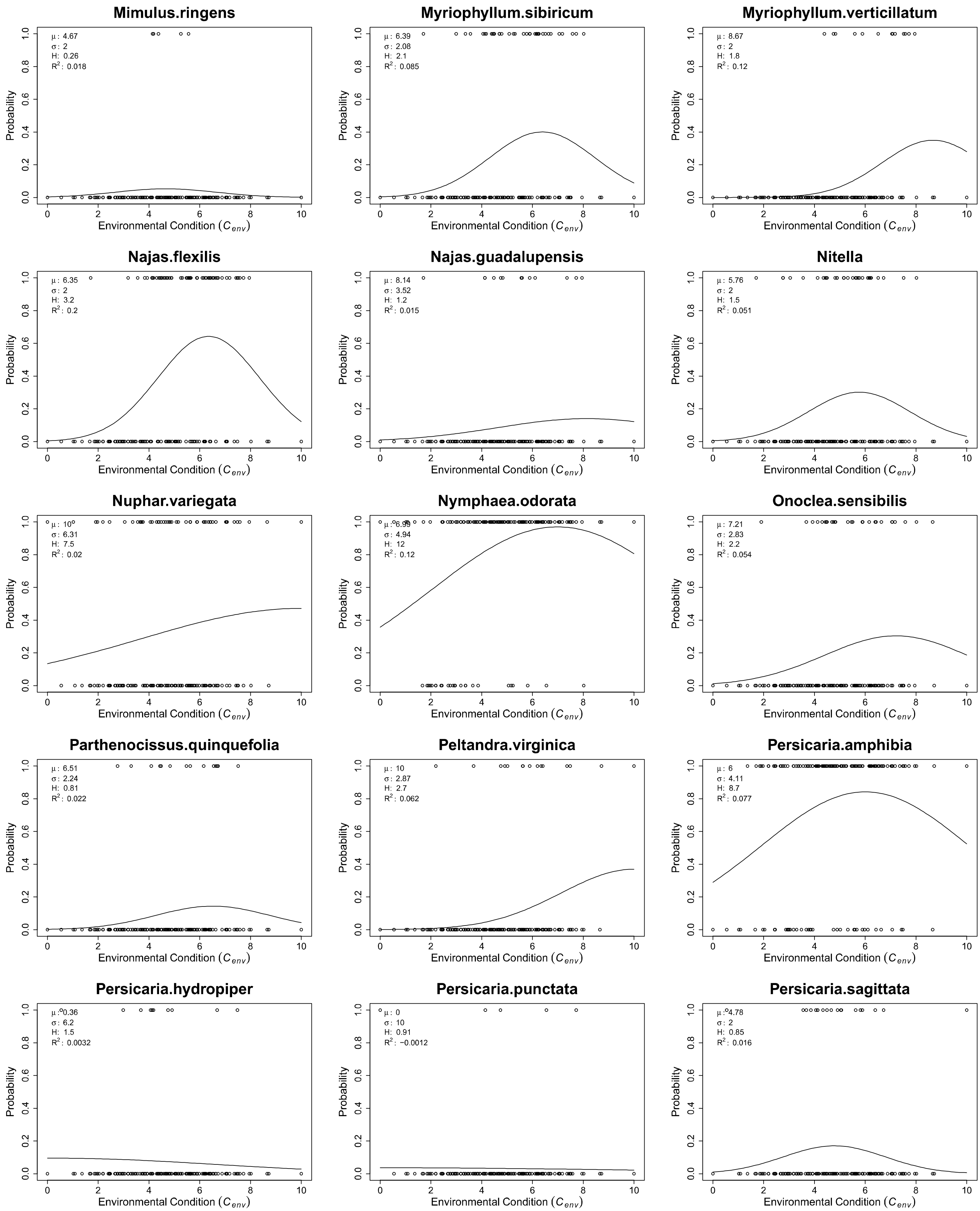
Mimulus.ringens
μ: 4.67
σ: 2
H: 0.26
R²: 0.018
Myriophyllum.sibiricum
μ: 6.39
σ: 2.08
H: 2.1
R²: 0.085
Myriophyllum.verticillatum
μ: 8.67
σ: 2
H: 1.8
R²: 0.12
Najas.flexilis
μ: 6.35
σ: 2
H: 3.2
R²: 0.2
Najas.guadalupensis
μ: 8.14
σ: 3.52
H: 1.2
R²: 0.015
Nitella
μ: 5.76
σ: 2
H: 1.5
R²: 0.051
Nuphar.variegata
μ: 10
σ: 6.31
H: 7.5
R²: 0.02
Nymphaea.odorata
σ: 4.94
H: 12
R²: 0.12
Onoclea.sensibilis
μ: 7.21
σ: 2.83
H: 2.2
R²: 0.054
Parthenocissus.quinquefolia
μ: 6.51
σ: 2.24
H: 0.81
R²: 0.022
Peltandra.virginica
μ: 10
σ: 2.87
H: 2.7
R²: 0.062
Persicaria.amphibia
μ: 6
σ: 4.11
H: 8.7
R²: 0.077
Persicaria.hydropiper
σ: 6.2
H: 1.5
R²: 0.0032
Persicaria.punctata
μ: 0
σ: 10
H: 0.91
R²: −0.0012
Persicaria.sagittata
σ: 2
H: 0.85
R²: 0.016
Probability
Environmental Condition (C_{env})

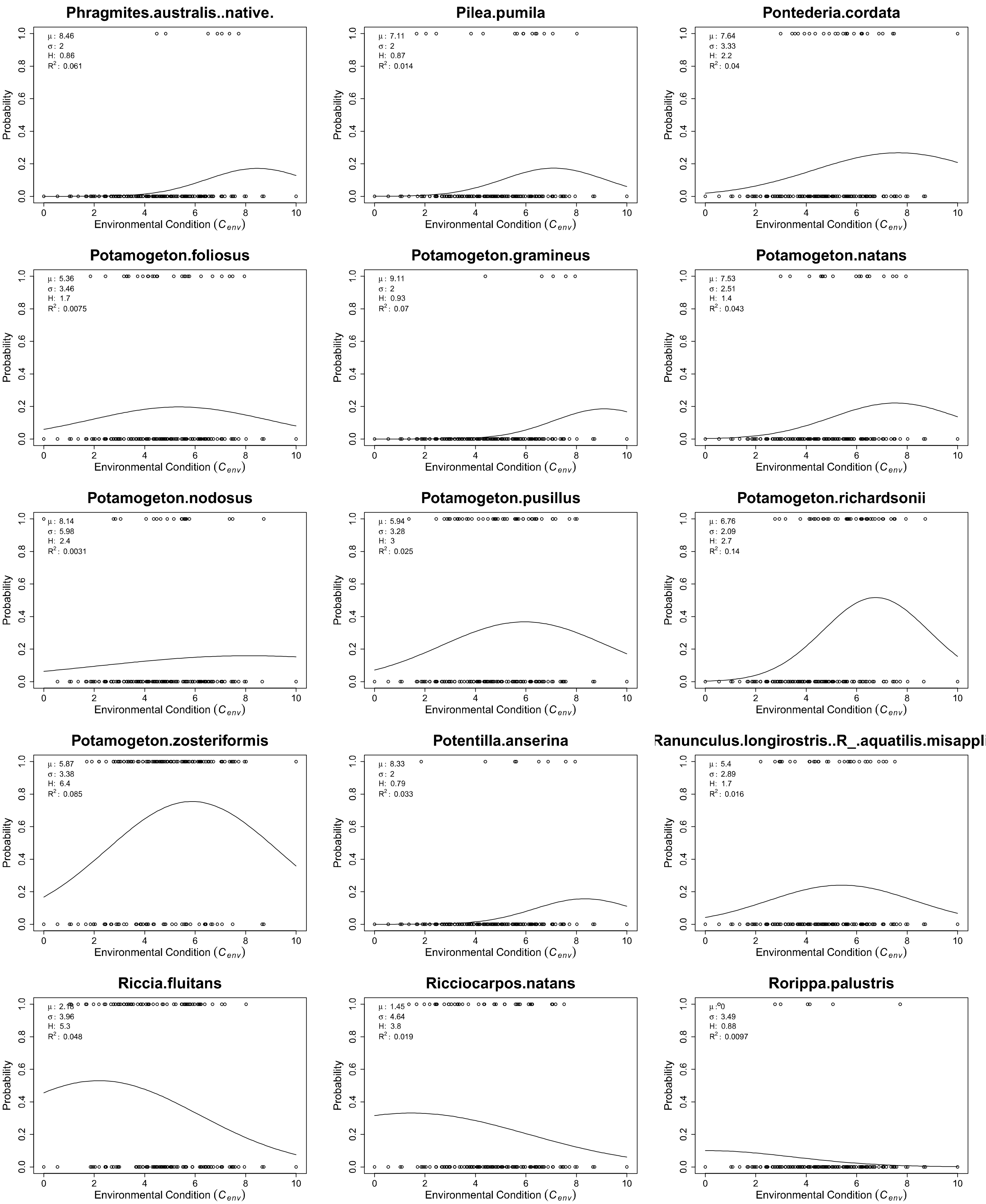
Phragmites.australis..native.
μ: 8.46
σ: 2
H: 0.86
R²: 0.061
Pilea.pumila
μ: 7.11
σ: 2
H: 0.87
R²: 0.014
Pontederia.cordata
μ: 7.64
σ: 3.33
H: 2.2
R²: 0.04
Potamogeton.foliosus
μ: 5.36
σ: 3.46
H: 1.7
R²: 0.0075
Potamogeton.gramineus
μ: 9.11
σ: 2
H: 0.93
R²: 0.07
Potamogeton.natans
μ: 7.53
σ: 2.51
H: 1.4
R²: 0.043
Potamogeton.nodosus
μ: 8.14
σ: 5.98
H: 2.4
R²: 0.0031
Potamogeton.pusillus
μ: 5.94
σ: 3.28
H: 3
R²: 0.025
Potamogeton.richardsonii
μ: 6.76
σ: 2.09
H: 2.7
R²: 0.14
Potamogeton.zosteriformis
μ: 5.87
σ: 3.38
H: 6.4
R²: 0.085
Potentilla.anserina
μ: 8.33
σ: 2
H: 0.79
R²: 0.033
Ranunculus.longirostris..R_.aquatilis.misappli
μ: 5.4
σ: 2.89
H: 1.7
R²: 0.016
Riccia.fluitans
μ: 2.18
σ: 3.96
H: 5.3
R²: 0.048
Ricciocarpos.natans
μ: 1.45
σ: 4.64
H: 3.8
R²: 0.019
Rorippa.palustris
μ: 0
σ: 3.49
H: 0.88
R²: 0.0097
Probability
Environmental Condition (C_{env})

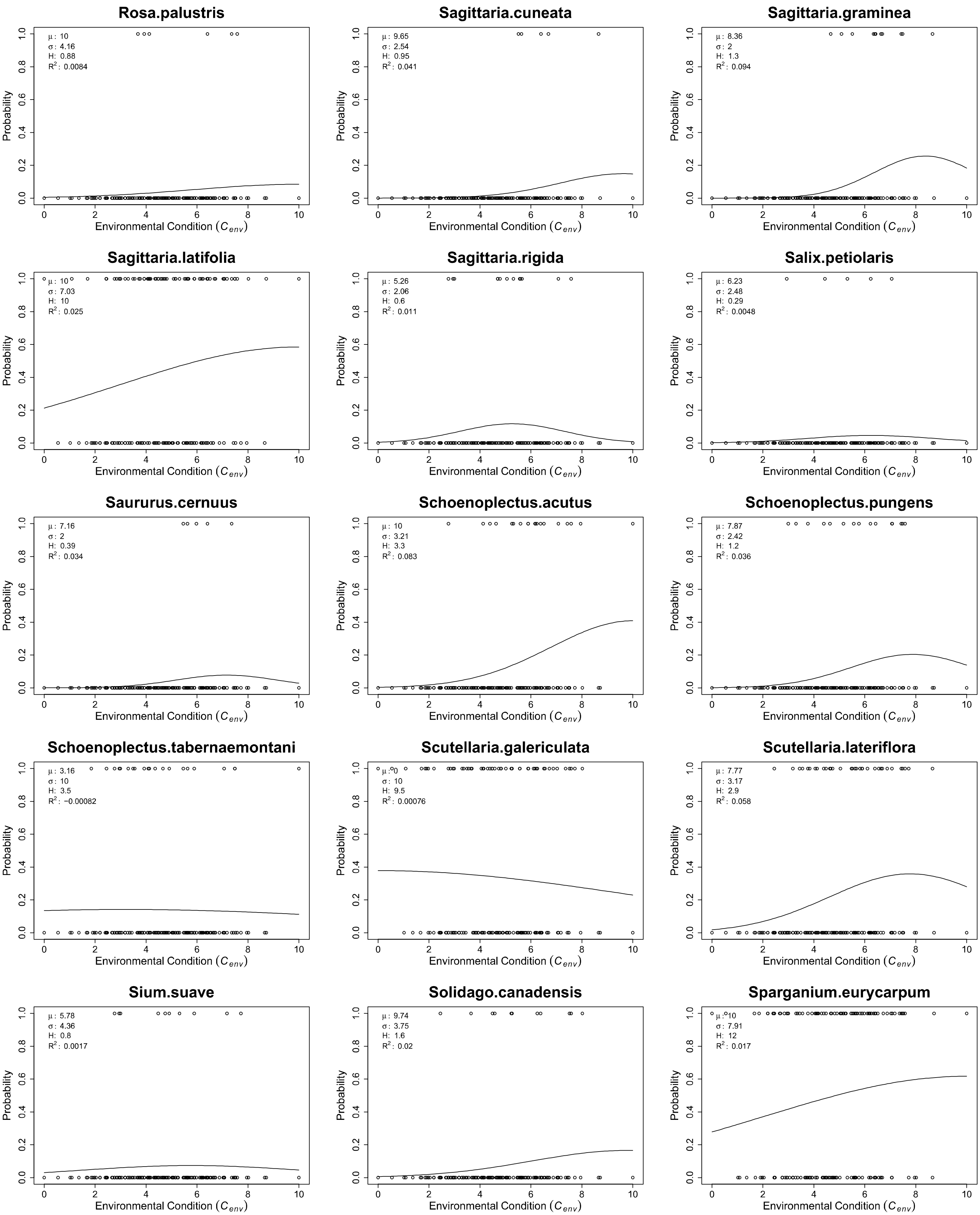
Rosa.palustris
μ: 10
σ: 4.16
H: 0.88
R²: 0.0084
Sagittaria.cuneata
μ: 9.65
σ: 2.54
H: 0.95
R²: 0.041
Sagittaria.graminea
μ: 8.36
σ: 2
H: 1.3
R²: 0.094
Sagittaria.latifolia
μ: 10
σ: 7.03
H: 10
R²: 0.025
Sagittaria.rigida
μ: 5.26
σ: 2.06
H: 0.6
R²: 0.011
Salix.petiolaris
μ: 6.23
σ: 2.48
H: 0.29
R²: 0.0048
Saururus.cernuus
μ: 7.16
σ: 2
H: 0.39
R²: 0.034
Schoenoplectus.acutus
μ: 10
σ: 3.21
H: 3.3
R²: 0.083
Schoenoplectus.pungens
μ: 7.87
σ: 2.42
H: 1.2
R²: 0.036
Schoenoplectus.tabernaemontani
μ: 3.16
σ: 10
H: 3.5
R²: −0.00082
Scutellaria.galericulata
μ: 0
σ: 10
H: 9.5
R²: 0.00076
Scutellaria.lateriflora
μ: 7.77
σ: 3.17
H: 2.9
R²: 0.058
Sium.suave
μ: 5.78
σ: 4.36
H: 0.8
R²: 0.0017
Solidago.canadensis
μ: 9.74
σ: 3.75
H: 1.6
R²: 0.02
Sparganium.eurycarpum
μ: 10
σ: 7.91
H: 12
R²: 0.017
Probability
Environmental Condition (Cenv)

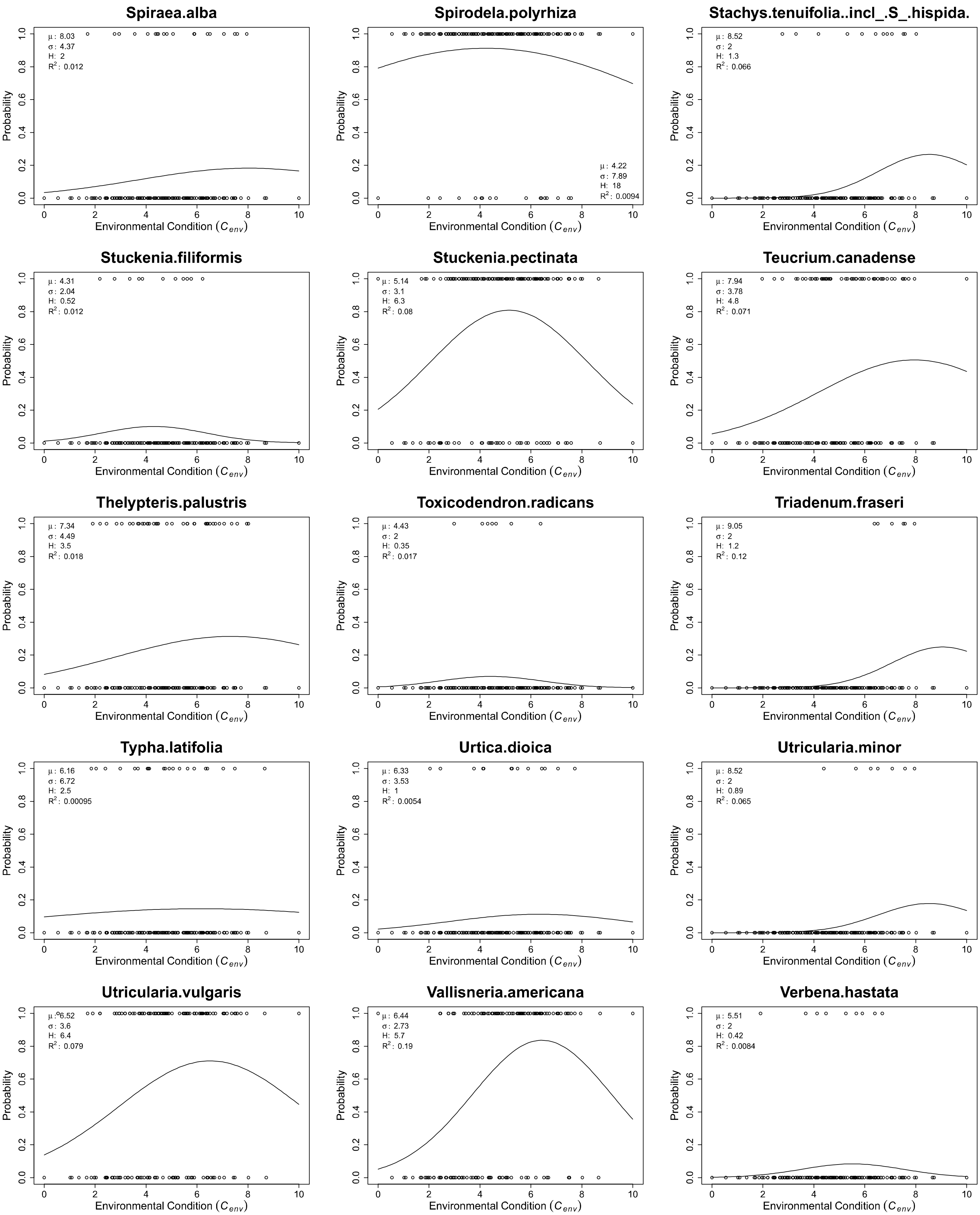
Spiraea.alba
μ: 8.03
σ: 4.37
H: 2
R²: 0.012
Spirodela.polyrhiza
μ: 4.22
σ: 7.89
H: 18
R²: 0.0094
Stachys.tenuifolia..incl_.S_.hispida.
μ: 8.52
σ: 2
H: 1.3
R²: 0.066
Stuckenia.filiformis
μ: 4.31
σ: 2.04
H: 0.52
R²: 0.012
Stuckenia.pectinata
μ: 5.14
σ: 3.1
H: 6.3
R²: 0.08
Teucrium.canadense
μ: 7.94
σ: 3.78
H: 4.8
R²: 0.071
Thelypteris.palustris
μ: 7.34
σ: 4.49
H: 3.5
R²: 0.018
Toxicodendron.radicans
μ: 4.43
σ: 2
H: 0.35
R²: 0.017
Triadenum.fraseri
μ: 9.05
σ: 2
H: 1.2
R²: 0.12
Typha.latifolia
μ: 6.16
σ: 6.72
H: 2.5
R²: 0.00095
Urtica.dioica
μ: 6.33
σ: 3.53
H: 1
R²: 0.0054
Utricularia.minor
μ: 8.52
σ: 2
H: 0.89
R²: 0.065
Utricularia.vulgaris
μ: 6.52
σ: 3.6
H: 6.4
R²: 0.079
Vallisneria.americana
μ: 6.44
σ: 2.73
H: 5.7
R²: 0.19
Verbena.hastata
μ: 5.51
σ: 2
H: 0.42
R²: 0.0084
Probability
Environmental Condition (C_{env})

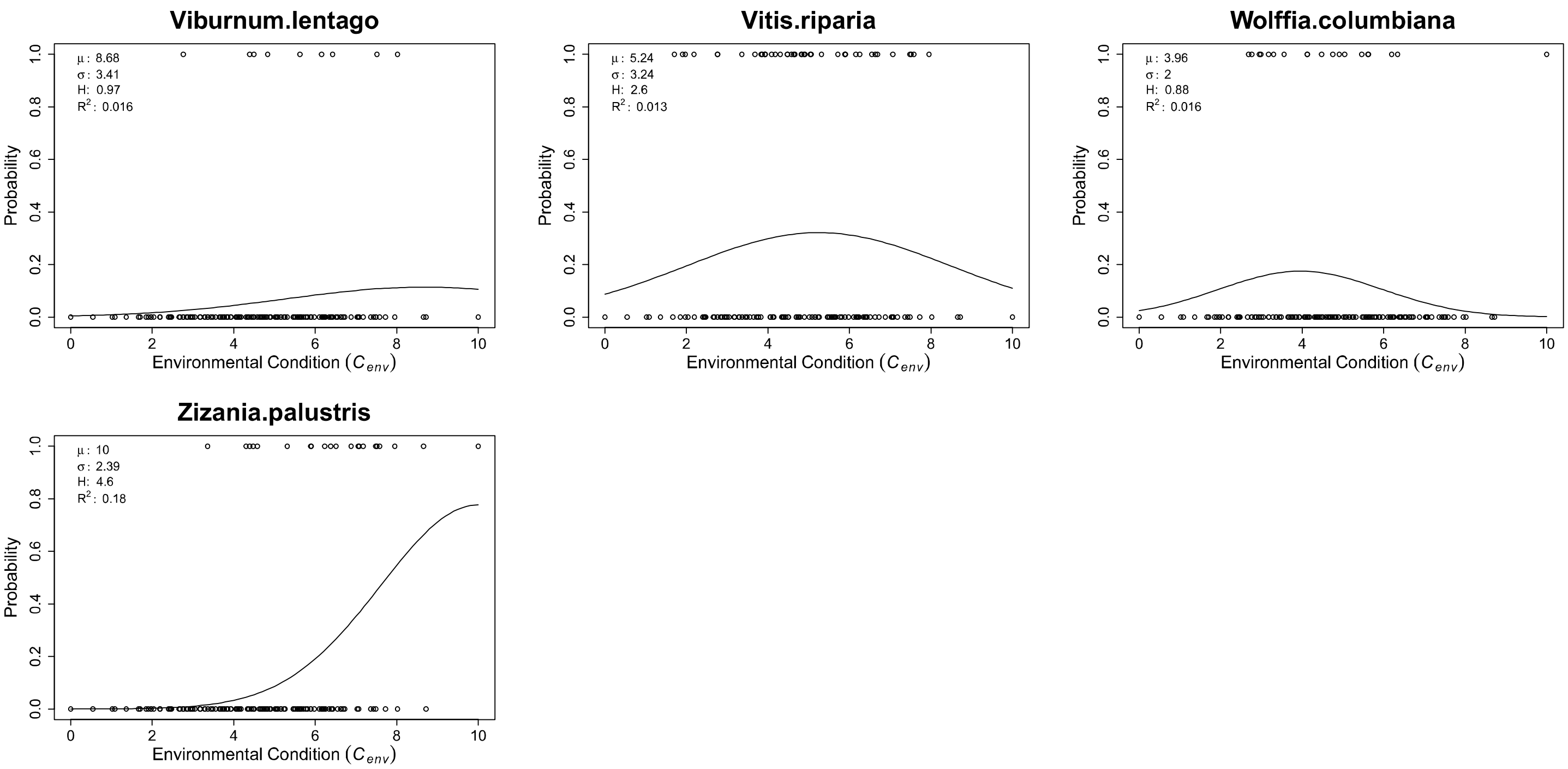
Viburnum.lentago
μ: 8.68
σ: 3.41
H: 0.97
R²: 0.016
Vitis.riparia
μ: 5.24
σ: 3.24
H: 2.6
R²: 0.013
Wolffia.columbiana
μ: 3.96
σ: 2
H: 0.88
R²: 0.016
Zizania.palustris
μ: 10
σ: 2.39
H: 4.6
R²: 0.18
Probability
Environmental Condition (C_env)
0.0
0.2
0.4
0.6
0.8
1.0
0
2
4
6
8
10
