# Supplementary material for: Beyond Floristic Quality: A Data-Driven Approach to Assessing Coastal Wetland Condition Using Plants
Source: Wetlands (Wilmington). 2026 Oct 6;46(7):129. doi: 10.1007/s13157-026-02094-z (PMC13642451; doi:10.1007/s13157-026-02094-z)
Supplement: Supplementary file 5 [file 13157_2026_2094_MOESM5_ESM.pdf]

Wetlands

Online Resource 1: Lake Superior biotic response curves

Beyond floristic quality: A data-driven approach to assessing coastal wetland condition using plants

Giuseppe E. Fiorino[1]*, Simon T. Denomme-Brown[1], Hayley N. Rogers[1], Ian M. Smith[1], Kathryn L. Amatangelo[2], Matthew J. Cooper[3], Nicholas P. Danz[4], Robert W. Howe[5], Rachel E. Schultz[2], Bridget A. Wheelock[6], Douglas A. Wilcox[2], Greg P. Grabas[1]

[1]Environment and Climate Change Canada, 4905 Dufferin Street, Toronto, Ontario M3H 5T4, Canada

[2]Department of Environmental Science and Ecology, SUNY-Brockport, 350 New Campus Drive, Brockport, NY 14420, USA

[3]Department of Biology, Grand Valley State University, Allendale, MI 49401, USA

[4]Department of Natural Sciences, University of Wisconsin-Superior, 1605 Catlin Avenue, Superior, WI 54880, USA

[5]Cofrin Center for Biodiversity, University of Wisconsin-Green Bay, Green Bay, WI 54311-7001, USA

[6]Institute for Great Lakes Research and Department of Biology, Central Michigan University, Mount Pleasant, MI, USA

*Corresponding Author (email: giuseppe.fiorino@ec.gc.ca, ORCID: 0000-0002-1569-0767)

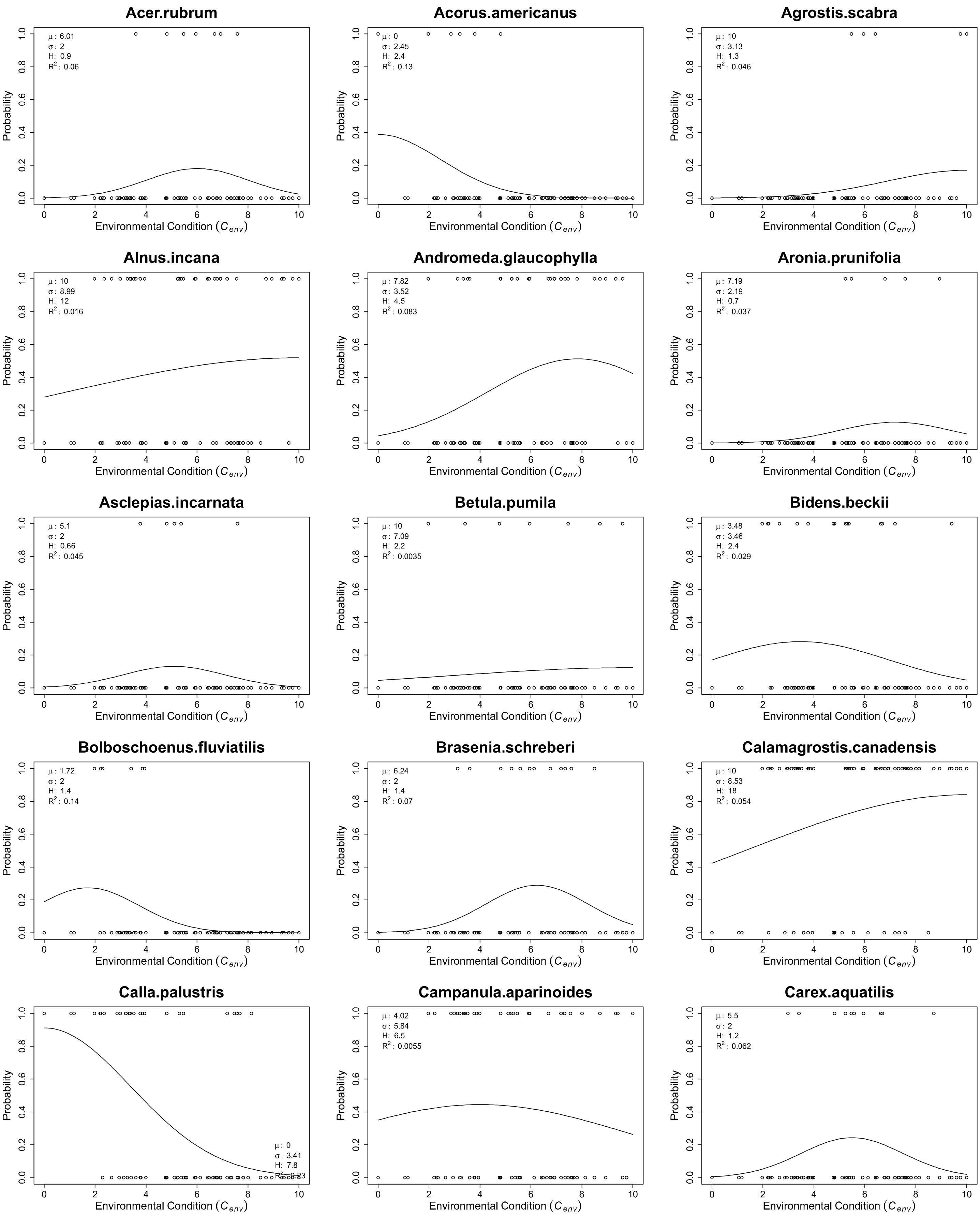
Acer.rubrum
μ: 6.01
σ: 2
H: 0.9
R²: 0.06
Acorus.americanus
μ: 0
σ: 2.45
H: 2.4
R²: 0.13
Agrostis.scabra
μ: 10
σ: 3.13
H: 1.3
R²: 0.046
Alnus.incana
μ: 10
σ: 8.99
H: 12
R²: 0.016
Andromeda.glaucophylla
μ: 7.82
σ: 3.52
H: 4.5
R²: 0.083
Aronia.prunifolia
μ: 7.19
σ: 2.19
H: 0.7
R²: 0.037
Asclepias.incarnata
μ: 5.1
σ: 2
H: 0.66
R²: 0.045
Betula.pumila
μ: 10
σ: 7.09
H: 2.2
R²: 0.0035
Bidens.beckii
μ: 3.48
σ: 3.46
H: 2.4
R²: 0.029
Bolboschoenus.fluviatilis
μ: 1.72
σ: 2
H: 1.4
R²: 0.14
Brasenia.schreberi
μ: 6.24
σ: 2
H: 1.4
R²: 0.07
Calamagrostis.canadensis
μ: 10
σ: 8.53
H: 18
R²: 0.054
Calla.palustris
μ: 0
σ: 3.41
H: 7.8
R²: 0.23
Campanula.aparinoides
μ: 4.02
σ: 5.84
H: 6.5
R²: 0.0055
Carex.aquatilis
μ: 5.5
σ: 2
H: 1.2
R²: 0.062
Probability
Environmental Condition (C_{env})

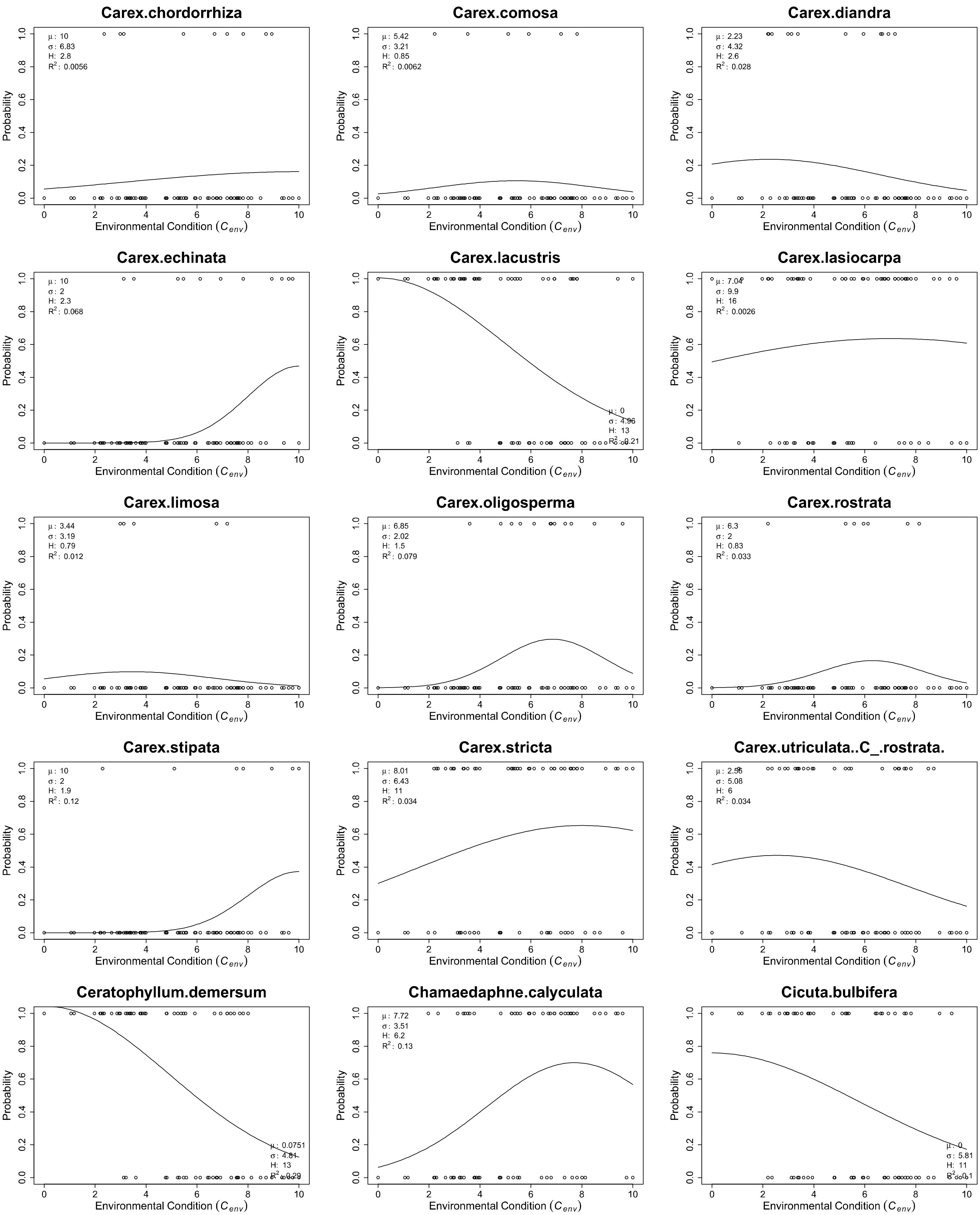
Carex.chordorrhiza
μ: 10
σ: 6.83
H: 2.8
R²: 0.0056
Carex.comosa
μ: 5.42
σ: 3.21
H: 0.85
R²: 0.0062
Carex.diandra
μ: 2.23
σ: 4.32
H: 2.6
R²: 0.028
Carex.echinata
μ: 10
σ: 2
H: 2.3
R²: 0.068
Carex.lacustris
μ: 0
σ: 4.96
H: 13
R²: 0.21
Carex.lasiocarpa
μ: 7.04
σ: 9.9
H: 16
R²: 0.0026
Carex.limosa
μ: 3.44
σ: 3.19
H: 0.79
R²: 0.012
Carex.oligosperma
μ: 6.85
σ: 2.02
H: 1.5
R²: 0.079
Carex.rostrata
μ: 6.3
σ: 2
H: 0.83
R²: 0.033
Carex.stipata
μ: 10
σ: 2
H: 1.9
R²: 0.12
Carex.stricta
μ: 8.01
σ: 6.43
H: 11
R²: 0.034
Carex.utriculata..C_.rostrata.
μ: 2.56
σ: 5.08
H: 6
R²: 0.034
Ceratophyllum.demersum
μ: 0.0751
σ: 4.81
H: 13
Chamaedaphne.calyculata
μ: 7.72
σ: 3.51
H: 6.2
R²: 0.13
Cicuta.bulbifera
μ: 0
σ: 5.81
H: 11
Probability
Environmental Condition (C_{env})

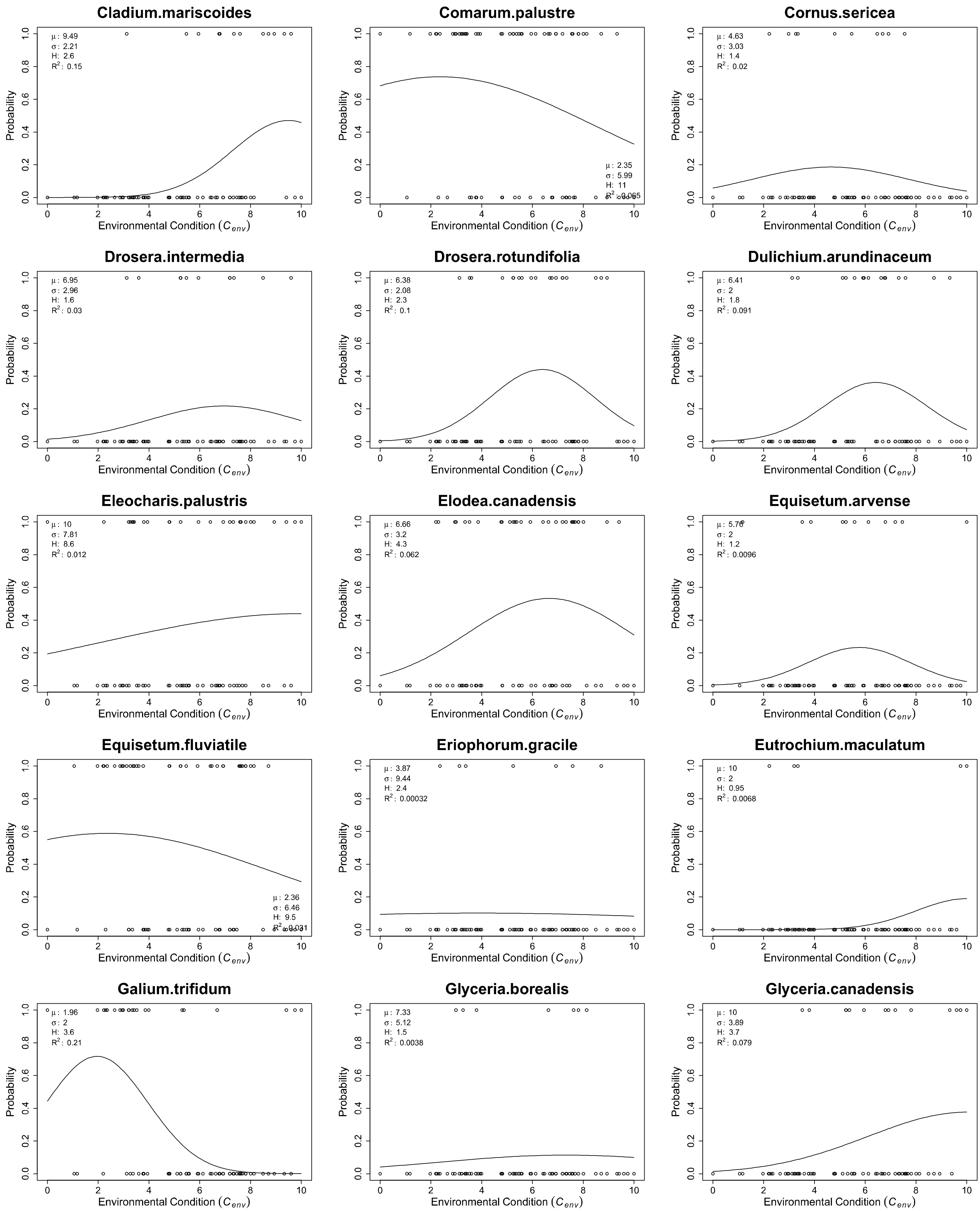

Cladium.mariscoides
μ: 9.49
σ: 2.21
H: 2.6
R²: 0.15
Comarum.palustre
μ: 2.35
σ: 5.99
H: 11
Cornus.sericea
μ: 4.63
σ: 3.03
H: 1.4
R²: 0.02
Drosera.intermedia
μ: 6.95
σ: 2.96
H: 1.6
R²: 0.03
Drosera.rotundifolia
μ: 6.38
σ: 2.08
H: 2.3
R²: 0.1
Dulichium.arundinaceum
μ: 6.41
σ: 2
H: 1.8
R²: 0.091
Eleocharis.palustris
μ: 10
σ: 7.81
H: 8.6
R²: 0.012
Elodea.canadensis
μ: 6.66
σ: 3.2
H: 4.3
R²: 0.062
Equisetum.arvense
σ: 2
H: 1.2
R²: 0.0096
Equisetum.fluviatile
μ: 2.36
σ: 6.46
H: 9.5
Eriophorum.gracile
μ: 3.87
σ: 9.44
H: 2.4
R²: 0.00032
Eutrochium.maculatum
μ: 10
σ: 2
H: 0.95
R²: 0.0068
Galium.trifidum
μ: 1.96
σ: 2
H: 3.6
R²: 0.21
Glyceria.borealis
μ: 7.33
σ: 5.12
H: 1.5
R²: 0.0038
Glyceria.canadensis
μ: 10
σ: 3.89
H: 3.7
R²: 0.079
Probability
Environmental Condition (C_{env})

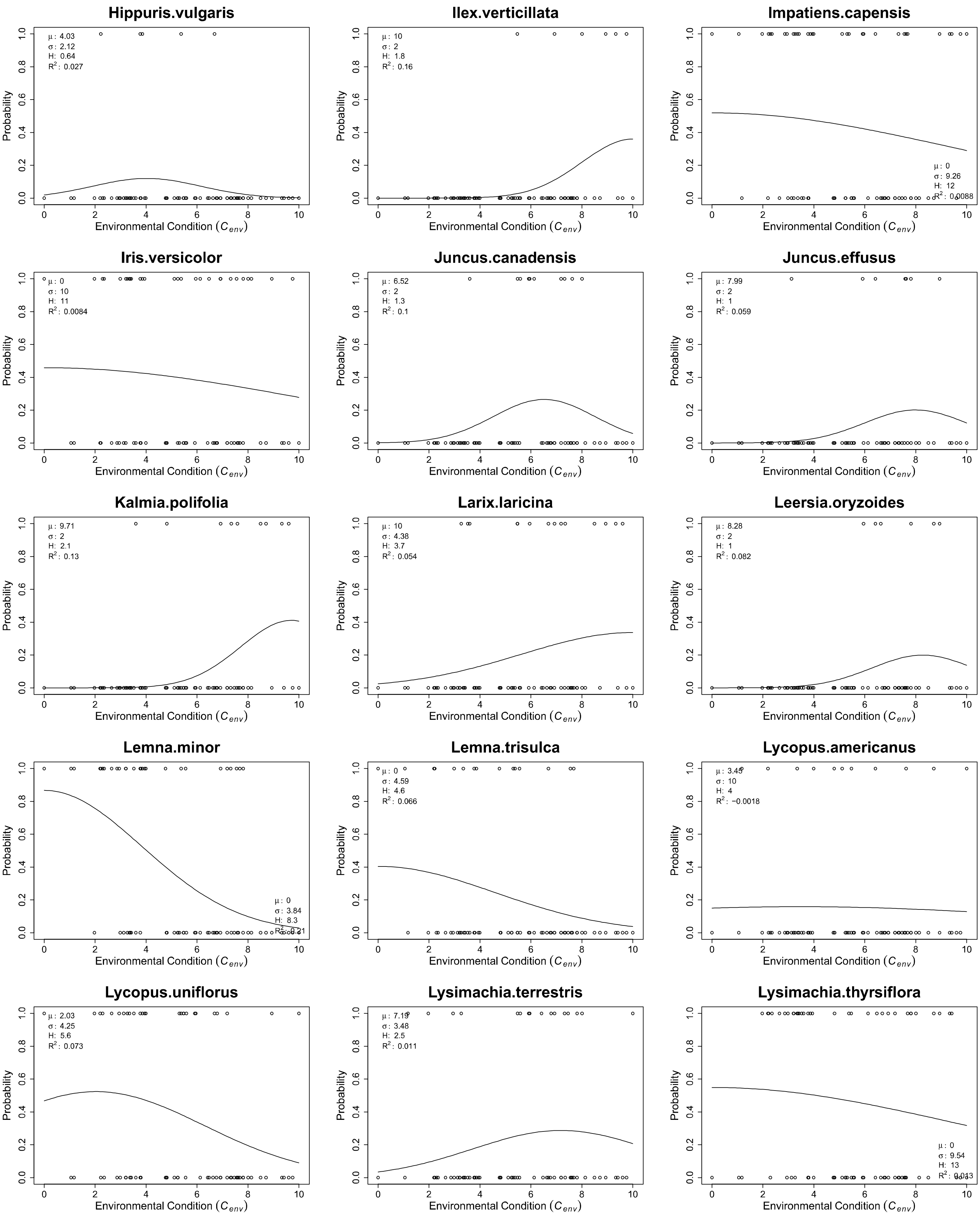

Hippuris.vulgaris
μ: 4.03
σ: 2.12
H: 0.64
R²: 0.027
Ilex.verticillata
μ: 10
σ: 2
H: 1.8
R²: 0.16
Impatiens.capensis
μ: 0
σ: 9.26
H: 12
R²: 0.0088
Iris.versicolor
μ: 0
σ: 10
H: 11
R²: 0.0084
Juncus.canadensis
μ: 6.52
σ: 2
H: 1.3
R²: 0.1
Juncus.effusus
μ: 7.99
σ: 2
H: 1
R²: 0.059
Kalmia.polifolia
μ: 9.71
σ: 2
H: 2.1
R²: 0.13
Larix.laricina
μ: 10
σ: 4.38
H: 3.7
R²: 0.054
Leersia.oryzoides
μ: 8.28
σ: 2
H: 1
R²: 0.082
Lemna.minor
μ: 0
σ: 3.84
H: 8.3
R²: 0.21
Lemna.trisulca
μ: 0
σ: 4.59
H: 4.6
R²: 0.066
Lycopus.americanus
μ: 3.45
σ: 10
H: 4
R²: −0.0018
Lycopus.uniflorus
μ: 2.03
σ: 4.25
H: 5.6
R²: 0.073
Lysimachia.terrestris
μ: 7.19
σ: 3.48
H: 2.5
R²: 0.011
Lysimachia.thyrsiflora
μ: 0
σ: 9.54
H: 13
R²: 0.013
Probability
Environmental Condition (C_{env})

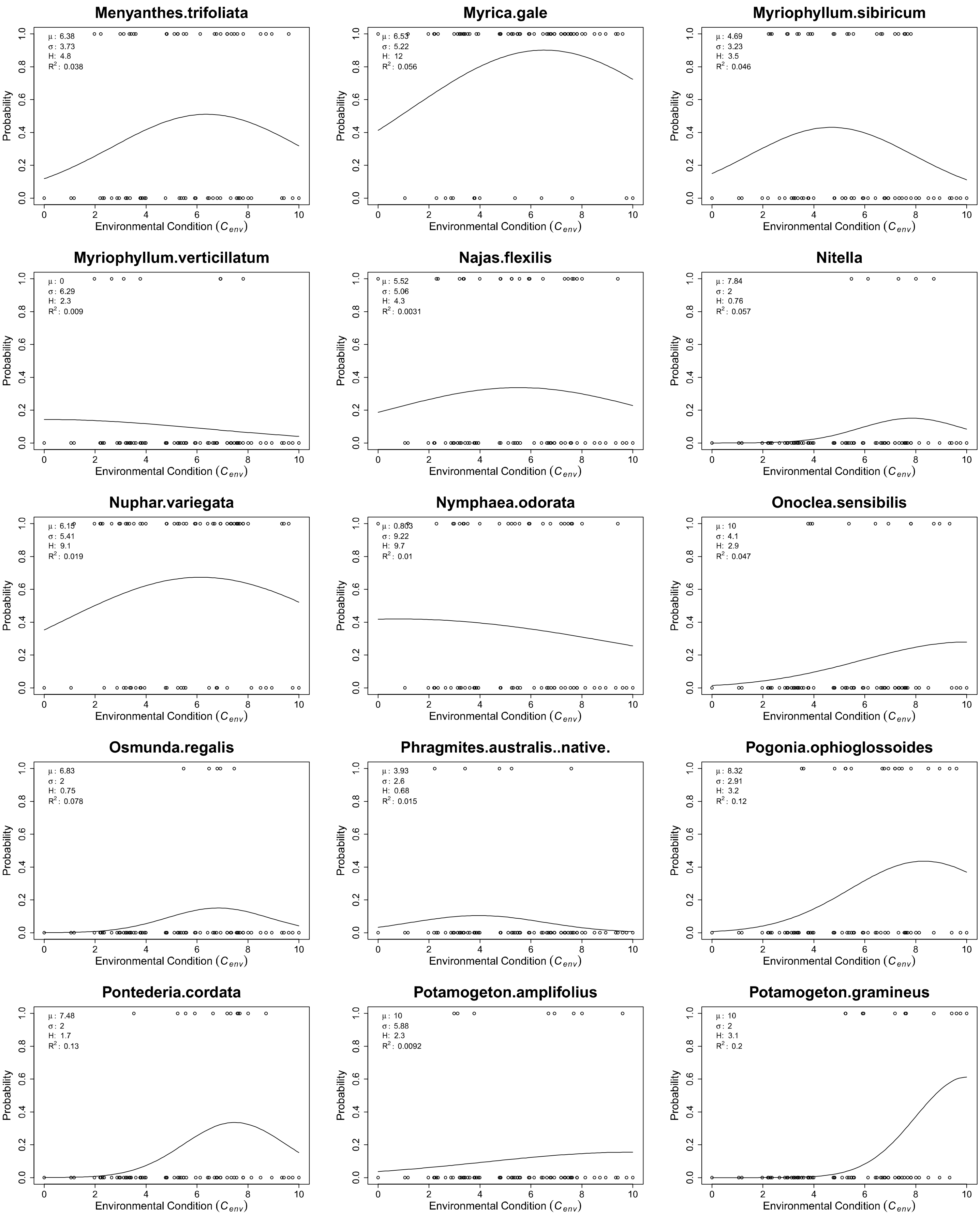
Menyanthes.trifoliata
μ: 6.38 σ: 3.73 H: 4.8 R^2: 0.038
Myrica.gale
μ: 6.53 σ: 5.22 H: 12 R^2: 0.056
Myriophyllum.sibiricum
μ: 4.69 σ: 3.23 H: 3.5 R^2: 0.046
Myriophyllum.verticillatum
μ: 0 σ: 6.29 H: 2.3 R^2: 0.009
Najas.flexilis
μ: 5.52 σ: 5.06 H: 4.3 R^2: 0.0031
Nitella
μ: 7.84 σ: 2 H: 0.76 R^2: 0.057
Nuphar.variegata
μ: 6.15 σ: 5.41 H: 9.1 R^2: 0.019
Nymphaea.odorata
μ: 0.803 σ: 9.22 H: 9.7 R^2: 0.01
Onoclea.sensibilis
μ: 10 σ: 4.1 H: 2.9 R^2: 0.047
Osmunda.regalis
μ: 6.83 σ: 2 H: 0.75 R^2: 0.078
Phragmites.australis..native.
μ: 3.93 σ: 2.6 H: 0.68 R^2: 0.015
Pogonia.ophioglossoides
μ: 8.32 σ: 2.91 H: 3.2 R^2: 0.12
Pontederia.cordata
μ: 7.48 σ: 2 H: 1.7 R^2: 0.13
Potamogeton.amplifolius
μ: 10 σ: 5.88 H: 2.3 R^2: 0.0092
Potamogeton.gramineus
μ: 10 σ: 2 H: 3.1 R^2: 0.2
Probability
Environmental Condition (C_{env})

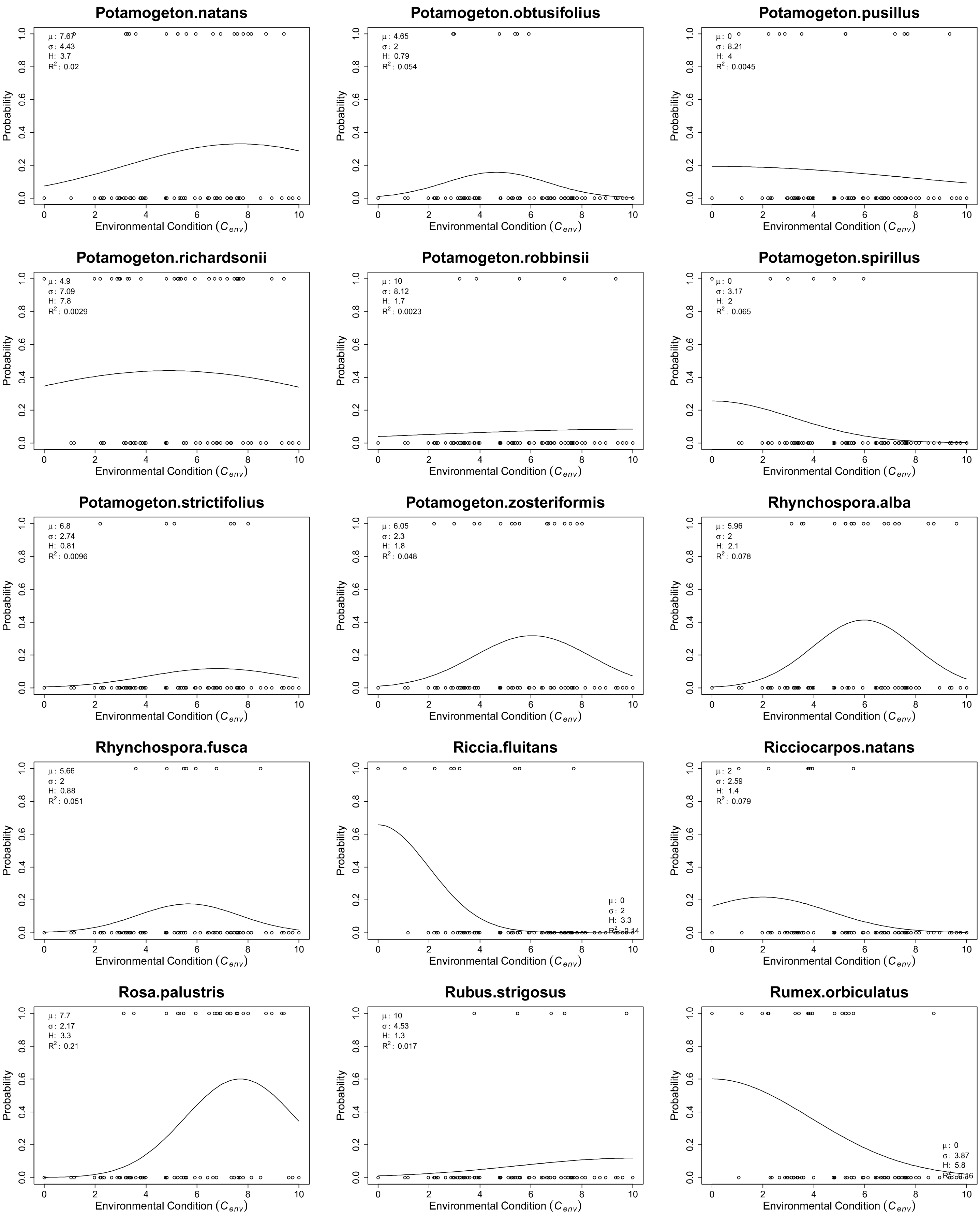

Potamogeton.natans
μ: 7.67
σ: 4.43
H: 3.7
R²: 0.02
Potamogeton.obtusifolius
μ: 4.65
σ: 2
H: 0.79
R²: 0.054
Potamogeton.pusillus
μ: 0
σ: 8.21
H: 4
R²: 0.0045
Potamogeton.richardsonii
μ: 4.9
σ: 7.09
H: 7.8
R²: 0.0029
Potamogeton.robbinsii
μ: 10
σ: 8.12
H: 1.7
R²: 0.0023
Potamogeton.spirillus
μ: 0
σ: 3.17
H: 2
R²: 0.065
Potamogeton.strictifolius
μ: 6.8
σ: 2.74
H: 0.81
R²: 0.0096
Potamogeton.zosteriformis
μ: 6.05
σ: 2.3
H: 1.8
R²: 0.048
Rhynchospora.alba
μ: 5.96
σ: 2
H: 2.1
R²: 0.078
Rhynchospora.fusca
μ: 5.66
σ: 2
H: 0.88
R²: 0.051
Riccia.fluitans
μ: 0
σ: 2
H: 3.3
R²: 0.14
Ricciocarpos.natans
μ: 2
σ: 2.59
H: 1.4
R²: 0.079
Rosa.palustris
μ: 7.7
σ: 2.17
H: 3.3
R²: 0.21
Rubus.strigosus
μ: 10
σ: 4.53
H: 1.3
R²: 0.017
Rumex.orbiculatus
μ: 0
σ: 3.87
H: 5.8
R²: 0.16
Probability
Environmental Condition (C_{env})

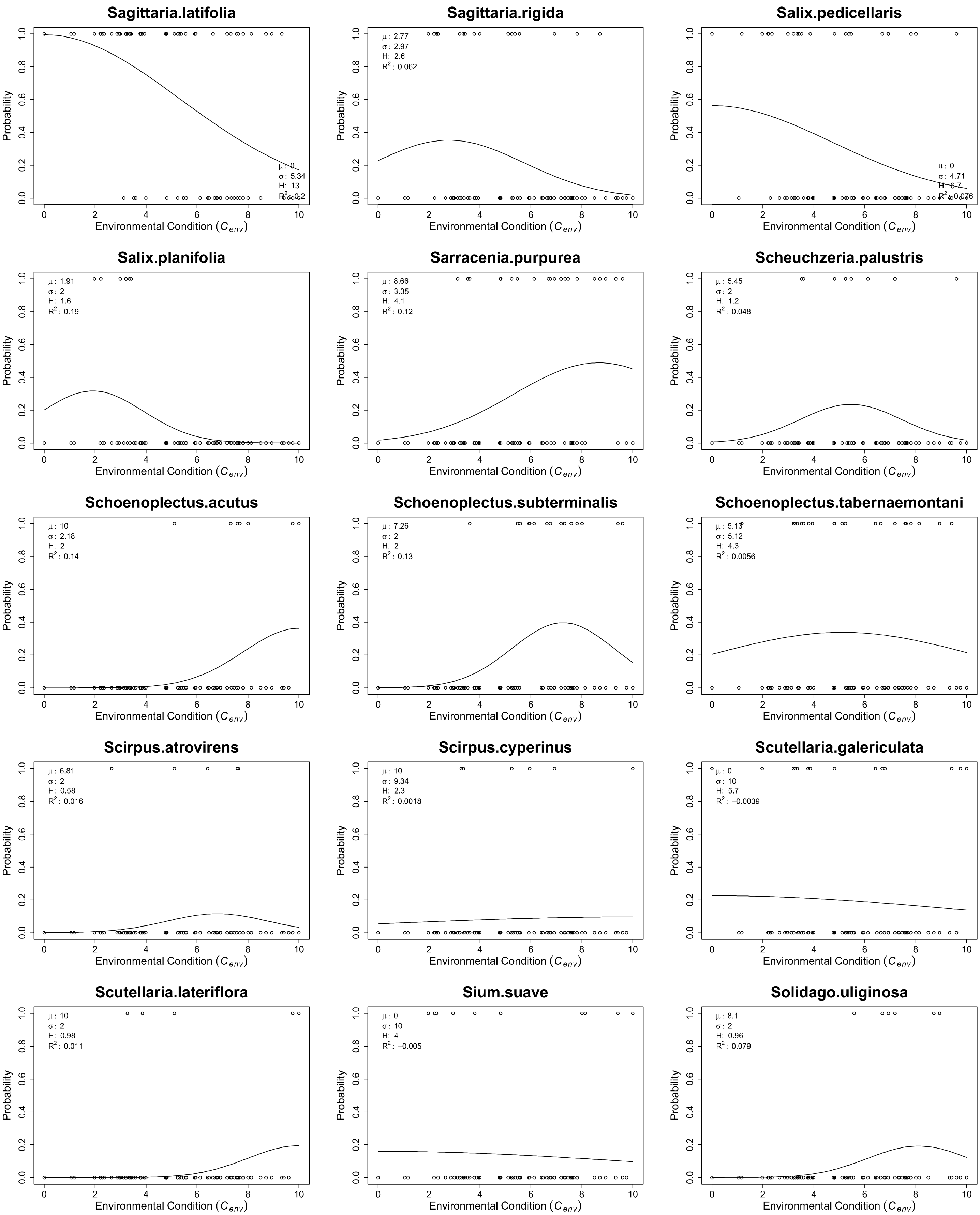

Sagittaria.latifolia
μ : 0
σ : 5.34
H: 13
R2 : 0.2
Sagittaria.rigida
μ : 2.77
σ : 2.97
H: 2.6
R2 : 0.062
Salix.pedicellaris
μ : 0
σ : 4.71
H: 6.7
R2 : 0.076
Salix.planifolia
μ : 1.91
σ : 2
H: 1.6
R2 : 0.19
Sarracenia.purpurea
μ : 8.66
σ : 3.35
H: 4.1
R2 : 0.12
Scheuchzeria.palustris
μ : 5.45
σ : 2
H: 1.2
R2 : 0.048
Schoenoplectus.acutus
μ : 10
σ : 2.18
H: 2
R2 : 0.14
Schoenoplectus.subterminalis
μ : 7.26
σ : 2
H: 2
R2 : 0.13
Schoenoplectus.tabernaemontani
μ : 5.13
σ : 5.12
H: 4.3
R2 : 0.0056
Scirpus.atrovirens
μ : 6.81
σ : 2
H: 0.58
R2 : 0.016
Scirpus.cyperinus
μ : 10
σ : 9.34
H: 2.3
R2 : 0.0018
Scutellaria.galericulata
μ : 0
σ : 10
H: 5.7
R2 : −0.0039
Scutellaria.lateriflora
μ : 10
σ : 2
H: 0.98
R2 : 0.011
Sium.suave
μ : 0
σ : 10
H: 4
R2 : −0.005
Solidago.uliginosa
μ : 8.1
σ : 2
H: 0.96
R2 : 0.079
Probability
Environmental Condition (Cenv)

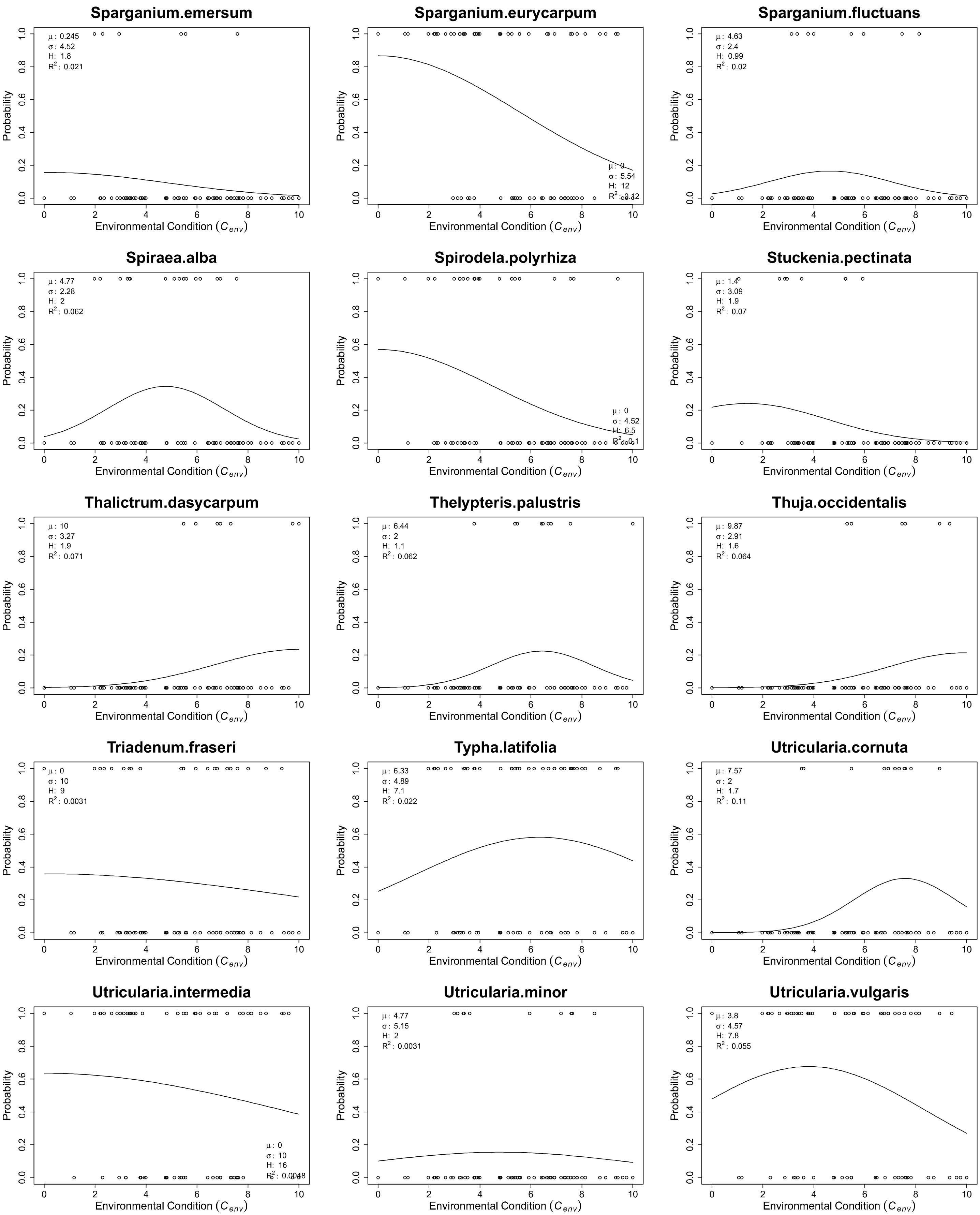

Sparganium.emersum
μ : 0.245
σ : 4.52
H: 1.8
R²: 0.021
Sparganium.eurycarpum
μ : 0
σ : 5.54
H: 12
Sparganium.fluctuans
μ : 4.63
σ : 2.4
H: 0.99
R²: 0.02
Spiraea.alba
μ : 4.77
σ : 2.28
H: 2
R²: 0.062
Spirodela.polyrhiza
μ : 0
σ : 4.52
H: 6.5
Stuckenia.pectinata
μ : 1.4
σ : 3.09
H: 1.9
R²: 0.07
Thalictrum.dasycarpum
μ : 10
σ : 3.27
H: 1.9
R²: 0.071
Thelypteris.palustris
μ : 6.44
σ : 2
H: 1.1
R²: 0.062
Thuja.occidentalis
μ : 9.87
σ : 2.91
H: 1.6
R²: 0.064
Triadenum.fraseri
μ : 0
σ : 10
H: 9
R²: 0.0031
Typha.latifolia
μ : 6.33
σ : 4.89
H: 7.1
R²: 0.022
Utricularia.cornuta
μ : 7.57
σ : 2
H: 1.7
R²: 0.11
Utricularia.intermedia
μ : 0
σ : 10
H: 16
Utricularia.minor
μ : 4.77
σ : 5.15
H: 2
R²: 0.0031
Utricularia.vulgaris
μ : 3.8
σ : 4.57
H: 7.8
R²: 0.055
Probability
Environmental Condition (C_{env})

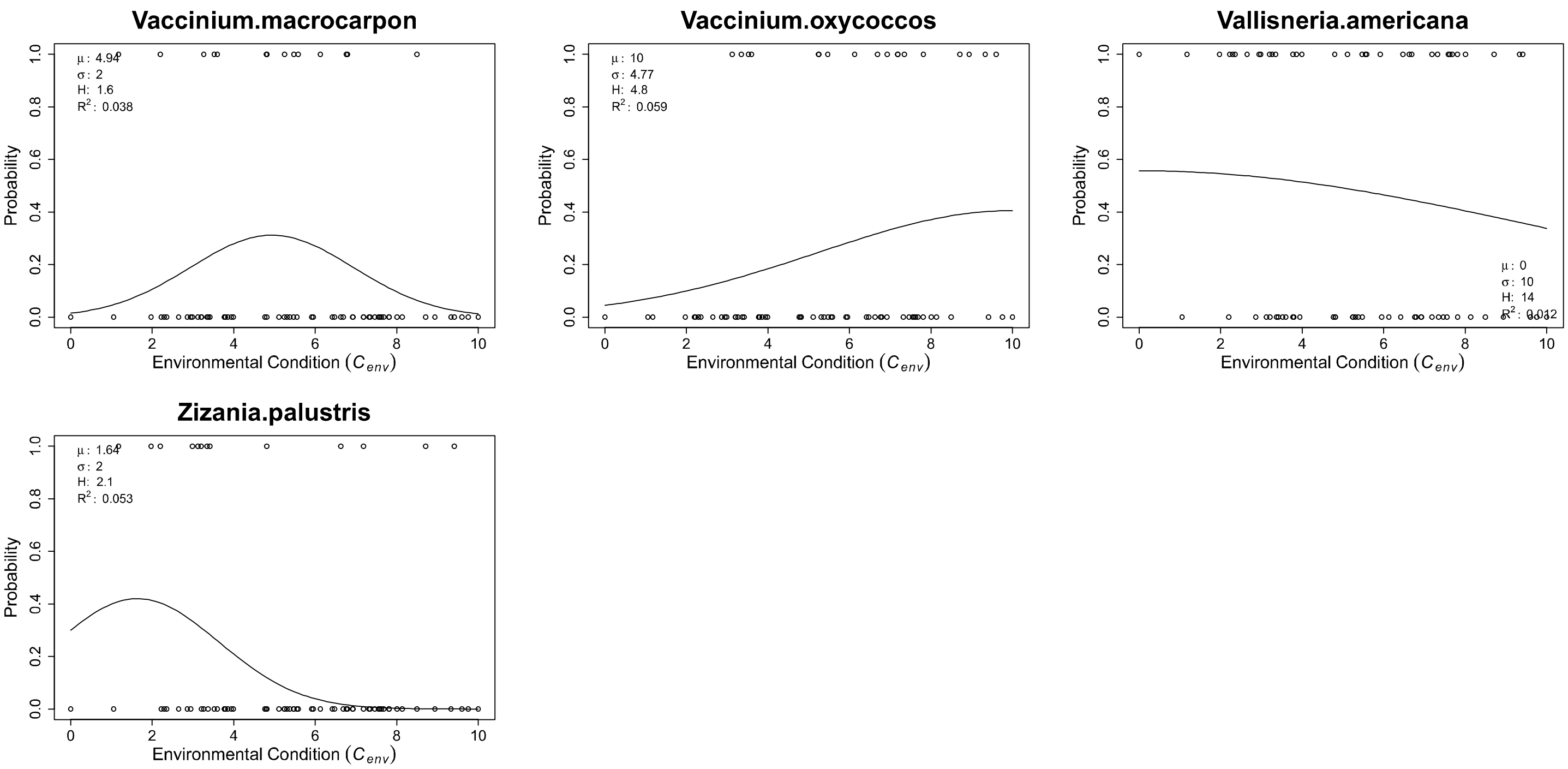
Vaccinium.macrocarpon
μ : 4.94
σ : 2
H: 1.6
R²: 0.038
Probability
Environmental Condition (C_env)
Vaccinium.oxycoccos
μ : 10
σ : 4.77
H: 4.8
R²: 0.059
Probability
Environmental Condition (C_env)
Vallisneria.americana
μ : 0
σ : 10
H: 14
R²: 0.012
Probability
Environmental Condition (C_env)
Zizania.palustris
μ : 1.64
σ : 2
H: 2.1
R²: 0.053
Probability
Environmental Condition (C_env)
