# Supplementary material for: Beyond Floristic Quality: A Data-Driven Approach to Assessing Coastal Wetland Condition Using Plants
Source: Wetlands (Wilmington). 2026 Oct 6;46(7):129. doi: 10.1007/s13157-026-02094-z (PMC13642451; doi:10.1007/s13157-026-02094-z)
Supplement: Supplementary file 6 [file 13157_2026_2094_MOESM6_ESM.pdf]

Wetlands

Online Resource 2: Results for water level and hydrogeomorphic type analyses for all Great Lakes

Beyond floristic quality: A data-driven approach to assessing coastal wetland condition using plants

Giuseppe E. Fiorino[1]*, Simon T. Denomme-Brown[1], Hayley N. Rogers[1], Ian M. Smith[1], Kathryn L. Amatangelo[2], Matthew J. Cooper[3], Nicholas P. Danz[4], Robert W. Howe[5], Rachel E. Schultz[2], Bridget A. Wheelock[6], Douglas A. Wilcox[2], Greg P. Grabas[1]

[1]Environment and Climate Change Canada, 4905 Dufferin Street, Toronto, Ontario M3H 5T4, Canada

[2]Department of Environmental Science and Ecology, SUNY-Brockport, 350 New Campus Drive, Brockport, NY 14420, USA

[3]Department of Biology, Grand Valley State University, Allendale, MI 49401, USA

[4]Department of Natural Sciences, University of Wisconsin-Superior, 1605 Catlin Avenue, Superior, WI 54880, USA

[5]Cofrin Center for Biodiversity, University of Wisconsin-Green Bay, Green Bay, WI 54311-7001, USA

[6]Institute for Great Lakes Research and Department of Biology, Central Michigan University, Mount Pleasant, MI, USA

*Corresponding Author (email: giuseppe.fiorino@ec.gc.ca, ORCID: 0000-0002-1569-0767)

Results

Water Levels and Hydrogeomorphic Types

We found no statistically significant interactions between any of the versions of the pIBC and annual water levels or wetland hydrogeomorphic type (Table 1), indicating the associations between different versions of the pIBC and WQLU were not significantly different between high- and low-water-level years, or among lacustrine, riverine, and barrier-protected hydrogeomorphic types (Figs. 1-5).

Table 1 Results from linear mixed-effects models used to test whether each lake-specific version of the plant-based Index of Biotic Condition (pIBC) was suitable for use under different water-level conditions, and for wetlands of different hydrogeomorphic types. Each model (one per Great Lake) had the Water Quality and Land Use (WQLU) Index as the response variable, two interaction terms (pIBC × annual water level [high or low] and pIBC × hydrogeomorphic type [HGM; lacustrine, riverine, or barrier-protected]) as fixed effects, and site and year as random effects. In all cases, there was a strong statistically significant positive association between pIBC and WQLU that was not significantly different between high- and low-water-level years, or among lacustrine, riverine, and barrier-protected hydrogeomorphic types. Given the inclusion of three-level categorical predictors in these models, we have reported results from Type III ANOVA tables to summarize the overall significance of fixed effects. Statistically significant *P*-values are indicated in boldface ($P < 0.05$)

Model	Effect	Sum Sq	Mean Sq	Num df	Den df	*F* value	*P* value
Lake Superior	$pIBC_{LS}$	2875.2	2875.2	1	102.6	40.3	**<0.001**
	Water Level	28.6	28.6	1	56.4	0.4	0.53
	HGM	7.5	3.7	2	89.7	0.1	0.95
	$pIBC_{LS}$ × Water Level	5.7	5.7	1	108.4	0.1	0.78
	$pIBC_{LS}$ × HGM	113.4	56.7	2	94.5	0.8	0.45

Lake Michigan	$pIBC_{LM}$	2071.9	2071.9	1	161.5	38.6	**<0.001**
	Water Level	0.1	0.1	1	55.9	0	0.96
	HGM	0	0	2	145.2	0	1
	$pIBC_{LM}$ × Water Level	10.7	10.7	1	92.9	0.2	0.66
	$pIBC_{LM}$ × HGM	42.4	21.2	2	169.9	0.4	0.67
Lake Huron	$pIBC_{LH}$	1665.7	1665.7	1	410.9	22.7	**<0.001**
	Water Level	0	0	1	81.8	0	1
	HGM	98.9	49.5	2	390.7	0.7	0.51
	$pIBC_{LH}$ × Water Level	0	0	1	259	0	0.98
	$pIBC_{LH}$ × HGM	24.2	12.1	2	420.2	0.2	0.85
Lake Erie	$pIBC_{LE}$	1713.1	1713.1	1	140.2	21.9	**<0.001**
	Water Level	181.6	181.6	1	34	2.3	0.14
	HGM	78	39	2	99	0.5	0.61
	$pIBC_{LE}$ × Water Level	73.2	73.2	1	95.6	0.9	0.34
	$pIBC_{LE}$ × HGM	94.9	47.4	2	108	0.6	0.55
Lake Ontario	$pIBC_{LO}$	3790.6	3790.6	1	284.2	56.2	**<0.001**
	Water Level	20.8	20.8	1	44.5	0.3	0.58
	HGM	89.4	44.7	2	234.4	0.7	0.52
	$pIBC_{LO}$ × Water Level	26.4	26.4	1	225.6	0.4	0.53
	$pIBC_{LO}$ × HGM	23	11.5	2	248.7	0.2	0.84

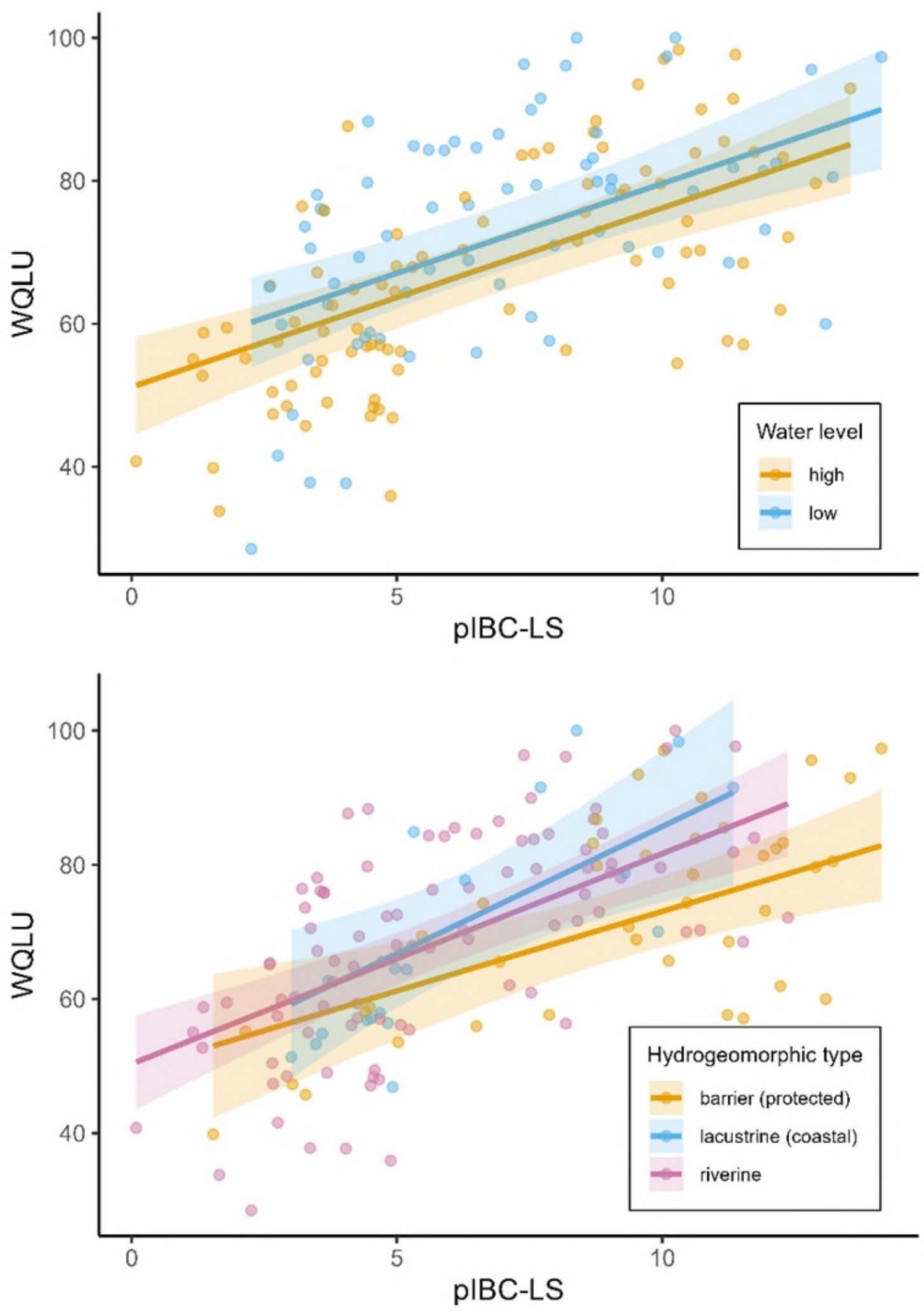

Fig. 1 Relationships between the Lake Superior version of the plant-based Index of Biotic Condition (pIBC$_{LS}$) and the Water Quality and Land Use (WQLU) Index, shown in high- versus low-water-level years (top), and for wetland hydrogeomorphic types (bottom). The association between pIBC$_{LS}$ and WQLU did not differ in high- versus low-water-level years, or among lacustrine, riverine, and barrier-protected hydrogeomorphic types (pIBC$_{LS}$ × annual water level and pIBC$_{LS}$ × hydrogeomorphic type interaction terms were not significantly significant). Points represent raw data. Lines and shaded ribbons represent model-predicted average trends and associated uncertainty (95% confidence intervals) based on a model that accounts for variation across sites and years

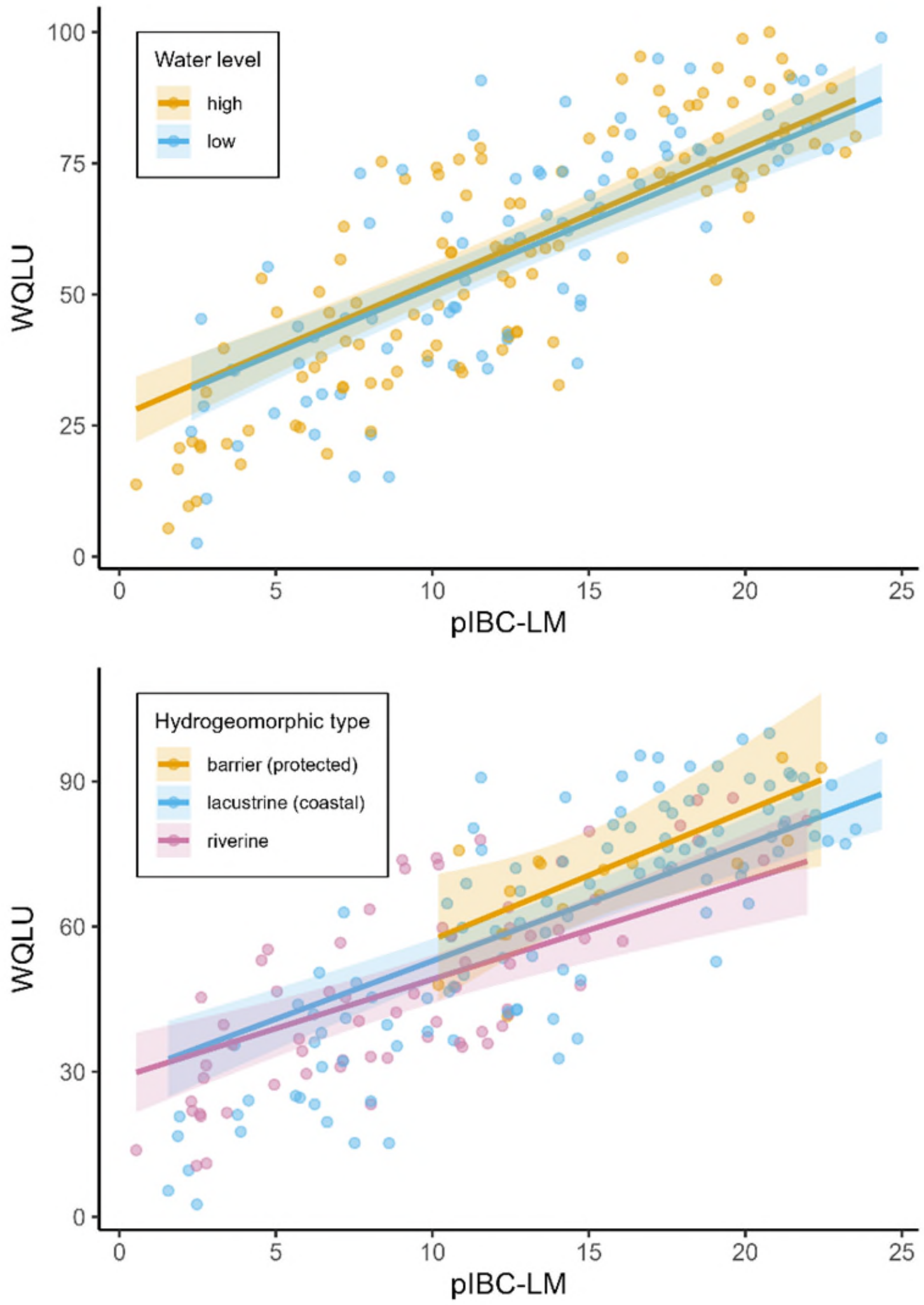

Fig. 2 Relationships between the Lake Michigan version of the plant-based Index of Biotic Condition ($pIBC_{LM}$) and the Water Quality and Land Use (WQLU) Index, shown in high- versus low-water-level years (top), and for wetland hydrogeomorphic types (bottom). The association between $pIBC_{LM}$ and WQLU did not differ in high- versus low-water-level years, or among lacustrine, riverine, and barrier-protected hydrogeomorphic types ($pIBC_{LM}$ × annual water level and $pIBC_{LM}$ × hydrogeomorphic type interaction terms were not significantly significant). Points represent raw data. Lines and shaded ribbons represent model-predicted average trends and associated uncertainty (95% confidence intervals) based on a model that accounts for variation across sites and years

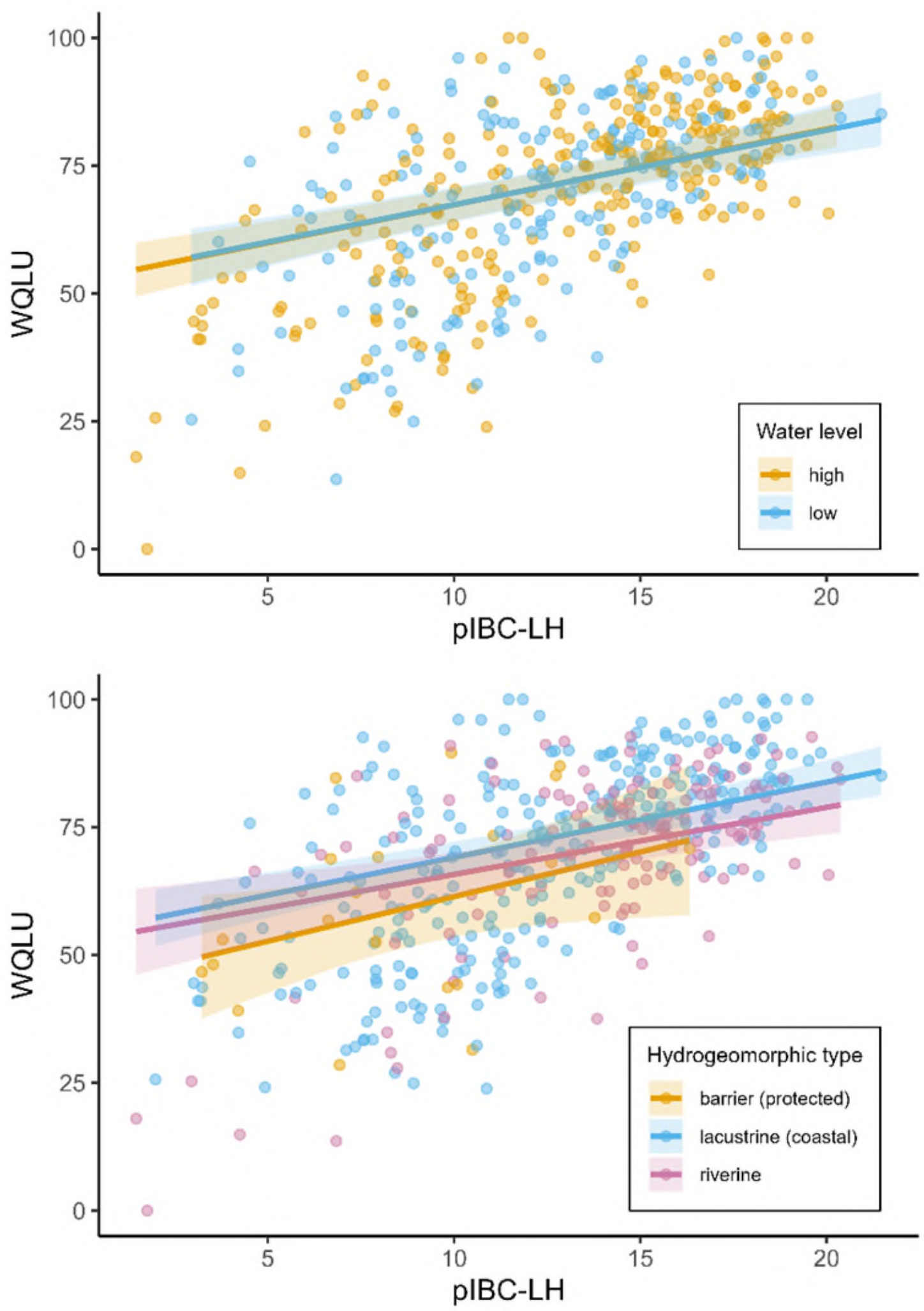

Fig. 3 Relationships between the Lake Huron version of the plant-based Index of Biotic Condition ($pIBC_{LH}$) and the Water Quality and Land Use (WQLU) Index, shown in high- versus low-water-level years (top), and for wetland hydrogeomorphic types (bottom). The association between $pIBC_{LH}$ and WQLU did not differ in high- versus low-water-level years, or among lacustrine, riverine, and barrier-protected hydrogeomorphic types ($pIBC_{LH}$ × annual water level and $pIBC_{LH}$ × hydrogeomorphic type interaction terms were not significantly significant). Points represent raw data. Lines and shaded ribbons represent model-predicted average trends and associated uncertainty (95% confidence intervals) based on a model that accounts for variation across sites and years

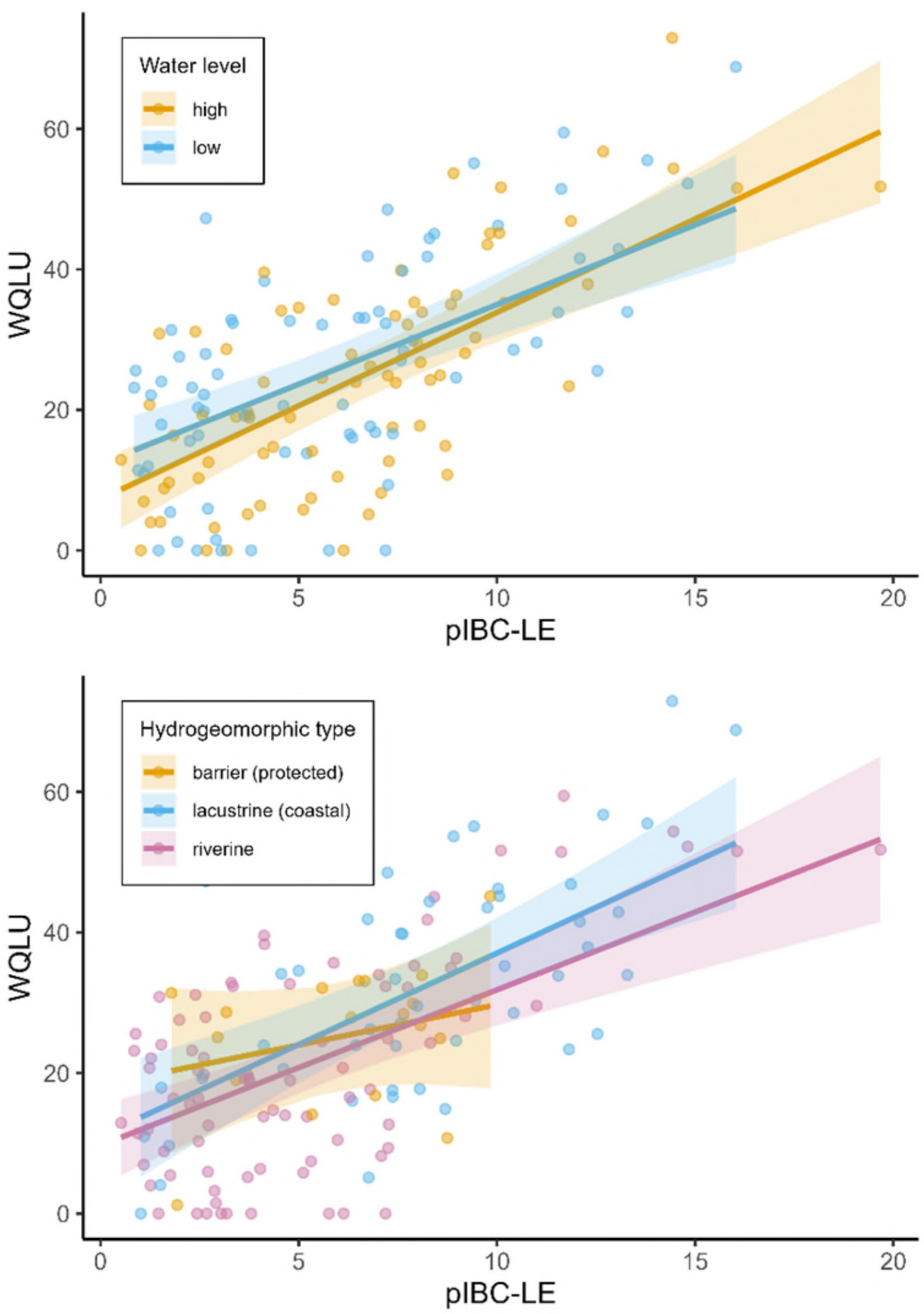

Fig. 4 Relationships between the Lake Erie version of the plant-based Index of Biotic Condition ($pIBC_{LE}$) and the Water Quality and Land Use (WQLU) Index, shown in high- versus low-water-level years (top), and for wetland hydrogeomorphic types (bottom). The association between $pIBC_{LE}$ and WQLU did not differ in high- versus low-water-level years, or among lacustrine, riverine, and barrier-protected hydrogeomorphic types ($pIBC_{LE}$ × annual water level and $pIBC_{LE}$ × hydrogeomorphic type interaction terms were not significantly significant). Points represent raw data. Lines and shaded ribbons represent model-predicted average trends and associated uncertainty (95% confidence intervals) based on a model that accounts for variation across sites and years

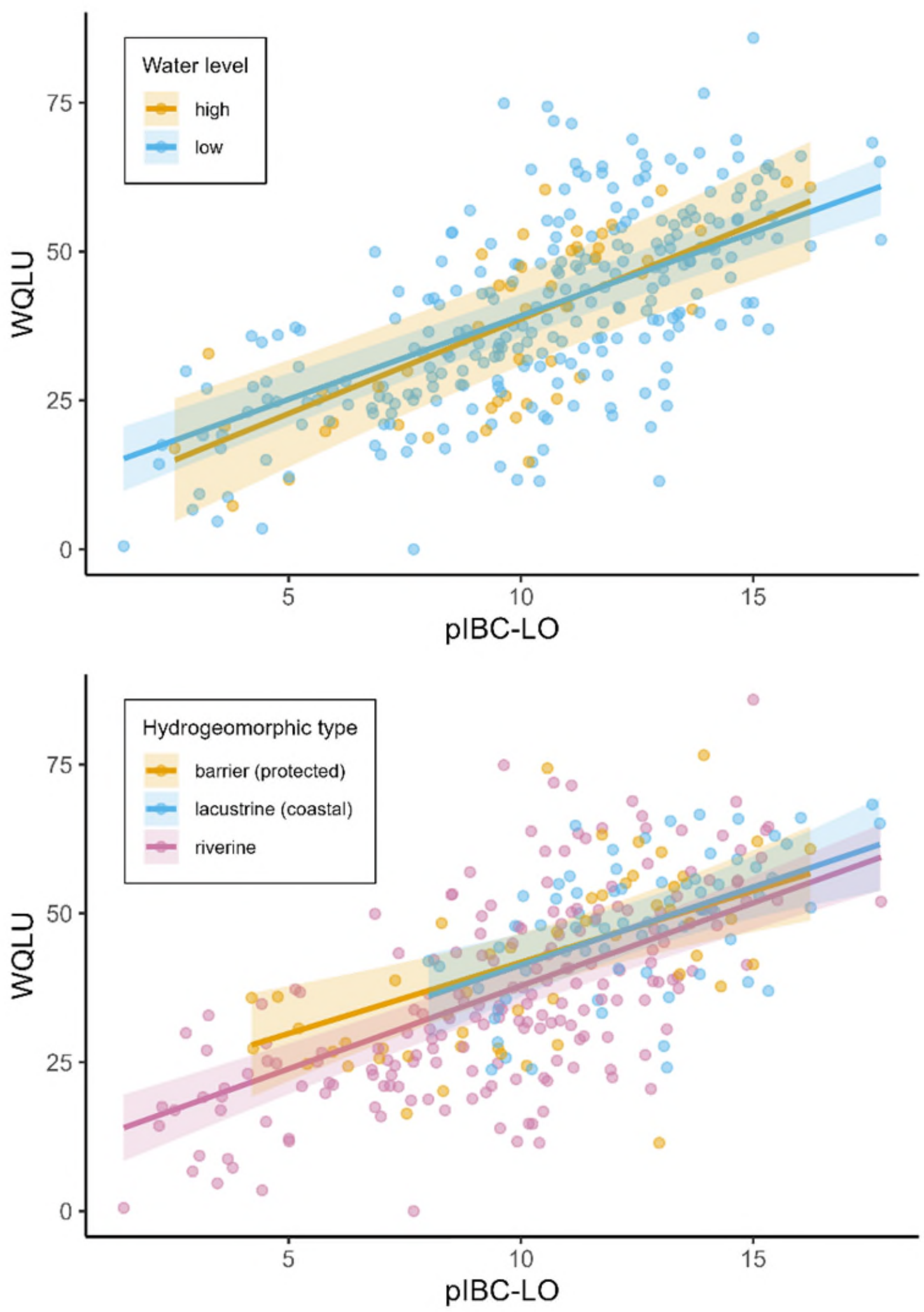

Fig. 5 Relationships between the Lake Ontario version of the plant-based Index of Biotic Condition ($pIBC_{LO}$) and the Water Quality and Land Use (WQLU) Index, shown in high- versus low-water-level years (top), and for wetland hydrogeomorphic types (bottom). The association between $pIBC_{LO}$ and WQLU did not differ in high- versus low-water-level years, or among lacustrine, riverine, and barrier-protected hydrogeomorphic types ($pIBC_{LO}$ × annual water level and $pIBC_{LO}$ × hydrogeomorphic type interaction terms were not significantly significant). Points represent raw data. Lines and shaded ribbons represent model-predicted average trends and associated uncertainty (95% confidence intervals) based on a model that accounts for variation across sites and years
