# Supplementary material for: Beyond Floristic Quality: A Data-Driven Approach to Assessing Coastal Wetland Condition Using Plants
Source: Wetlands (Wilmington). 2026 Oct 6;46(7):129. doi: 10.1007/s13157-026-02094-z (PMC13642451; doi:10.1007/s13157-026-02094-z)
Supplement: Supplementary file 7 [file 13157_2026_2094_MOESM7_ESM.pdf]

Wetlands

Online Resource 3: Standardized (Great Lakes) pIBC

Beyond floristic quality: A data-driven approach to assessing coastal wetland condition using plants

Giuseppe E. Fiorino[1]*, Simon T. Denomme-Brown[1], Hayley N. Rogers[1], Ian M. Smith[1], Kathryn L. Amatangelo[2], Matthew J. Cooper[3], Nicholas P. Danz[4], Robert W. Howe[5], Rachel E. Schultz[2], Bridget A. Wheelock[6], Douglas A. Wilcox[2], Greg P. Grabas[1]

[1]Environment and Climate Change Canada, 4905 Dufferin Street, Toronto, Ontario M3H 5T4, Canada

[2]Department of Environmental Science and Ecology, SUNY-Brockport, 350 New Campus Drive, Brockport, NY 14420, USA

[3]Department of Biology, Grand Valley State University, Allendale, MI 49401, USA

[4]Department of Natural Sciences, University of Wisconsin-Superior, 1605 Catlin Avenue, Superior, WI 54880, USA

[5]Cofrin Center for Biodiversity, University of Wisconsin-Green Bay, Green Bay, WI 54311-7001, USA

[6]Institute for Great Lakes Research and Department of Biology, Central Michigan University, Mount Pleasant, MI, USA

*Corresponding Author (email: giuseppe.fiorino@ec.gc.ca, ORCID: 0000-0002-1569-0767)

Background

One important feature of the pIBC is that the different versions were calibrated against the range of WQLU Index values for a given lake (where the highest values of pIBC are associated with the highest values of the WQLU Index for each lake). Calibrating pIBCs to be lake-specific is advantageous because it maximizes within-level resolution of the index, which is the scale that is often most relevant for conservation practitioners and land managers, but developing the index in this way means pIBC values cannot be directly compared among lakes because different lakes have different ranges of WQLU Index values (e.g., max WQLU Index value on Lake Erie was 72.9 whereas the max on Lake Huron was 100). There will, however, be instances where site comparisons among lakes may be required. To allow for comparison of pIBC values among lakes, we developed equations to standardize lake-specific pIBC values to the range of WQLU Index values for a given lake. However, users should interpret standardized pIBC values across lakes with caution, as these values reflect relative rankings within the original context of each lake's disturbance gradient (i.e., standardization aligns the numerical range of pIBC values across lakes but does not fully account for underlying differences in lake-specific drivers of disturbance and associated plant community response). Therefore, while standardized pIBC scores can support broad comparisons or regional summaries, the pIBC was developed primarily for detailed assessments of wetland condition within lakes.

Methods

We standardized lake-specific pIBC values to the range of WQLU Index values for a given lake (excluding outliers). Outliers were defined as WQLU values that were lower than the first quartile minus 1.5 times the interquartile range ($Q1-1.5\times IQR$) or higher than the third quartile

plus 1.5 times the interquartile range (Q3 +1.5×IQR). The standardized (Great Lakes) pIBC for a given wetland ($pIBC_{GL,i}$) was calculated following this general equation:

$$pIBC_{GL,i} = \left(\frac{pIBC_{Lake,i} - min\, pIBC_{Lake}}{max\, pIBC_{Lake} - min\, pIBC_{Lake}}\right)(max\, WQLU_{Lake} - min\, WQLU_{Lake}) + min\, WQLU_{Lake}$$

where $pIBC_{Lake,i}$ is the pIBC value for wetland i within a given lake;

$max\, WQLU_{Lake}$ and $min\, WQLU_{Lake}$ are the maximum and minimum WQLU values for all wetlands within a given lake; and

$max\, WQLU_{Lake}$ and $min\, WQLU_{Lake}$ were substituted for the upper or lower bound for outliers if $max\, WQLU_{Lake}$ or $min\, WQLU_{Lake}$ exceeded those boundaries.

The simplified equations for $pIBC_{GL,i}$ for each lake are:

$$pIBC_{GL,i} = 5.09\, pIBC_{LS,i} + 28.06 \text{ (Lake Superior)}$$

$$pIBC_{GL,i} = 4.09\, pIBC_{LM,i} + 0.38 \text{ (Lake Michigan)}$$

$$pIBC_{GL,i} = 3.81\, pIBC_{LH,i} + 18.29 \text{ (Lake Huron)}$$

$$pIBC_{GL,i} = 3.10\, pIBC_{LE,i} - 1.61 \text{ (Lake Erie)}$$

$$pIBC_{GL,i} = 5.27\, pIBC_{LO,i} - 7.65 \text{ (Lake Ontario)}$$

To assess the relationship between $pIBC_{GL}$ and the WQLU Index, we fit a linear mixed-effects model (Gaussian family) with the WQLU Index as the response variable, $pIBC_{GL}$ as a fixed effect, and site, year, and lake as random effects.

Results

Standardized $pIBC_{GL}$ values ranged from 28.5 to 100 for Lake Superior (mean: 62.2), 2.58 to 100 for Lake Michigan (mean: 51.0), 23.9 to 100 for Lake Huron (mean: 66.8), 0 to 59.5 for Lake Erie (mean: 17.6), and 0 to 85.9 for Lake Ontario (mean: 46.9) (Fig. 1). There was a strong,

statistically significant, positive association between $pIBC_{GL}$ and WQLU ($\beta \pm SE = 0.55 \pm 0.02$, $t_{960} = 22.05$, $P < 0.001$, $R^2_m = 0.36$) (Fig. 2).

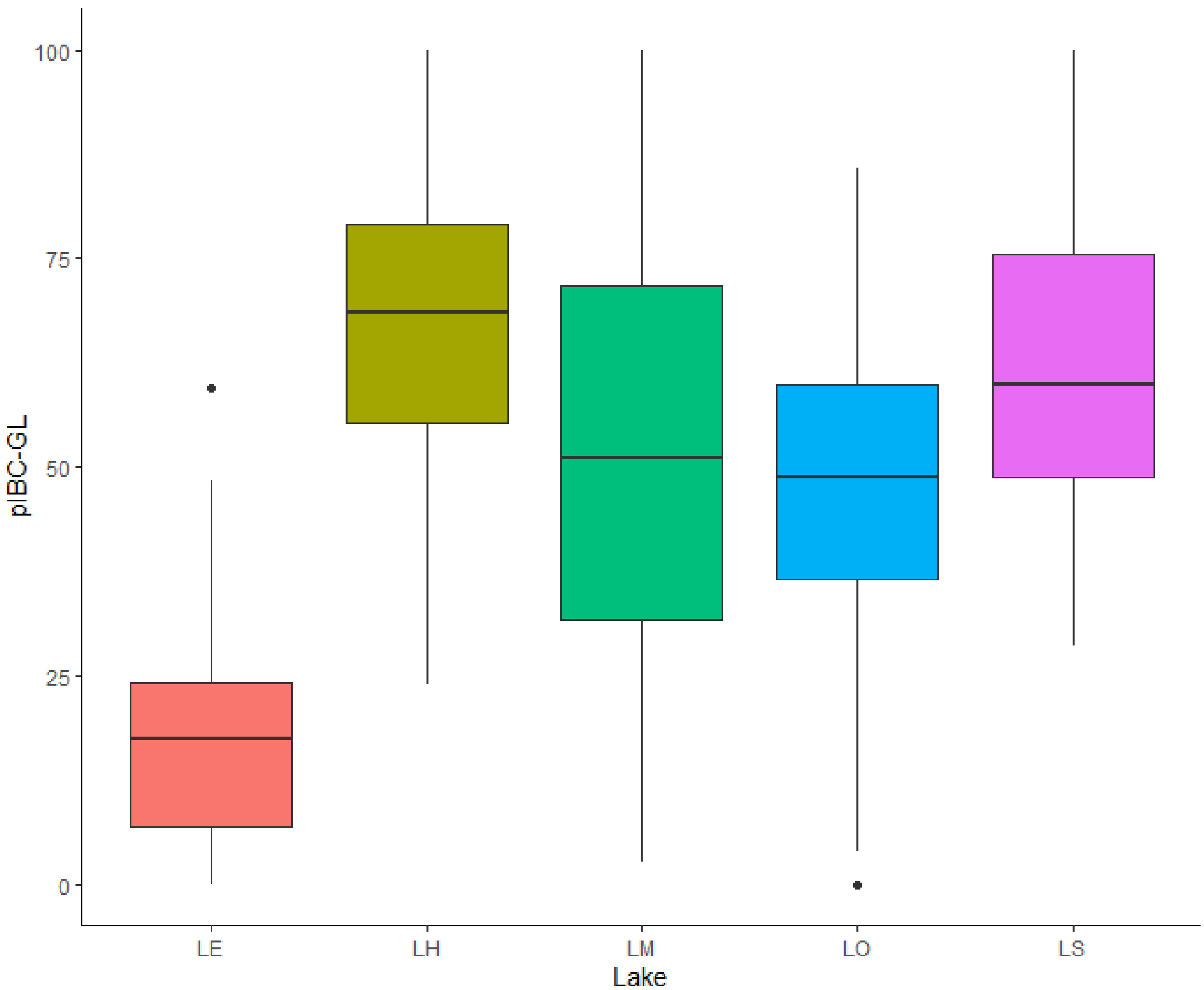

Fig. 1 Boxplots of standardized plant-based Index of Biotic Condition (pIBC-GL) values for coastal wetlands surveyed from 2011 to 2022 for each of the Laurentian Great Lakes

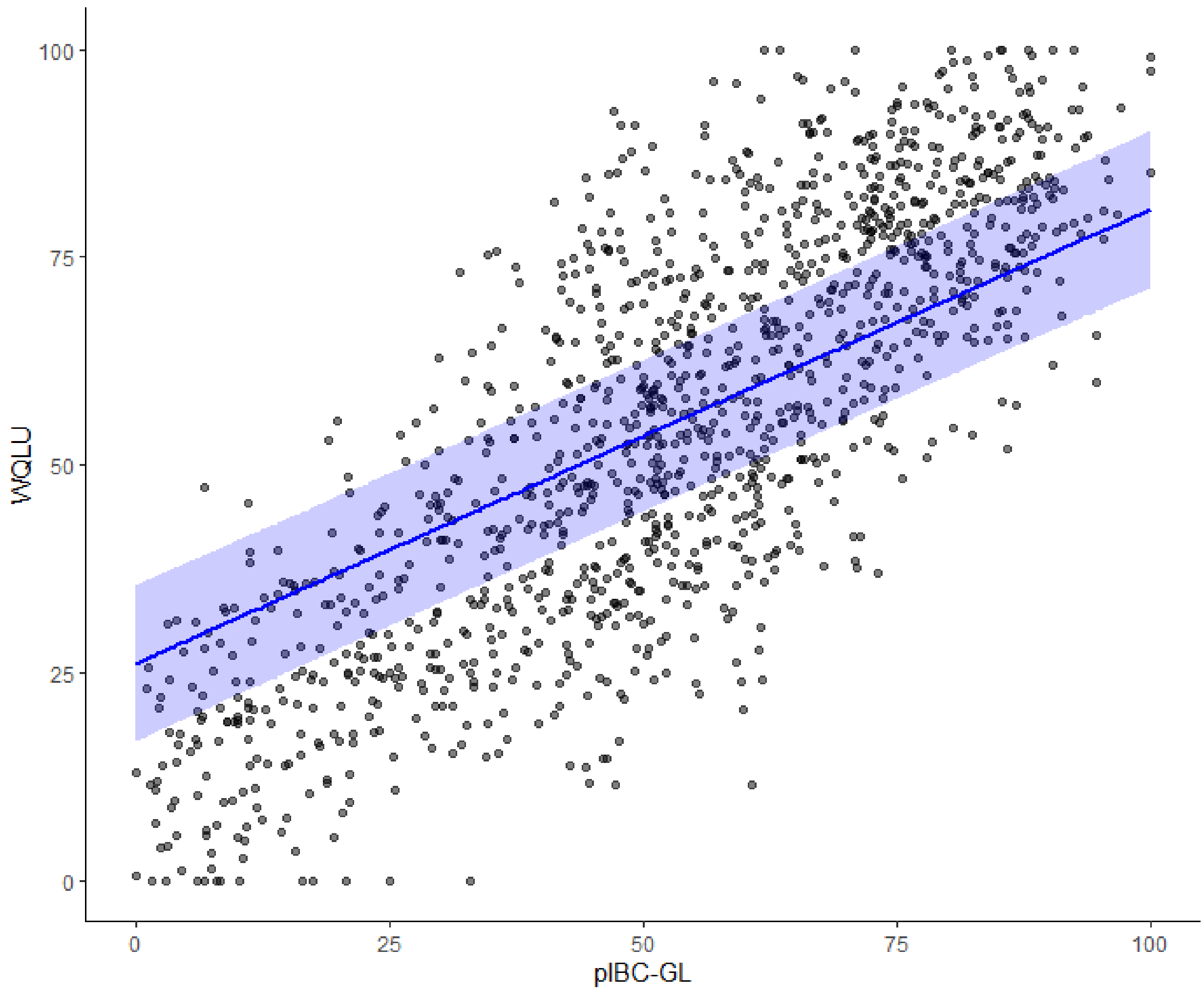

Fig. 2 Relationship between the standardized plant-based Index of Biotic Condition (pIBC-GL) and the Water Quality and Land Use (WQLU) Index for coastal wetlands surveyed from 2011 to 2022 for all of the Laurentian Great Lakes. There was a strong, statistically significant, positive association between $pIBC_{GL}$ and WQLU. Points represent raw data. Lines and shaded ribbons represent model-predicted average trends and associated uncertainty (95% confidence intervals) based on a model that accounts for variation across sites and years
